# “Do not leave me behind and do not put me in your box”: family-planning access and care needs among women with migration histories in Berlin, Germany—a mixed-methods study

**DOI:** 10.1186/s12978-026-02450-6

**Published:** 2026-09-25

**Authors:** Bahar Hassantabar, Gaotswake Patience Kovane, Nure Jannat Ara Riya, Namanou Ines Emma Woks, Modupe M. Fasina, Jalid Sehouli, Julia Leinweber, Angel Phuti

**Affiliations:** 1 Institute of International Health, Charité – Universitätsmedizin Berlin, Corporate Member of Freie Universität Berlin and Humboldt Universität zu Berlin, Berlin, Germany; 2 NuMIQ Research Focus Area, North-West University, Mahikeng, South Africa; 3 School of Public Health, Charité – Universitätsmedizin Berlin, Corporate Member of Freie Universität Berlin and Humboldt Universität zu Berlin, Berlin, Germany; 4 Clinic for Gynecology with Centre for Oncology, Charité – Universitätsmedizin Berlin, Corporate Member of Freie Universität Berlin and Humboldt Universität zu Berlin, Berlin, Germany; 5 Institute of Midwifery Science, Charité – Universitätsmedizin Berlin, Corporate Member of Freie Universität Berlin and Humboldt Universität zu Berlin, Berlin, Germany

**Keywords:** Family planning, Healthcare access, Health equity, Migration and health, Reproductive autonomy, Reproductive health, Germany, Mixed-methods research

## Abstract

**Introduction:**

Family planning is a fundamental human right and contributes to several Sustainable Development Goals. Women who have migrated from low- and middle-income countries may encounter barriers to family planning and reproductive healthcare. This study examined access to family planning information and services and related care needs among first-generation women with migration histories in Berlin, Germany.

**Methods:**

We conducted a qualitatively driven, emergent sequential mixed-methods study with 29 first-generation women who had migrated from low- and middle-income countries to Berlin and accessed maternal or reproductive healthcare services within the preceding two years. Semi-structured interviews were conducted between 17 July and 12 November 2024, analyzed thematically, and mapped to the Education, Training, and Research (ETR) Health Equity Framework. Selected codes were organized in a participant-by-code matrix for descriptive cross-case comparison. Findings were integrated through cross-case comparison and conceptual-model development.

**Findings:**

Four care-need domains were identified: communication and information; continuity and affordability; respect and shared responsibility; and cultural and life-course responsiveness. Access was shaped through four pathways: Systems of Power, Psychological Pathways, Individual Pathways, and Relationships and Networks. Structural, linguistic, financial, psychological, and relational conditions constrained access, while individual agency, peer and community networks, and navigation support could facilitate it. Barriers and facilitators co-existed.

**Conclusion:**

Equitable family planning access requires more than formal service availability. Health systems should provide affordable, understandable, continuous, respectful, responsive care that supports informed choice and reproductive autonomy while reducing structural, linguistic, financial, and administrative barriers. Agency and community support should complement, not substitute for, health-system responsibility.

**Supplementary Information:**

The online version contains supplementary material available at https://doi.org/10.1186/s12978-026-02450-6.

## Introduction

 Universal access to family planning is widely recognized as a human right and is central to gender equality and women’s empowerment, reflecting states’ obligations under international human rights law [1, 2]. This rights-based perspective is embedded in the approach of the United Nations Population Fund (UNFPA) to family planning, which emphasizes bodily autonomy and agency, informed decision-making, non-discrimination, equality, participation, and quality of care across the individual, service delivery, community, and policy levels [3]. It also aligns with ecosystem-based approaches in high-income countries that position family planning as a shared responsibility of health systems and public policy rather than solely as an individual responsibility [3, 4].

A rights-based approach is particularly important for people who have migrated, including those seeking refuge or asylum, who should be recognized as equal partners and rights holders within healthcare systems [5]. Equitable access to family planning information and services benefits people with migration histories as well as the communities and health systems in which they live and contributes to several Sustainable Development Goals, particularly those related to gender equality, health, and well-being [5, 6]. However, the formal availability of services does not necessarily ensure that care is affordable, understandable, respectful, continuous, or responsive to women’s circumstances [7, 8].

Germany is a high-income country with a well-resourced health system and relatively low levels of unmet need for family planning in the general population. In 2025, unmet need for family planning among women aged 15–49 was estimated at 6% among all women and 9% among women who were married or in a union [9]. Definitions of unmet need vary according to the population and indicator used; among women who are married or in a union, it is commonly defined as the proportion of women of reproductive age who wish to delay or avoid pregnancy but are not using contraception [10]. Evidence indicates that unmet need is substantially higher among women with refugee status and women seeking asylum in Germany [10, 11]. A 2020 study estimated unmet need for family planning at 47% among this population [11]. Compared with the UNFPA population-level estimate of approximately 6%–9%, this figure is roughly five to eight times as high [9, 11].

Across different national and population contexts, unmet need for family planning has been associated with unintended pregnancy [12–16]. By increasing the likelihood of unintended or inadequately spaced pregnancies, including pregnancies that may end in abortion under unsafe conditions, unmet need may contribute to preventable maternal morbidity and mortality [17–20].

It is therefore an important indicator of reproductive health outcomes and health-system performance [21]. Given the markedly higher levels reported among women with refugee status and women seeking asylum in Germany, understanding the factors that contribute to unmet need for family planning in this population is particularly important.

In Germany, the term *women with migration histories*, adapted from the German *Menschen mit Migrationsgeschichte*, is increasingly used as a more inclusive and less label-based alternative to terms such as *Ausländer* or *Migrantin/Migrant* [22–24]. In population statistics, this category may include both first- and second-generation women. Women with migration histories comprise approximately 38.4% of the female population in Germany and nearly half of women in key reproductive-age groups [25, 26]. The present study focused specifically on first-generation women who migrated to Germany from low- and middle-income countries (LMICs). This group was selected because research indicates that first-generation women may encounter multiple barriers that influence reproductive health outcomes and access to healthcare [27, 28]. Moreover, as the population of first-generation women with migration histories in Germany continues to grow, understanding their family planning care needs and access to relevant information and services is increasingly important [25, 26].

Research conducted in Germany has provided important evidence on family planning and sexual and reproductive health among people with migration histories; however, substantial gaps remain. The *Frauen Leben* series examined family planning in the general population and among women with migration histories [29–31]. Within the series, *Frauen Leben 2* included a survey of 1,674 women aged 20–44 years with Turkish or Eastern European migration histories living in Berlin, Stuttgart, Nuremberg, and Oberhausen [31]. Although this work made an important contribution, its focus on selected subgroups and relatively established communities with migration histories, together with data collected in 2007 and 2010, limits its applicability to increasingly diverse migration patterns and the current healthcare context in Germany. The study also provided limited insight into women’s care needs and pathways to accessing family planning services.

The German Health and Sexuality Survey provided more recent population-level evidence through a nationally representative study of 4,955 adults conducted in 2018–2019, including participants with first- and second-generation migration histories [32]. Participants in both generations reported greater unmet needs, particularly for contraceptive counselling, as well as more frequent barriers related to language and experiences of shame. However, the study examined a broad adult population and a wide range of sexual health topics rather than investigating family planning access in depth among first-generation women who had migrated from LMICs. Similarly, Inci et al. [11]. conducted a cross-sectional study among women with refugee status and women seeking asylum and reported a high level of unmet need for family planning despite formal access to services. However, unmet need is a population-level indicator and does not explain why access may be inadequate or capture broader dimensions of care, including information, affordability, continuity, communication, shared decision-making, and responsiveness to individual circumstances [7, 8]. An in-depth examination of family planning access is therefore needed to understand women’s ability to obtain appropriate information and services and to make informed contraceptive choices [33, 34].

Existing research demonstrates disparities, but a key remaining gap is explanatory. Current evidence provides limited understanding of why barriers persist, how women experience them, and how migration-related, structural, relational, psychological, and health-system factors interact to shape access to family-planning care. Much of the available research has focused on specific groups, particularly women with refugee status and women seeking asylum, or on other health outcomes such as mental health; although these studies provide valuable insights, they offer limited evidence on family-planning and reproductive-care experiences [35, 36]. Less is known about the experiences of first-generation women with diverse migration pathways, including migration for family reunification, education, employment, and protection.

This study therefore examined access to family planning and related care needs among first-generation women who had migrated from LMICs and were living in Berlin. Specifically, it aimed to (1) explore participants’ family planning experiences and care needs and (2) identify barriers to and facilitators of access to family planning information and services. The study sought to generate recommendations for strengthening equitable access, reproductive autonomy, informed choice, and health-system responsiveness in Germany, with potential relevance to other high-income settings with increasingly diverse populations shaped by migration.

## Methods

### Study design and setting

**Fig. 1:**
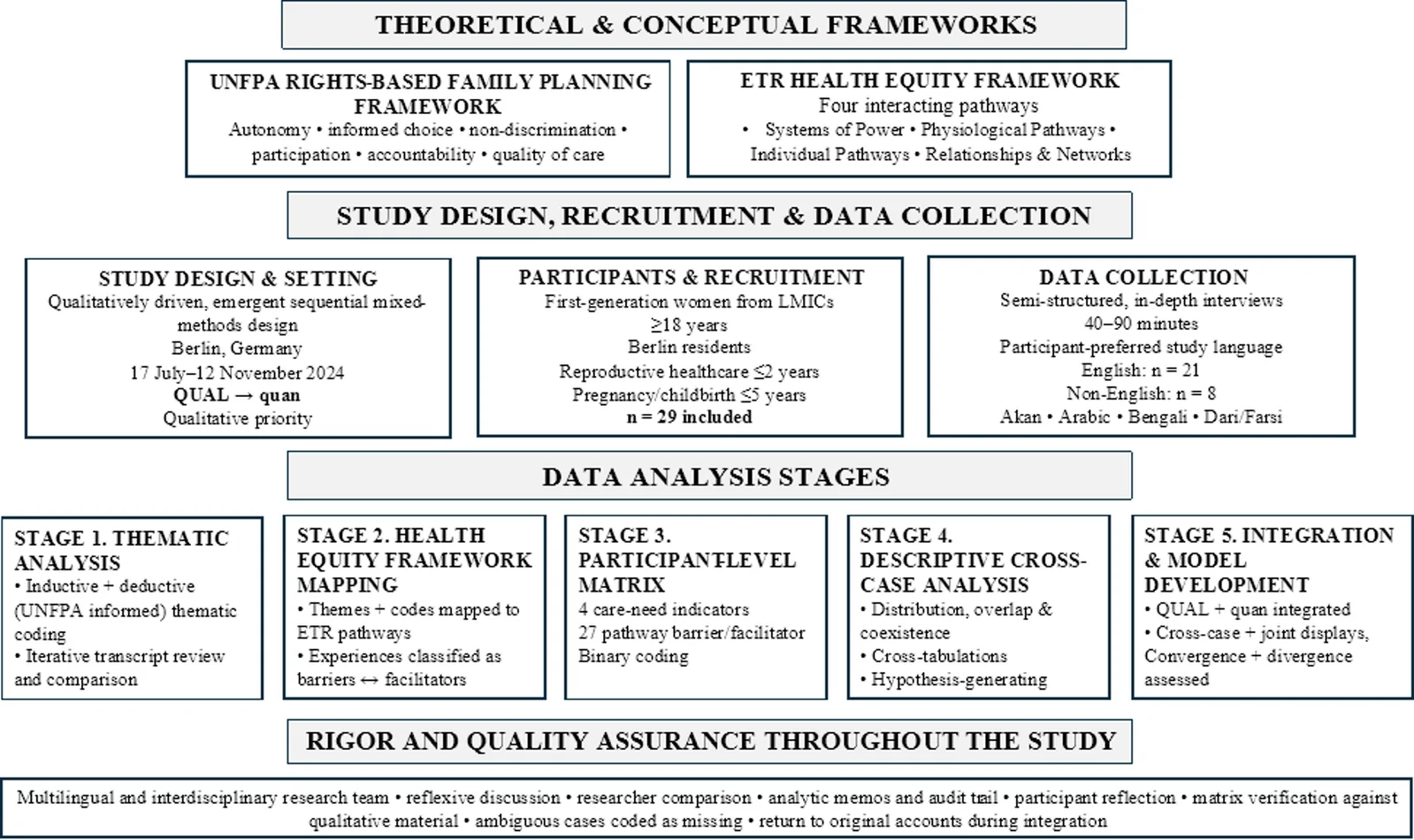
Qualitatively driven, emergent sequential mixed-methods design and analytical sequence of the Family Planning and Reproductive Healthcare Access Study, Berlin, Germany, 2024. Note. The qualitative component had methodological priority and informed the subsequent descriptive cross-case analysis. Following thematic analysis and Health Equity Framework mapping, selected qualitative codes were transformed into participant-level variables for descriptive cross-case analysis. Qualitative and descriptive findings were integrated through cross-case comparison, joint displays, narrative weaving, and return to the original interview accounts to inform conceptual-model development. QUAL denotes the dominant qualitative component and quan the subordinate descriptive quantitative component

This study used a qualitatively driven, emergent sequential mixed-methods design. Semi-structured interviews constituted the exploratory and descriptive qualitative core and examined participants’ family planning experiences, care needs, and barriers to and facilitators of accessing family planning information and services [37]. The qualitative component had methodological priority and informed the subsequent descriptive cross-case analysis. The study adopted a pragmatic research paradigm, with an interpretive orientation guiding the qualitative component. Pragmatism supported the use and integration of qualitative and descriptive quantitative approaches according to their contribution to the research questions, while the interpretive orientation emphasized participants’ meanings, experiences, and contexts. Findings from the qualitative analysis generated and refined the codes that were subsequently organized as participant-level variables in a participant-by-code matrix to support systematic comparison across cases [38, 39]. The overall study design, data-collection process, analytical sequence, and mixed-methods integration are summarized in Fig. 1.

The study was conducted in Berlin, Germany, between 17 July and 12 November 2024. Berlin was selected as a diverse migrant-receiving urban setting in which women with different countries of origin, migration pathways, residence histories, and reproductive health experiences could be recruited.

### Theoretical and conceptual frameworks

The study was informed by the United Nations Population Fund (UNFPA) rights-based family planning framework and the Education, Training, and Research (ETR) Health Equity Framework [3, 40]. The UNFPA framework provided a normative basis for examining family planning as a reproductive health and human rights issue, with attention to bodily autonomy, informed and voluntary decision-making, non-discrimination, participation, accountability, and quality of care.

The ETR Health Equity Framework provided an analytical structure for examining how access to care and reproductive-health experiences were shaped through four interrelated pathways: Systems of Power, Physiological Pathways, Individual Pathways, and Relationships and Networks [40]. Systems of Power encompassed structural and institutional conditions such as discrimination, healthcare entitlements, financial constraints, and material circumstances. The original Physiological Pathways sphere encompasses biological, physical, cognitive, and psychological processes through which social and environmental conditions may influence health [40]. In the present study, empirical material relevant to this sphere concerned predominantly psychological and emotional processes rather than biological or physiological mechanisms. We therefore retained the conceptual placement of these experiences within the original Physiological Pathways sphere but operationalized the domain as Psychological Pathways to reflect the narrower empirical focus of the data. This study-specific adaptation encompassed trauma-related fear, emotional distress, reproductive loss, displacement, painful memories, and psychological safety and should not be interpreted as a proposed modification of the ETR Health Equity Framework itself.

Within Psychological Pathways, barriers were defined as psychologically constraining conditions relevant to participants’ reproductive-health experiences, decision-making, communication, or engagement with care. The presence of trauma, distress, or mental-health concerns alone was not considered sufficient; coding required evidence of a meaningful psychological mechanism within the reproductive-health context, and ambiguous or purely descriptive accounts were not classified as barriers. Accordingly, these variables were interpreted as psychologically constraining conditions rather than evidence that psychological distress independently caused a particular family-planning decision, healthcare behaviour, or access outcome. Individual Pathways included health literacy, language, contraceptive knowledge, information seeking, decision-making confidence, and self-advocacy. Relationships and Networks encompassed partners, families, peers, community organizations, digital networks, practical support, social isolation, and stigma.

The UNFPA and ETR frameworks were used as complementary sensitizing and organizing tools rather than as a prespecified causal model. They informed interview-guide development, deductive coding, framework mapping, the organization of barriers and facilitators, construction of the participant-by-code matrix, mixed-methods integration, and conceptual-model development. Inductive coding was retained throughout to allow experiences and care needs not anticipated by either framework to emerge from participants’ accounts. The resulting pathway classifications were therefore interpretive and descriptive and were not intended to establish causal relationships between pathway conditions and family-planning access.

### Study population and eligibility criteria

The family-planning analysis was nested within a broader study of maternal and reproductive healthcare experiences among women with migration histories in Germany, titled Migrants’ Maternal Health in Germany (MIMAHG), and used the same interview dataset. Eligibility criteria were established for the parent study.

For the present analysis, *women with migration histories* was operationally defined more narrowly than the broader population-level German term. Eligible participants were first-generation women who were born outside Germany, had migrated to Germany from a low- or middle-income country (LMIC), and were residing in Berlin at the time of recruitment. Participants were required to be aged 18 years or older, to have accessed maternal or reproductive healthcare services in Germany within the preceding two years, to have experienced at least one pregnancy or childbirth within the preceding five years, and to be able and willing to provide informed consent.

Women were excluded if they did not meet one or more of these predefined eligibility criteria. Three women who contacted the research team were excluded because they had migrated from high-income countries and therefore did not meet the study eligibility criteria. The two participants who completed pilot interviews were excluded from the final analytical dataset. The final analytical sample comprised 29 participants.

### Recruitment and sampling strategy

Participants were recruited in Berlin between 17 July and 12 November 2024 using voluntary-response recruitment followed by eligibility-based purposive screening. Recruitment sought variation in migration pathway, country of origin, duration of residence in Germany, and reproductive health experiences rather than statistical representativeness.

Recruitment information was disseminated through community organizations, nongovernmental organizations, social media channels, community networks, and multilingual study flyers. Community partners included the African Parents Association Berlin-Brandenburg (AfEBB e.V.) and United Action Women and Girls e.V. Recruitment materials provided a brief description of the study, eligibility criteria, information about the voluntary nature of participation, and contact details for the research team.

Community organizations supported dissemination of the study information but were not involved in participant selection. Interested women contacted the research team directly and were screened by a member of the study team against the predefined eligibility criteria before an interview was arranged. This process was designed to minimize unnecessary disclosure of participation to community organizations and to safeguard voluntariness, privacy, and confidentiality.

Recruitment materials were written in accessible language and reviewed for clarity and cultural sensitivity by members of the multilingual and interdisciplinary research team. Examples of the recruitment materials are provided in Supplementary File 1.

Interviews were conducted in locations selected in consultation with participants to promote privacy, confidentiality, accessibility, and comfort. These included the African Parents Association Berlin-Brandenburg (AfEBB e.V.), United Action Women and Girls e.V., private rooms at the Institute of International Health at Campus Virchow, participants’ homes when preferred, and secure online platforms.

### Interview guide development and data collection

Data were collected through semi-structured, in-depth interviews using a topic guide informed by the UNFPA rights-based family planning framework and the ETR Health Equity Framework. The UNFPA framework informed questions relating to informed choice, reproductive autonomy, non-discrimination, respectful care, participation in decision-making, and quality of care. The ETR Health Equity Framework sensitized the interviews to structural, institutional, psychological, individual, relational, and social circumstances that could constrain or facilitate access.

The family planning component addressed seven areas: (1) sociodemographic, migration, and reproductive background; (2) family planning knowledge and previous and current contraceptive practices; (3) contraceptive preferences and decision-making; (4) access to services and healthcare navigation; (5) information provision, communication, and counseling; (6) perceived quality and continuity of care; and (7) social, cultural, relational, psychological, financial, and institutional influences on family planning experiences. The guide also captured participant characteristics that were subsequently used to contextualize descriptive cross-case comparisons. The complete interview guide and consent form are provided in Supplementary File 2.

The guide was developed by an interdisciplinary team with expertise in qualitative research, sexual and reproductive health, migration and health, public health, midwifery, and clinical care. Team review considered relevance to the research questions, clarity, cultural sensitivity, the wording of potentially sensitive questions, logical sequencing, adequacy of probes, and consistency with the two conceptual frameworks.

The original interview guide was developed and pilot-tested in English. Before multilingual data collection began, interviewers reviewed key concepts and reproductive health terminology to support conceptual consistency across languages.

Interviews were conducted in participants’ preferred study language: English (*n* = 21), Akan (*n* = 2), Arabic (*n* = 2), Bengali (*n* = 1), and Dari/Farsi (*n* = 3). The eight non-English interviews were conducted directly by trained multilingual research assistants who were fluent in the relevant languages. To preserve contextual and linguistic understanding, each non-English interview was translated into English by the same multilingual research assistant who had conducted the interview. The English-language material was subsequently used in the analytical process.

Interviews lasted approximately 60–90 min and were audio-recorded with participants’ permission. Interviewers also made brief field notes to document issues requiring follow-up and contextual observations relevant to interpretation. These notes supported the analytical process but were not treated as an independent dataset. Audio recordings were transcribed verbatim. Transcripts were reviewed against the corresponding audio recordings for accuracy and corrected where necessary before analysis. For interviews conducted in languages other than English, the multilingual research assistant who conducted the interview translated the transcript into English, with attention to contextual and culturally specific meanings. All transcripts and translated interview materials were pseudonymized before analysis.

### Pilot testing and interview-guide refinement

The interview guide was pilot-tested in English with two women with migration histories who met the study eligibility criteria. Each pilot interview was conducted by a different researcher, audio-recorded with the participant’s consent, and subsequently reviewed by the study team.

The pilot-testing process proceeded in four stages. First, each interviewer assessed participants’ comprehension of the questions and identified wording that required clarification or additional explanation. Second, the researchers reviewed the recordings and interview notes to assess sequencing, flow, question length, repetition, sensitivity, and the usefulness of planned probes. Third, the two pilot interviews were compared during a team debriefing, with particular attention to family planning terminology, transitions between sensitive topics, and whether the questions enabled participants to describe their experiences in their own terms. Fourth, the interview guide was revised before the start of the main data collection.

Revisions included simplifying selected wording, improving transitions between sections, and strengthening probes related to family planning decision-making, information provision, healthcare navigation, access barriers, and provider communication. No major restructuring of the guide was required.

The two pilot interviews were excluded from the final analytical dataset. Because both pilot interviews were conducted in English, the guide was not formally pilot-tested in Akan, Arabic, Bengali, or Dari/Farsi. This limitation is addressed in the Strengths and Limitations section.

### Data adequacy

Data collection and preliminary analysis proceeded iteratively. During data collection, the research team periodically discussed emerging codes, variation across participants, and areas requiring further exploration or probing in subsequent interviews.

After 25 main-study interviews, the team reviewed the developing analytical framework and judged that subsequent interviews were contributing limited new information relevant to the central research questions. Four additional interviews were conducted to examine whether the developing themes remained adequate across participants with differing migration pathways and reproductive health experiences and to strengthen variation within the sample.

After 29 main-study interviews, the team judged that the dataset provided sufficient thematic depth, variation, and contextual detail to address the study objectives. This judgment referred to the adequacy of the dataset for qualitative interpretation and theory development and did not imply statistical representativeness or population-level saturation. The two pilot interviews were not included in the final analysis.

### Data analysis

Analysis proceeded through five linked stages: thematic analysis, Health Equity Framework mapping, participant-level matrix construction, descriptive cross-case analysis, and integration and conceptual-model development. The qualitative analysis remained the methodological core throughout.

#### Stage 1: Thematic analysis

Interview transcripts were analyzed in NVivo 12 using inductive and deductive thematic coding. Deductive coding was sensitized by the UNFPA rights-based family planning framework and the ETR Health Equity Framework, while inductive coding allowed participants’ experiences and care needs to extend beyond the predefined frameworks. Analysis involved iterative transcript reading, coding, comparison within and across cases, theme development, analytic memo writing, and team discussion [41].

For interviews conducted in languages other than English, the English translations prepared by the multilingual research assistants who had conducted the respective interviews formed the material used for coding and analysis. Because the interviewer and translator were the same person, the translation process retained the interviewer’s direct familiarity with the interview context, participants’ intended meanings, and the circumstances in which statements were made. Interpretation nevertheless remained attentive to the possibility that linguistic and culturally embedded meanings may not have exact equivalents across languages.

The qualitative coding tree, operational definitions, and coding rules are provided in Supplementary File 3.

#### Stage 2: Health equity framework mapping

Themes and codes related to family planning and reproductive health access were mapped to the four ETR Health Equity Framework pathways: Systems of Power, Physiological Pathways, Individual Pathways, and Relationships and Networks. For the qualitative analysis, themes and codes were organized within these four pathways without further categorization as barriers or facilitators. The barrier–facilitator classification was applied subsequently, based on the qualitative interpretation of participants’ accounts, specifically to inform the quantitative component of the study. This qualitative classification guided the assessment and reporting of whether corresponding access barriers or facilitators were present in the quantitative data. Pathway definitions, the codebook, and the barrier–facilitator classifications used for this purpose are provided in Supplementary File 4.

#### Stage 3: Participant-level matrix construction and variable definition

Following the qualitative analysis, selected codes were organized in a participant-by-code matrix to support systematic cross-case comparison [38, 42]. The final matrix comprised 48 variables: 4 family-planning unmet-need indicators, 17 participant characteristics, and 27 pathway variables, including 15 barriers and 12 facilitators across Psychological, Individual, Systems of Power, and Relationships and Networks pathways. Binary variables used for family-planning unmet needs and pathway barriers and facilitators were coded as 1 when identified and 0 when not identified; ambiguous or insufficient information was coded as missing. Detailed definitions and coding rules are provided in Supplementary File 4.

Matrix entries were checked against coded excerpts, analytic memos, and, where necessary, the original transcripts to ensure consistency with the underlying qualitative material. Participant-level coded data are not provided because of data privacy considerations.

#### Stage 4: Descriptive cross-case analysis

The participant-level matrix was used descriptively to examine the distribution, overlap, coexistence, and heterogeneity of qualitative findings across the 29 participant accounts. This stage was intended to support systematic qualitative cross-case comparison and theory development rather than hypothesis testing, estimation of population-level relationships, or statistical inference.

Care needs, barriers, facilitators, and Health Equity Framework pathways were summarized using participant counts, percentages, and available-case denominators. The numbers of coded barriers and facilitators per participant account were also summarized using means to describe their distribution and coexistence within the sample.

Selected cross-tabulations were used to describe the co-occurrence of care needs and pathway conditions across participant accounts. For example, structural racism was cross-tabulated with respect and shared responsibility needs, and financial barriers with continuity and affordability needs. Because both sets of variables were derived from the same qualitative accounts and some constructs were conceptually related, these cross-tabulations were not treated as independent evidence of associations between constructs. They were reported using cell counts to describe patterns of overlap and were not interpreted as statistical tests, causal relationships, or confirmation of the qualitative findings.

Recurrent combinations of care needs, barriers, and facilitators were summarized using participant counts when they illustrated heterogeneity or the coexistence of supportive and constraining conditions. Participant characteristics were used to contextualize individual accounts and support cross-case comparison, but participants were not assigned to fixed profiles or types.

Patterns presented in the main text were selected according to their descriptive prominence, interpretability, relevance to the research questions, and ability to contextualize or extend the qualitative findings. Matrix-based patterns were returned to coded excerpts and, where necessary, the original participant accounts before interpretation to ensure that the descriptive summaries remained grounded in the qualitative material.

Supplementary exploratory analyses examined sample-specific relationships between participant characteristics and pathway variables using Spearman’s rank correlation for ordered or continuous characteristics and bias-corrected Cramér’s V for nominal characteristics. Given the small sample (*N* = 29), these coefficients were considered potentially unstable and were used only to identify patterns for hypothesis generation. They were not interpreted as confirmatory, predictive, causal, or generalizable estimates of population associations.

Supplementary File 5 presents care-need counts and percentages; barrier and facilitator counts and percentages; participant-level barrier and facilitator counts; descriptive cross-tabulations and co-occurrence analyses; Spearman’s rank correlations; bias-corrected Cramér’s V analyses; and accompanying notes to support cautious, descriptive interpretation of these exploratory findings.

#### Stage 5: Integration and conceptual-model development

Qualitative themes and descriptive cross-case findings were integrated through cross-case comparison, narrative weaving, and joint displays [43, 44]. Integration considered convergence, where matrix patterns supported the qualitative findings; complementarity, where descriptive patterns added information about distribution, coexistence, or heterogeneity; and divergence or ambiguity, where findings did not support a clear or consistent interpretation [43, 45]. Selected matrix patterns were compared with coded excerpts and original transcripts before integrated interpretations were developed. The joint display in Table 3 presents the qualitative findings, descriptive cross-case patterns, and integrated interpretations in the Results section.

The conceptual model was developed iteratively during integration. It synthesized the four interacting spheres of influence of the Health Equity Framework—Systems of Power, Relationships and Networks, Individual Pathways, and Psychological Pathways—with the four care-need domains identified in this study, contextual circumstances, supportive and constraining conditions, family planning access, and reproductive autonomy [40]. The framework’s Physiological Pathways sphere was adapted to Psychological Pathways, reflecting the predominance of psychological rather than biological mechanisms in the empirical data. The model is interpretive and hypothesis-generating; its relationships and arrows do not imply established causality, mediation, prediction, or population-level generalizability.

### Rigor and quality assurance

Interviews were conducted by B.H., N.J.A.R., and five trained multilingual research assistants with backgrounds in midwifery, medicine, international health, and qualitative research. Six members of the interviewing team were women and one was a man. The lead researchers had personal migration histories, while the wider team included researchers with and without migration histories. No interviewer or multilingual research assistant had a prior personal relationship with any participant before recruitment or data collection. Before participation, women were informed of the researchers’ institutional affiliation, professional and research roles, the purpose of the study, and the reasons for conducting the research.

Rigor was supported through interdisciplinary and multilingual team involvement, reflexive discussion, researcher comparison, and documentation of analytical decisions. Throughout data collection and analysis, the team considered interviewer–participant dynamics, positionality, emerging interpretations, coding decisions, and alternative explanations, consistent with reflexive approaches to qualitative research [46]. The team specifically considered how researchers’ gender, migration history, professional background, language, cultural experiences, and assumptions could influence rapport, participant disclosure, data interpretation, and the meanings attributed to participants’ accounts. These influences were examined through reflexive team discussions and deliberate consideration of alternative explanations during data collection and analysis. Two researchers analyzed transcripts in separate NVivo projects, with a third researcher reviewing and comparing the analytical outputs. Differences were resolved through discussion to refine coding definitions and interpretations rather than through calculation of intercoder reliability. Key analytical decisions were documented in shared memos to provide an audit trail and enhance transparency of the analytical process [47]. Preliminary findings were also discussed with two participants as an additional source of reflection rather than as a formal validation procedure [48].

Multilingual data collection and translation were incorporated into the quality-assurance process. Before data collection, multilingual interviewers reviewed key family planning and reproductive health concepts to support conceptual consistency across languages. Each of the eight interviews conducted in a language other than English was translated into English by the multilingual research assistant who had conducted that interview. This approach enabled the translator to draw on direct knowledge of the interview context, conversational flow, and participants’ intended meanings when rendering the material in English. The research team remained attentive during interpretation to the possibility that culturally specific expressions or meanings could be altered or reduced during translation. The absence of formal pilot testing of the interview guide in the four non-English language groups is considered separately as a methodological limitation.

For the participant-by-code matrix, variables were defined using a participant-level codebook and checked against coded excerpts, analytic memos, and, where necessary, original transcripts. Ambiguous or insufficiently supported cases were coded as missing, and no values were imputed. The team reviewed operational definitions, coding decisions, and selected cross-case patterns, with particular caution applied to small or unevenly distributed counts. The matrix supported systematic comparison across participants and analytical categories while retaining links to the underlying qualitative material [39].

During integration, matrix patterns were interpreted alongside the qualitative material and returned to participants’ accounts to assess convergence, complementarity, or divergence across findings [43]. Supportive and constraining conditions were allowed to coexist within the same account; therefore, facilitators were not interpreted as evidence that barriers had been resolved. These procedures supported systematic cross-case comparison while preserving the qualitative analysis as the methodological core.

Credibility was supported through iterative analysis, researcher comparison, reflexive discussion, return to the original transcripts and coded excerpts, and discussion of preliminary findings with two participants. Transferability was supported through detailed description of the study setting, participant characteristics, recruitment procedures, and contextual variation within the sample. Dependability was strengthened through documentation of coding procedures, operational definitions, analytical decisions, and shared memos that provided an audit trail. Confirmability was supported through reflexive analysis, comparison of interpretations across researchers, deliberate consideration of alternative explanations, and repeated checking of matrix-based findings against the underlying qualitative material. Authenticity was supported by preserving variation, contrasting experiences, and less common perspectives in participants’ accounts rather than seeking a single or uniform interpretation of family-planning access and care needs.

### Reporting standards

Reporting of the mixed-methods design was guided by the Good Reporting of A Mixed Methods Study (GRAMMS) recommendations [49]. Reporting addressed the rationale for using mixed methods, the priority and sequence of the qualitative and quantitative components, the transformation of qualitative codes into participant-level variables, the exploratory quantitative procedures, integration of findings across methods, and limitations arising from the mixed-methods design. The completed GRAMMS checklist is provided in Supplementary File 6.

Because qualitative interviews constituted the empirical core of the study, reporting of the qualitative component was guided primarily by the Consolidated Criteria for Reporting Qualitative Research (COREQ), a 32-item checklist for studies using interviews and focus groups [50]. The Comprehensive Criteria for Reporting Qualitative Research (CCQR) were additionally used to support comprehensive organization and reporting of the qualitative components of the manuscript [51]. Completed COREQ and CCQR checklists are provided in Supplementary File 7 as Supplementary Tables S1 and S2, respectively.

### Ethical considerations

Ethical approval was obtained from the Ethics Committee of Charité–Universitätsmedizin Berlin (EA4/076/23), and site-level permissions were obtained from the participating recruitment organizations. All participants provided written informed consent after receiving information about the study procedures, confidentiality safeguards, the voluntary nature of participation, and their right to decline to answer any question or withdraw from the study without adverse consequences. The study was conducted in accordance with the ethical principles set out in the Declaration of Helsinki [52].

Interviews were conducted by trained researchers in participants’ preferred study languages. Audio recordings were labeled using study identification codes and stored securely. Transcripts, English translations, analytical files, and participant-level matrix data were pseudonymized and accessible only to authorized members of the research team. Personal data were processed in accordance with the European Union (EU) General Data Protection Regulation (GDPR), Regulation (EU) 2016/679 [53].

## Results

### Participant characteristics

The study included 29 participants from 17 countries of origin. Their mean age was 33.8 years (standard deviation [SD] = 4.8; range, 25–45 years). Participants had lived in Germany for a mean of 5.5 years (SD = 3.9; range, 0–20 years) and in Berlin for a mean of 5.0 years (SD = 2.5; range, 0–9 years). Family reunification was the most common migration pathway (*n* = 13), followed by education (*n* = 7), employment (*n* = 5), and protection or asylum (*n* = 4). Most participants were married (*n* = 25), and 19 had tertiary-level qualifications. Additional sociodemographic and migration characteristics are presented in Table 1.

**Table 1:** Sociodemographic and migration characteristics of participants (*N* = 29)

Variable	Category	Frequency (*n*)
Age, years	25–29	3
	30–34	15
	35–39	8
	40–45	3
	Range; mean ± SD	25–45; 33.8 ± 4.8
Time in Germany, years	0–2	5
	3–5	11
	6–10	11
	11–20	2
	Range; mean ± SD	0–20; 5.5 ± 3.9
Time in Berlin, years	0–2	5
	3–5	11
	6–9	13
	Range; mean ± SD	0–9; 5.0 ± 2.5
Migration pathway	Family reunification	13
	Protection or asylum	4
	Education	7
	Employment	5
Marital status	Married	25
	Single	4
Education level	Secondary or high school	10
	Bachelor’s degree	7
	Master’s degree	11
	Doctoral degree	1
Country of origin	Pakistan	5
	Mexico	4
	Afghanistan	3
	Ghana	3
	Turkey	2
	Other countries*	12
Employment status	Employed	10
	On parental or maternity leave	7
	Not currently employed	12

Other countries included Argentina, Bangladesh, Guinea, Guinea-Bissau, India, Indonesia, Lebanon, the Philippines, Somalia, Vietnam, Yemen, and Zimbabwe

*Abbreviation: SD* Standard deviation

Participants had a mean of 1.3 children (SD = 0.8; range, 0–3) and 2.0 pregnancies (SD = 1.1; range, 1–4). At the time of interview, 20 were parenting a child younger than two years, eight were pregnant, and one had recently experienced an abortion.

Thirteen participants were not currently using a family-planning method. Among the 16 using contraception, six reported condoms or other barrier methods, five withdrawal or other natural or traditional methods, two hormonal methods, one a copper intrauterine device, and two the concurrent use of methods from more than one category.

Over their lifetimes, 15 participants had used methods from more than one contraceptive category, although not necessarily simultaneously. These combinations comprised barrier and hormonal methods (*n* = 7), barrier and natural or traditional methods (*n* = 4), barrier and hormonal methods together with a copper intrauterine device (*n* = 2), barrier, hormonal, and natural or traditional methods (*n* = 1), and hormonal and natural or traditional methods (*n* = 1). Additional reproductive and family planning characteristics are presented in Table 2.

**Table 2:** Reproductive history, current reproductive status, and family-planning methods of participants (*N* = 29)

Variable	Category	Frequency (*n*)
Number of children	0	3
	1	16
	2	7
	3	3
	Range; mean ± SD	0–3; 1.3 ± 0.8
Total pregnancies	1	12
	2	7
	3	7
	4	3
	Range; mean ± SD	1–4; 2.0 ± 1.1
Number of miscarriages	0	19
	1	8
	2	2
	Range; mean ± SD	0–2; 0.4 ± 0.6
Current reproductive status	Parenting a child younger than two years	20
	Currently pregnant	8
	Recently experienced an abortion	1
Current family-planning method	Not using a method	13
	Condoms or other barrier methods	6
	Withdrawal or other natural or traditional methods	5
	Pills or other hormonal contraceptives	2
	Copper intrauterine device	1
	Mixed or combined current methods	2
Lifetime family-planning experience	Never used a method	1
	Barrier methods only	7
	Natural or traditional methods only	3
	Hormonal methods only	3
	Intrauterine device only	0
	Methods from more than one category	15
Combinations among participants with mixed lifetime experience (*n* = 15)	Barrier and hormonal methods	7
	Barrier and natural or traditional methods	4
	Barrier and hormonal methods plus a copper intrauterine device	2
	Barrier, hormonal, and natural or traditional methods	1
	Hormonal and natural or traditional methods	1

Mixed or combined current methods refers to the concurrent use of methods from more than one contraceptive category at the time of interview. Mixed lifetime experience refers to the use of methods from more than one category over a participant’s lifetime; these methods were not necessarily used simultaneously. The lifetime combinations are mutually exclusive and include only the 15 participants classified as having mixed lifetime experience. *IUD* Intrauterine device, *SD* Standard deviation

### Family planning care needs

Participants’ family planning care needs were organized into four interrelated themes: communication and information, cultural and life-course responsiveness, continuity and affordability and respect and shared responsibility. The themes were not mutually exclusive, and participants could contribute to more than one theme. A theme coded as not identified in an interview was not interpreted as evidence that the corresponding care need was absent.

#### Communication and information

Participants described difficulties obtaining, locating, understanding, and translating family planning information. These difficulties concerned not only the amount of information provided but also its clarity, timing, and relevance to individual reproductive preferences. Several participants perceived consultations as time-constrained and reported receiving limited information unless they raised specific questions. Participants therefore often described initiating discussions themselves and seeking additional information independently.

Bushra explained that she prepared questions in advance and conducted her own research before appointments:

"Here [in Germany], doctors don’t usually give you a lot of information. You kind of have to push them and ask questions yourself. I always had my questions ready, so I kept asking them and doing my own research." *(Bushra*,* 33 years*,* Pakistan)*

When formal healthcare sources did not fully meet their informational needs, participants described using alternative sources, including websites, artificial intelligence tools, translation applications, and menstrual-cycle tracking applications. Kylie described artificial intelligence tools as an important part of how she currently accessed and managed health-related information:

"ChatGPT has changed everything… That is how I am surviving now." *(Kylie*,* 39 years*,* Zimbabwe)*

Together, these accounts illustrate how participants used self-directed strategies to locate, interpret, translate, and apply family planning information when formal information pathways were perceived as insufficient or difficult to access.

#### Continuity and affordability

Participants emphasized the importance of being able to maintain a preferred contraceptive method without substantial personal cost, repeated appointments, administrative requirements, or practical burden. Financial constraints were particularly salient among participants with limited financial resources.

Rosa described obtaining contraceptive supplies through personal contacts outside Germany because of cost:

"I had to pay for them… and because I’m not working it can be expensive. Sometimes I ask people to bring them from Portugal." *(Rosa*,* 38 years*,* Guinea-Bissau)*

Participants also described administrative requirements that complicated continued access to an established method. Isabella explained that obtaining another prescription after six months required an additional appointment despite no change in her contraceptive preference:

"My doctor only gave me a prescription for six months, so I have to go back again to get a new prescription. It feels like a lot of wasted time." *(Isabella*,* 33 years*,* Mexico)*

Across these accounts, continuity involved not only obtaining an initial contraceptive method but also being able to sustain preferred care without repeated financial, administrative, caregiving, or other practical burdens.

#### Respect and shared responsibility

Participants described two related dimensions of respectful family planning care: the distribution of contraceptive responsibility between women and men and respect for women’s preferences and decisions within clinical encounters.

Several participants questioned the concentration of contraceptive responsibility and associated side effects among women. Hina described the limited range of contraceptive options available to men:

"Most contraception methods are more focused on women than on men. The side effects are also mostly on women—hormonal side effects or birth-control side effects. For men, there are very few methods." *(Hina*,* 30 years*,* Pakistan)*

Participants also emphasized the importance of respecting decisions to accept or decline a contraceptive method. Sana described repeatedly being encouraged to use an intrauterine device despite preferring condoms:

"My doctor has been trying to tell me to get a coil. I told her that I would get back to her, but I do not plan to do it. I am fine with condoms for now, and I do not want to put anything else in my body." *(Sana*,* 37 years*,* Pakistan)*

Ifrah similarly described respectful care as requiring individualized dialogue rather than standardized assumptions:

"You should not put me in your box. You also need to listen to what works for me so that we can meet somewhere in the middle." *(Ifrah*,* 32 years*,* Somalia)*

Across these accounts, participants emphasized reproductive autonomy, respect for informed refusal, collaborative decision-making, and a more equitable distribution of responsibility for family planning.

#### Cultural and life-course responsiveness

Participants described a need for family planning care that accounted for previous healthcare experiences, migration-related and cultural context, and changing reproductive circumstances without relying on generalized assumptions about individuals or groups.

Comparisons with healthcare received in participants’ countries of origin sometimes shaped expectations of care in Germany. Linh described receiving proactive contraceptive counseling after childbirth in her country of origin, whereas in Germany she perceived that the topic was discussed only when she raised it herself:

"In my country they give birth control information right after the baby is born, but here nobody talked to me about it unless I asked." *(Linh*,* 30 years*,* Vietnam)*

Participants also described family planning needs as changing across reproductive stages. Roya, who was breastfeeding at the time of interview, explained:

"Right now, I am breastfeeding and using condoms, but I feel like I should probably talk to a doctor about other options that might work better for me later." *(Roya*,* 25 years*,* Afghanistan)*

These accounts illustrate how contraceptive preferences and counseling needs could evolve across reproductive stages and be shaped by previous healthcare experiences, current reproductive circumstances, and migration-related context.

### Pathways shaping access

Whereas the preceding themes describe what participants identified as necessary for appropriate family planning care, the Education, Training, and Research (ETR) Health Equity Framework pathways describe the conditions through which access to such care was constrained or supported.

Four interrelated pathways were identified: Systems of Power, Psychological Pathways, Individual Pathways, and Relationships and Networks. These pathways shaped participants’ ability to obtain, understand, afford, navigate, and continue care aligned with their preferences and circumstances.

#### Systems of power

Participants described racism and discrimination, uncertainty about healthcare entitlements, financial constraints, and housing or material insecurity as structural conditions shaping access to reproductive healthcare. These experiences affected not only access to services but also participants’ trust, confidence, and sense of safety within healthcare encounters.

Elina described an incident during pregnancy in which she perceived unequal treatment at a gynecological clinic:

"I arrived early, even before my scheduled time. Every time someone came in after me, they were attended to immediately. I sat there for almost two hours waiting. I told her directly, ‘This is not right; it’s racism.’" *(Elina*,* 25 years*,* Guinea)*

Bushra recalled racial othering following a medication-related complication:

"The doctor told me, ‘Sorry, because you’re my first brown patient, I didn’t consider that this could be a problem.’" *(Bushra*,* 33 years*,* Pakistan)*

Ifrah described racism and discrimination as extending beyond isolated interpersonal encounters:

"The negative here [in Germany] is racism and discrimination. This is structural. When you are Black, people assume things about you." *(Ifrah*,* 32 years*,* Somalia)*

Legal and administrative uncertainty also complicated healthcare access. Roya recalled:

"I was in the Heim [refugee accommodation]. I did not know which services I was actually entitled to." *(Roya*,* 25 years*,* Afghanistan)*

Across these accounts, structural and institutional conditions shaped both access to reproductive healthcare and participants’ experiences within the healthcare system.

#### Psychological pathways

Participants described psychological experiences related to war, displacement, female genital cutting, reproductive loss, and distressing healthcare encounters. In some accounts, these experiences were explicitly linked to fear or uncertainty regarding reproductive health. In others, participants were unsure whether or how these experiences influenced reproductive decisions or healthcare engagement.

Ifrah linked her childhood experience of female genital cutting to anxiety about childbirth:

"I was cut when I was six years old. Sometimes I wonder if I will even be able to give birth because of the cutting. That is one of my biggest fears." *(Ifrah*,* 32 years*,* Somalia)*

Khatoon described traumatic memories associated with conflict and displacement:

"The night the Taliban attacked, I saw my brother being killed in front of my eyes. I have these memories in my head so many times." *(Khatoon*,* 35 years*,* Afghanistan)*

When asked whether these experiences affected her health or reproductive decisions and whether they had been addressed by a healthcare professional, she added:

"I cannot say if it is relevant to it. I don’t know. No one ever asked me, and honestly, I prefer not to share because it brings me pain." *(Khatoon*,* 35 years*,* Afghanistan)*

These accounts indicate that psychological distress formed part of some participants’ broader reproductive health context, although participants did not always identify a direct relationship between traumatic experiences and family planning decisions or healthcare engagement.

#### Individual pathways

Participants varied in health literacy, German-language proficiency, familiarity with the healthcare system, exposure to family planning information, and confidence in navigating healthcare and contraceptive decisions. These resources shaped how participants understood available options, sought information, communicated with providers, and navigated services.

Lola described language-related difficulty completing routine healthcare tasks:

"My experience as a migrant in Germany has been quite difficult because of the language. It was hard even to go to the hospital or book an appointment online, so language was a major challenge for me." *(Lola*,* 35 years*,* Ghana)*

Self-advocacy also emerged as an important resource. Jasmine explained:

"You need to be assertive and ask questions to make sure you are getting the right treatment. If you think you need more examinations or further treatment, you need to ask for it, seek a second opinion, or go to another doctor." *(Jasmine*,* 37 years*,* Philippines)*

Participants’ accounts suggested that the capacity for self-advocacy was associated with familiarity with the German healthcare system, language skills, previous healthcare experiences, professional knowledge, and awareness of healthcare rights. These resources could facilitate access to care but were not equally available across participants.

#### Relationships and networks

Relationships and social networks operated as both sources of constraint and sources of support. Social isolation, childcare responsibilities, limited practical support, stigma, and discouraging information could complicate access, whereas peer, community, and digital networks could facilitate information exchange, navigation, and emotional support.

Sana described how concerns circulating within her social network contributed to her reluctance to use hormonal contraception:

"I’ve heard so many horror stories about how it messes up your hormones. I just never ended up taking it." *(Sana*,* 37 years*,* Pakistan)*

Anicka described establishing an online support group through which women could exchange experiences and information:

"I started an online support group where women could share their experiences and information with each other, because knowledge is power." *(Anicka*,* 45 years*,* Argentina)*

For some participants, limited personal support, childcare responsibilities, and social isolation complicated appointment attendance and healthcare navigation. For others, community, peer, and digital networks facilitated access to information and services. Relationships and Networks therefore operated in different ways depending on the nature and quality of the support available.

### Exploratory matrix findings

The participant-by-code matrix supported descriptive cross-case comparison of care needs, barriers, facilitators, and ETR Health Equity Framework pathways across the 29 participant accounts. Consistent with the qualitatively driven design, these findings are exploratory and sample-specific and were interpreted alongside the qualitative analysis.

#### Distribution and coexistence

Communication and information needs were identified in 28 of 29 participants, cultural and life-course responsiveness needs in 26, continuity and affordability needs in 24, and respect and shared responsibility needs in 22. These needs commonly overlapped within participant accounts.

The most common barriers were mental-health concerns (23 participants), childcare burden/stigma [22], and social isolation [19]. The most common facilitators were provider compassion/empathy (28 participants), self-advocacy [25], and decision-making confidence and healthcare-navigation support (24 each). All 29 accounts contained at least one barrier and one facilitator, with a mean of 7.03 of 15 barriers and 7.28 of 12 facilitators identified per account.

**Fig. 2:**
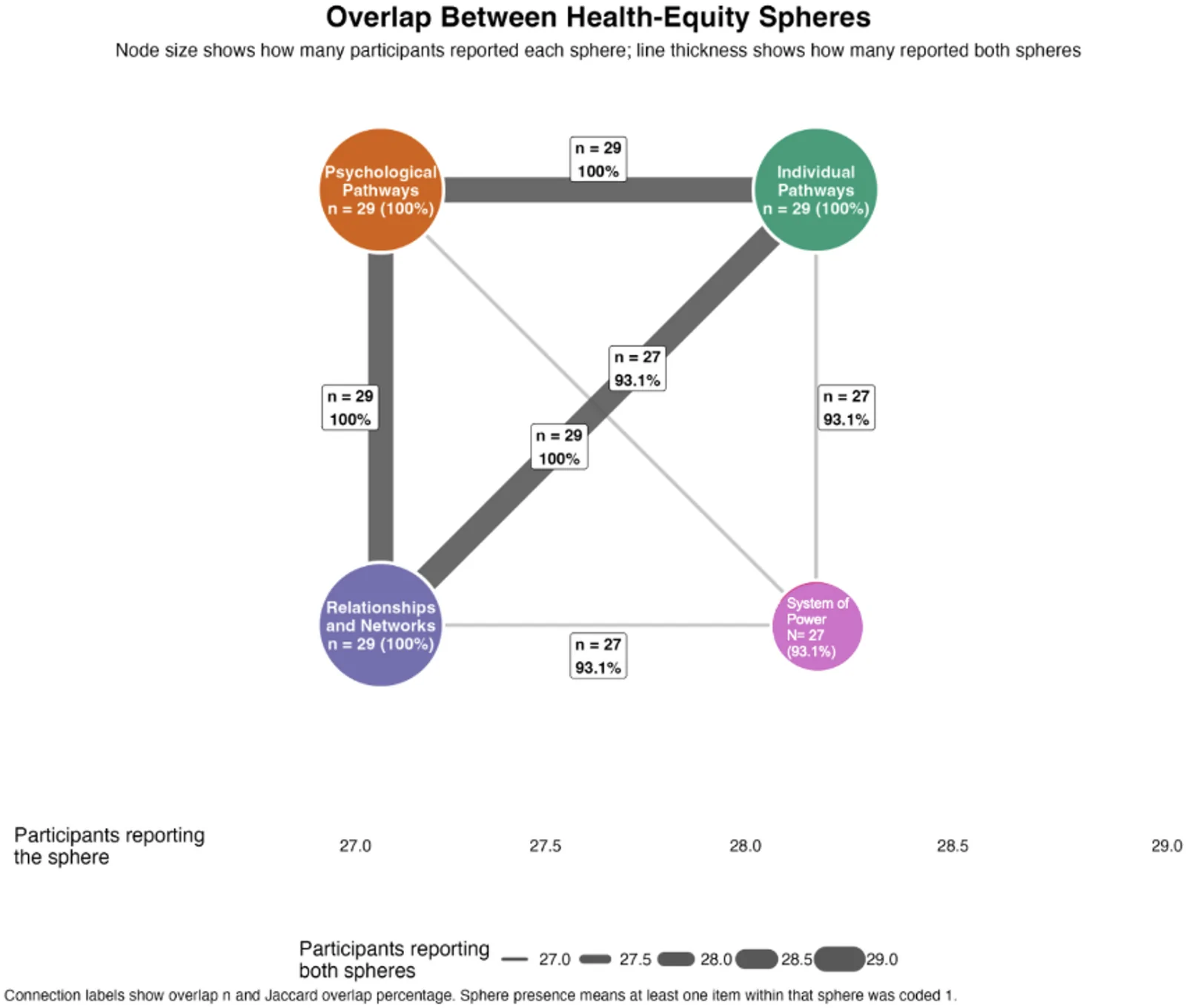
Overlap among Health Equity Framework pathways shaping access to family planning and reproductive healthcare. Node size represents the number of participants in whose accounts each pathway was identified, and line width represents the number of participants in whose accounts both connected pathways were identified

Psychological Pathways, Individual Pathways, and Relationships and Networks were represented in all 29 accounts, while Systems of Power was represented in 27. Pathways frequently overlapped within individual accounts (Fig. 2).

#### Cross-case patterns

Descriptive cross-case comparisons showed that psychological distress and childcare burden were identified across multiple care-need domains rather than being confined to a single type of need. Provider compassion and self-advocacy also remained common among participants with unresolved care needs, illustrating that supportive resources could coexist with ongoing constraints.

Structural racism co-occurred with a respect and shared responsibility need in 16 of 22 accounts in which that need was identified and in none of the seven accounts without it. Financial barriers co-occurred with a continuity and affordability need in 12 of 24 accounts in which that need was identified and in none of the five accounts without it. Because these variables were derived from the same qualitative accounts and were conceptually related, the cross-tabulations were interpreted only as descriptive patterns of code co-occurrence and not as independent evidence of associations, between-group differences, or causal relationships.

Co-occurrence analyses further illustrated the simultaneous presence of needs, constraining conditions, and supportive resources. Mental-health concerns, social isolation, and childcare burden frequently appeared within the same participant accounts. Limited German-language proficiency, a communication and information need, and healthcare-navigation support co-occurred in 15 accounts. A broader pattern of multiple unmet needs together with barriers and facilitators spanning at least three Health Equity Framework pathways each was identified in 21 participants, further illustrating the multidimensional and heterogeneous nature of family-planning access within the sample.

#### Exploratory associations with participant characteristics

Participants with similar migration-related, reproductive, or sociodemographic characteristics did not necessarily report similar care experiences. Within this heterogeneity, several sample-specific rank-order patterns were observed. Greater numbers of pregnancies corresponded with greater identification of financial barriers (ρ = 0.564), greater financial security corresponded with decision-making confidence (ρ = 0.554), and greater numbers of children corresponded with social isolation (ρ = 0.499). Total barrier and facilitator counts showed only a weak relationship (ρ = 0.159), consistent with the qualitative finding that supportive resources and constraining conditions could coexist.

Given the small sample size (*N* = 29) and the exploratory nature of these analyses, the coefficients were treated as potentially unstable and are presented for hypothesis generation rather than as estimates of population-level relationships. They should not be interpreted as causal, predictive, or confirmatory associations. Full Spearman correlations, bias-corrected Cramér’s V analyses, cross-tabulations, and co-occurrence results are presented in Supplementary File 5.

### Integration of qualitative and descriptive cross-case findings

The qualitative themes and descriptive matrix findings were integrated to assess convergence, complementarity, and heterogeneity across participant accounts (Table 3). The joint display illustrates how the matrix findings described the distribution, overlap, and coexistence of patterns identified in the qualitative analysis and provided additional cross-case context. These descriptive findings were interpreted alongside the underlying qualitative material rather than as independent statistical confirmation of relationships among constructs.

**Table 3:** Joint display of qualitative and descriptive cross-case findings

Integrated domain	Descriptive matrix finding	Qualitative evidence	Integrated interpretation
Communication, information, and navigation	Communication and information needs were identified in 28 of 29 accounts. Limited German-language proficiency, a communication and information need, and navigation support co-occurred in 15 accounts.	Participants described difficulties obtaining and understanding information, communicating with providers, and navigating services. They also relied on self-directed information seeking, digital tools, healthcare navigators, peers, and community networks.	Informational and navigation resources often helped participants respond to communication constraints but did not necessarily resolve the underlying care need.
Respect, autonomy, and structural conditions	Respect and shared responsibility needs were identified in 22 accounts. Structural racism was identified in 16 of these 22 accounts and in none of the seven accounts without this need.	Participants described racism, stereotyping, insufficient involvement in decision-making, pressure regarding contraceptive choices, and lack of respect for their preferences or informed refusal.	Experiences of disrespect extended beyond individual clinical encounters and were embedded in broader structural and relational conditions.
Continuity, affordability, and agency	Continuity and affordability needs were identified in 24 accounts. Financial barriers were identified in 12 of these 24 accounts and in none of the five accounts without this need.	Participants described financial costs, repeated appointments, administrative requirements, childcare responsibilities, and other practical burdens, while also using self-advocacy and information seeking to maintain access.	Agency often functioned as a means of navigating unresolved constraints rather than as evidence that those constraints had been removed.
Cultural and life-course responsiveness	Cultural and life-course responsiveness needs were identified in 26 accounts.	Participants described care needs shaped by previous healthcare experiences, migration context, cultural background, and changing reproductive circumstances.	Responsive care required attention to individual histories and reproductive stages without relying on generalized assumptions about cultural or migration backgrounds.
Coexistence across pathways	All 29 accounts included at least one barrier and one facilitator. Participants had a mean of 7.03 barriers and 7.28 facilitators, and Health Equity Framework pathways frequently overlapped.	Participants simultaneously described structural constraints, psychological distress, individual agency, and relational or provider support.	Barriers and supportive resources were not opposing states; substantial support and agency could coexist with unresolved constraints and care needs.
Participant characteristics and heterogeneity	Selected participant characteristics were associated with particular barriers or resources, but similar characteristics did not consistently correspond to similar overall care experiences.	Participants described different experiences despite apparently similar linguistic, financial, reproductive, or migration-related circumstances.	Participant characteristics shaped the context in which care was experienced but did not determine access. Their relevance depended on how they intersected with structural, psychological, individual, and relational conditions.

Cross-case findings are descriptive and specific to this sample. Exploratory associations with participant characteristics were interpreted as contextual signals rather than explanatory or predictive relationships. Integrated interpretations were developed by comparing descriptive matrix patterns with qualitative themes and participant accounts and should not be interpreted as evidence of causality or population-level relationships

#### Integrated interpretation

Taken together, the qualitative and descriptive cross-case findings indicate that family planning access was shaped by interacting care needs, barriers, and supportive resources rather than by isolated determinants. The matrix analysis confirmed that the principal qualitative themes were widely represented across participant accounts and showed that they frequently overlapped.

A central finding was that facilitators did not simply offset barriers. Participants could receive compassionate care, demonstrate self-advocacy, or access navigation support while still reporting unresolved communication, affordability, continuity, or respect-related needs. Supportive resources therefore often enabled participants to navigate or compensate for constraints without necessarily resolving them.

The integration also highlighted the importance of structural and material conditions. Structural racism was concentrated among participants with respect and shared responsibility needs, while financial barriers were concentrated among those with continuity and affordability needs. These patterns complemented qualitative accounts of discrimination, constrained autonomy, financial burden, and difficulties maintaining preferred care.

Selected associations with reproductive, socioeconomic, and migration-related characteristics provided additional context for the observed heterogeneity but did not support deterministic or stable participant profiles. Participants with similar characteristics could experience care differently depending on their resources, relationships, previous experiences, and encounters with the healthcare system.

Overall, the integrated findings support a multidimensional understanding of family planning access in which structural, psychological, individual, and relational conditions operate simultaneously and interact with participants’ circumstances, available resources, and healthcare responses. The central pattern was the coexistence of unmet needs, accumulated barriers, agency, and supportive resources within the same participant accounts, together with substantial heterogeneity in how these conditions were experienced.

### Integrated conceptual model

**Fig. 3:**
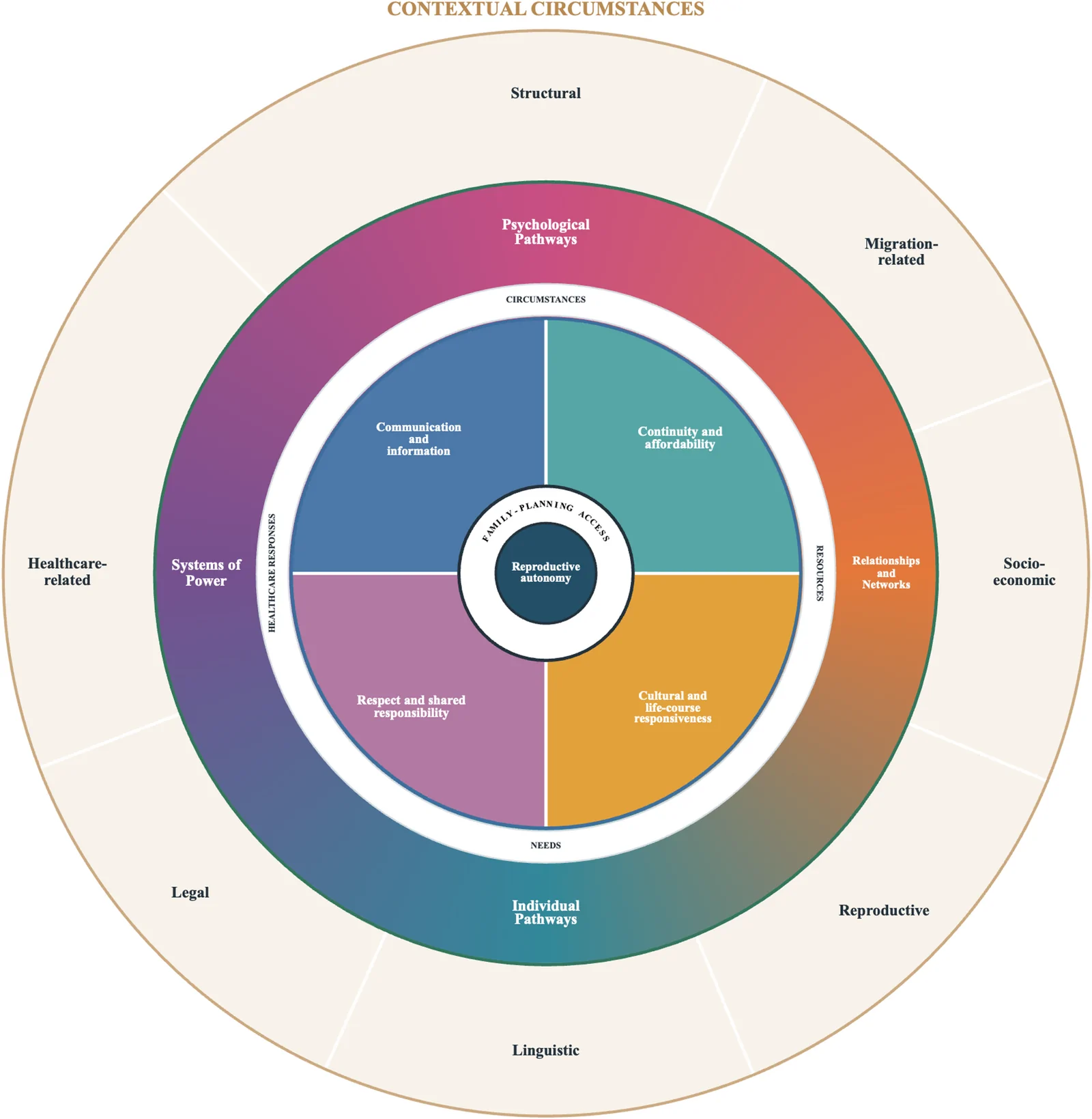
Integrated ecological–alignment model of family planning access and reproductive autonomy among women with migration histories in Berlin, Germany. Contextual circumstances—including structural, migration-related, socioeconomic, reproductive, linguistic, legal, and healthcare factors—surround and shape four interrelated ETR Health Equity Framework pathways: Systems of Power, Psychological Pathways, Individual Pathways, and Relationships and Networks. Supportive and constraining conditions may coexist within and across these pathways and influence how family-planning care needs related to communication and information, continuity and affordability, respect and shared responsibility, and cultural and life-course responsiveness are addressed. Family planning access and reproductive autonomy are conceptualized as emerging from the degree of alignment or misalignment between participants’ circumstances, available resources, care needs, and healthcare responses. Connectors indicate interpretive interrelationships rather than causal direction, mediation, prediction, or population-level generalizability

The integrated ecological–alignment model synthesizes the qualitative themes and descriptive cross-case findings (Fig. 3). It conceptualizes family planning access and reproductive autonomy as emerging from the degree of alignment between participants’ circumstances, available resources, care needs, and healthcare responses.

Overall, the model shows that Family planning access and reproductive autonomy emerge from the alignment—or misalignment—between women’s circumstances and care needs, the barriers and resources operating across Health Equity Framework pathways, and the healthcare responses available to them.

Structural, migration-related, socioeconomic, reproductive, linguistic, legal, and healthcare circumstances form the broader context within which the four interrelated ETR Health Equity Framework pathways operate: Systems of Power, Psychological Pathways, Individual Pathways, and Relationships and Networks. Supportive and constraining conditions may coexist within and across these pathways and shape whether and how family-planning care needs are addressed. These needs include communication and information, continuity and affordability, respect and shared responsibility, and cultural and life-course responsiveness.

Greater alignment between participants’ circumstances, available resources, care needs, and healthcare responses was associated with more supportive conditions for accessing care and exercising reproductive autonomy. By contrast, misalignment was reflected in greater informational, practical, financial, administrative, and emotional work required to obtain care consistent with participants’ preferences and circumstances. The model therefore emphasizes interaction and coexistence rather than a linear sequence of barriers and facilitators.

The model is interpretive and hypothesis-generating and should not be understood as causal, predictive, or representative of population-level relationships.

## Discussion

This study suggests that the availability of family-planning services does not necessarily ensure meaningful access or reproductive autonomy for women with migration histories. Participants could use services while still facing multiple barriers. Meaningful access therefore depended not only on service availability, but also on whether care was understandable, affordable, continuous, respectful, and responsive to participants’ circumstances, care needs, and reproductive preferences.

Reproductive autonomy emerged as a central dimension of access because being able to make an informed decision did not guarantee that participants could act on it. Defined as “the power to decide about and control matters associated with contraceptive use, pregnancy, and childbearing” reproductive autonomy may be constrained when women can form reproductive preferences but cannot readily translate those preferences into feasible and acceptable choices within the healthcare system [1, 3, 54, 55]. From a rights-based perspective, autonomy therefore depends on a healthcare system that supports not only decision-making but also the ability to carry out those decisions in accordance with one’s own preferences [56]. Access, in this sense, is achieved only when it moves beyond mere availability of services to include genuine alignment with, and responsiveness to, individuals’ needs and preferences. This is consistent with with findings from a study of family-planning services among refugee women in Berlin, where unmet family-planning and educational needs persisted despite the availability of complementary services [11].

Barriers and facilitators to access frequently coexisted. Facilitators helped participants navigate care without necessarily resolving the barriers they faced, a pattern also reported in studies of women seeking asylum and other populations with migration histories [57]. Self-advocacy was a clear example: participants sought information, used digital and translation tools, obtained second opinions, and drew on peer or community support, consistent with previous evidence on the role of informal networks, trusted information sources, and navigation strategies [54, 58, 59]. However, these strategies often emerged in response to gaps in service provision and could not fully compensate for underlying barriers [54, 57, 60]. Self-advocacy should therefore be understood as both a facilitator and an adaptive response to difficult-to-navigate healthcare systems, rather than as a substitute for accessible and responsive care.

Migration history was relevant not as a barrier in itself, but because migration-related circumstances could intensify existing barriers. In Germany, Kikhia et al. (2021) similarly identified limited system knowledge, uncertainty regarding health-insurance entitlements, and language barriers, while family support facilitated access [61]; Nowak and Hornberg (2023) reported language-related difficulties, disorientation within the healthcare system, and experiences of inappropriate treatment [62]; and Klein and von dem Knesebeck (2018) highlighted communication and information gaps and limited system knowledge [63]. Financial and administrative barriers may also persist despite formal coverage, as shown by differences in healthcare use according to administrative access arrangements and continued difficulties related to insurance coverage and access to medicines [61, 64, 65]. This is relevant in Berlin, where prescribed contraception is generally covered by statutory health insurance until the 22nd birthday and additional means-tested support is available to eligible residents thereafter [66, 67]. In this sense, the present findings reinforce earlier work showing that access depends not only on the existence of healthcare and financial-support mechanisms, but also on individuals’ ability to understand, navigate, and effectively use them [68–70].

Notably, the care needs identified in this study are not necessarily specific to women with migration histories [71, 72] and without a comparison group, between-group differences cannot be established. German evidence supports this caution, as Seidel et al. (2020) found low income to be a stronger predictor of delayed antenatal care than migration status [73, 74]. Communication, respect, and life-course responsiveness were closely connected in participants’ accounts. Communication needs extended beyond language, and Origlia Ikhilor et al. (2019) similarly showed that translation alone is insufficient without responsive provider communication and supportive organizational conditions [75]. Experiences of racism and stereotyping also reflected broader power relations within healthcare [76–78]. Provider perspectives were not examined, although counselling may be shaped by provider and organizational factors, and existing German guidance including the AWMF S2k guideline on non-hormonal contraception does not establish how consistently it is implemented in practice [72, 79, 80]. Finally, participants’ preferences changed across reproductive and life-course circumstances, reinforcing the need for individualized and responsive care rather than assumptions based on migration or cultural background [81].

Importantly, heterogeneity among participants also has implications for equity, as women with similar migration-related, reproductive, linguistic, or sociodemographic characteristics did not necessarily report similar experiences. This suggests that broader determinants interact with individual circumstances, resources, relationships, and healthcare responses. Fair et al. (2020) similarly cautioned against assumptions based on perceived cultural background [82]. Migration pathway, nationality, education, language proficiency, and reproductive history should therefore be treated as contextual factors rather than proxies for individual family-planning needs. Equitable access extends beyond service availability to affordable, continuous, respectful, non-discriminatory, and responsive care that supports informed choice and autonomy [71, 72, 83].

As part of interpreting these findings cautiously, alternative explanations were also considered. Some reported difficulties may reflect broader features of family-planning care or the German healthcare system rather than migration-related circumstances specifically. Time-constrained consultations, costs, prescription requirements, variation in provider communication, and limited continuity may affect women irrespective of migration history. Comparative evidence from Berlin supports this interpretation: Seidel et al. (2020) found that although some differences existed between immigrant and non-immigrant women, low income was a stronger predictor of delayed antenatal care than migration status [74]. Reproductive stage, socioeconomic circumstances, previous healthcare experiences, relationship circumstances, and personal preferences may likewise shape family-planning needs independently of migration-related factors [81, 83]. Given the absence of a comparison group, the findings should therefore be interpreted as showing how these conditions were experienced within migration-related contexts, rather than as demonstrating that migration history itself caused the observed care needs or barriers.

### Strengths and limitations

This study has several strengths. The qualitatively driven mixed-methods design enabled in-depth exploration of participants’ experiences together with systematic descriptive cross-case comparison. The sample included women from 17 countries with diverse migration pathways and reproductive experiences, while multilingual interviewing broadened participation beyond English-speaking participants. Additional strengths included an interdisciplinary and multilingual research team, reflexive and iterative analysis, transparent documentation of analytical decisions, discussion of preliminary findings with participants, and integration of qualitative and descriptive cross-case findings.

Several limitations should be considered. Recruitment may have underrepresented women with precarious legal or administrative circumstances, limited social networks, lower German-language proficiency or literacy, or less accessible living arrangements, while the sample was predominantly highly educated, married, and parenting young children. Eligibility criteria requiring recent maternal or reproductive healthcare contact and a pregnancy or childbirth within the preceding five years further limited transferability. Multilingual interviewing and translation may have introduced some loss of nuance, and the interview guide was formally pilot-tested only in English. The small sample limited systematic subgroup comparisons, so matrix-based analyses were used descriptively and for hypothesis generation rather than statistical inference. Researcher and participant subjectivities may also have influenced interpretation despite reflexive and collaborative procedures. Finally, several intersecting characteristics, including religion, sexual orientation, disability, social class, partner nationality, and racialization, were not fully captured. The findings are therefore context-specific and should be interpreted in terms of transferability rather than statistical generalizability.

### Practice implications

Family-planning services should strengthen rights-based, culturally responsive, trauma-informed, and anti-discriminatory care through active listening, shared decision-making, respect for informed refusal, proactive but non-coercive initiation of family-planning discussions, and avoidance of assumptions about women’s preferences, fertility intentions, or cultural backgrounds. Providers should consider how migration-related, legal and administrative, linguistic, socioeconomic, reproductive, and previous healthcare circumstances may shape individual care needs while avoiding the use of these characteristics as proxies for individual preferences.

Services should strengthen communication and healthcare navigation through clear, understandable information on contraceptive options, costs, eligibility, prescriptions, and follow-up care, supported by professional interpretation and translated materials where needed. Continuity should also be supported by reducing avoidable financial, administrative, and practical burdens associated with maintaining preferred methods. Community, peer, and digital resources may strengthen trust, information access, and navigation but should complement rather than replace formal healthcare provision. Partner involvement may support shared responsibility where desired by women while preserving reproductive autonomy, confidentiality, and informed choice.

### Research implications

Future research should examine family-planning access in larger and more diverse populations and further evaluate the integrated ecological–alignment model developed in this study. The Health Equity Framework pathways, four care-need domains, and participant-level variables may support hypothesis generation, measure development, and subsequent quantitative testing but require further conceptual and empirical validation before use as standardized measures.

Longitudinal, comparative, and participatory research could examine how family-planning care needs and access change across migration trajectories and reproductive life stages. Comparative designs including women without migration histories could help distinguish broadly experienced healthcare constraints from those that are intensified or configured differently within migration-related contexts. Future studies should also incorporate provider and health-system perspectives to examine how clinicians initiate family-planning discussions, whether concerns about cultural sensitivity or assumptions regarding patient preferences influence counselling, and how professional guidance is interpreted and implemented in routine practice. Such research could help distinguish individual provider-level factors from organizational and system-level conditions and clarify how these contribute to alignment or misalignment between women’s circumstances and care needs and available healthcare responses.

### Policy implications

Policy should move beyond formal service availability toward equitable and responsive family-planning access. Priorities include reducing financial and administrative barriers; strengthening multilingual information, professional interpretation, and healthcare-navigation support; and embedding rights-based, culturally responsive, trauma-informed, and anti-discriminatory approaches within routine care.

In the German context, this includes ensuring clear and accessible information on statutory insurance coverage, age-related contraceptive costs, eligibility requirements, and available financial-support mechanisms, including Berlin’s means-tested contraceptive cost-coverage scheme. Formal financial support and professional guidance should be accompanied by accessible counselling and navigation processes that support informed contraceptive choice and continuity of preferred care.

Health systems should monitor access using relevant social determinants of health (SDOH) indicators alongside context-specific equity measures, including affordability, language access, continuity of care, respectful treatment, informed choice, and disparities across social, legal, linguistic, and economic circumstances. Community and peer resources should complement, rather than compensate for, gaps in formal services. Responsibility for creating the conditions for equitable family-planning access and reproductive autonomy should remain primarily with healthcare systems and the broader structural and policy environments in which care is delivered.

## Conclusion

This study advances understanding of family-planning access by showing that meaningful access depends on the alignment between women’s circumstances, preferences, resources, and healthcare responses, rather than on service availability or individual knowledge alone. Barriers and facilitators frequently coexisted: participants could be informed, proactive, and socially supported while still encountering financial, linguistic, administrative, or discriminatory barriers. Individual agency may therefore support healthcare navigation, but it cannot be expected to compensate for structural and service-level constraints.

The conceptual model positions reproductive autonomy as relational and dependent on healthcare-system responsiveness, shaped by the interaction between contextual circumstances and the four Health Equity Framework pathways. Formal availability alone is insufficient when care is not aligned with women’s needs and preferences. Equitable family-planning access therefore requires health systems to reduce structural and practical barriers while providing understandable, affordable, continuous, respectful, and responsive care that enables informed choice and supports women in acting on their reproductive preferences.

## Supplementary Information

Supplementary Material 1.

## Data Availability

The datasets generated and/or analyzed during the current study are not publicly available due to ethical restrictions and th+e sensitive nature of the qualitative interview data but are available from the corresponding author on reasonable request, subject to ethical approval and data protection regulations.

## Abbreviations

AfEBB: African Parents Association Berlin-Brandenburg
CCQR: Comprehensive Criteria for Reporting Qualitative Research
COREQ: Consolidated Criteria for Reporting Qualitative Research
ETR: Education, Training, and Research
EU: European Union
GDPR: General Data Protection Regulation
GRAMMS: Good Reporting of A Mixed Methods Study
LMIC: Low- and middle-income country
LMICs: Low- and middle-income countries
QUAL: Dominant qualitative component in mixed-methods notation
Quan: Subordinate quantitative component in mixed-methods notation
SD: Standard deviation
SDOH: Social determinants of health
UNFPA: United Nations Population Fund
