# Supplementary material for: “Do not leave me behind and do not put me in your box”: family-planning access and care needs among women with migration histories in Berlin, Germany—a mixed-methods study
Source: Reprod Health. 2026 Sep 25;23:175. doi: 10.1186/s12978-026-02450-6 (PMC13613642; doi:10.1186/s12978-026-02450-6)
Supplement: Supplementary file 1 [file 12978_2026_2450_MOESM1_ESM.zip › Supplementary File 1_Recruitment Materials.pdf]

Supplementary File 1. Examples of Recruitment Materials

This supplementary file presents two illustrative examples of the multilingual recruitment materials used in the study: an English-language flyer and a Dari-language flyer. Recruitment flyers were available in all study languages: English, German, Akan, Arabic, Bengali, and Dari/Farsi.

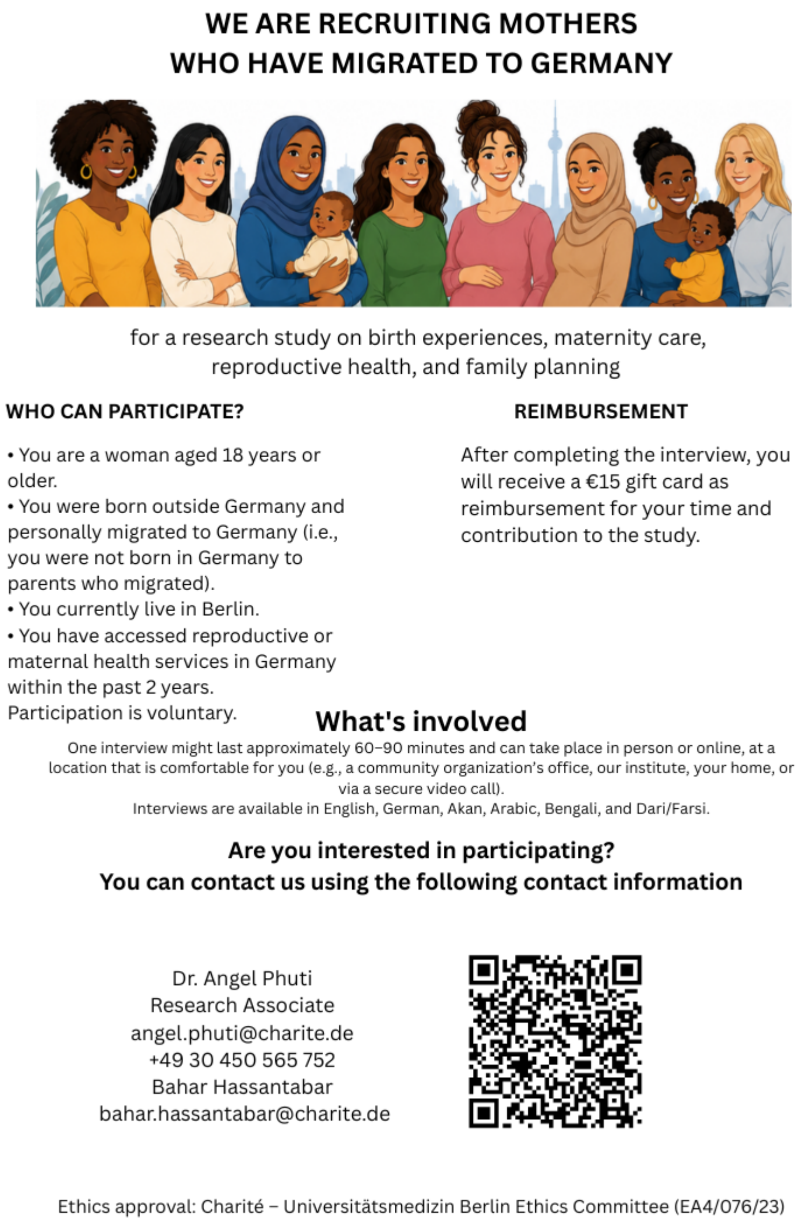

Figure S1. English-language recruitment flyer used to provide information about the study, eligibility criteria, voluntary participation, interview procedures, reimbursement, and study contact details.

از مادرانی که به آلمان مهاجرت کرده‌اند دعوت می‌کنیم در این پژوهش شرکت کنند

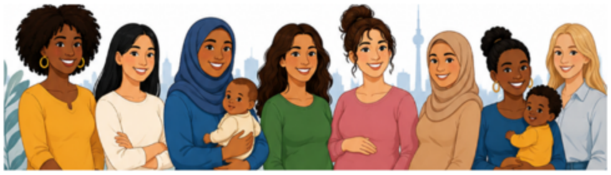

.این پژوهش درباره تجربه‌های زایمان، مراقبت‌های دوران بارداری و زایمان، سلامت باروری و تنظیم خانواده است

قدردانی از مشارکت شما

پس از انجام مصاحبه، به پاس زمانی که برای شرکت در این پژوهش اختصاص می‌دهید، یک کارت هدیه **۱۵** یورویی دریافت خواهید کرد.

چه کسانی می‌توانند در این پژوهش شرکت کنند؟

- بانوانی که ۱۸ سال سن یا بیش تر از ۱۸ سال دارند.
- خارج از آلمان متولد شده‌اید و خودتان به آلمان مهاجرت کرده‌اید (یعنی در آلمان از والدینی که مهاجرت کرده اند متولد نشده‌اید)
- در حال حاضر در برلین زندگی می‌کنید.
- در دو سال گذشته در آلمان از خدمات سلامت باروری یا مراقبت‌های مرتبط با بارداری و زایمان استفاده کرده‌اید.
- شرکت در این پژوهش کاملاً داوطلبانه است.

شرکت در پژوهش چگونه است؟

.مصاحبه ممکن است حدود ۶۰ تا ۹۰ دقیقه طول بکشد و می‌تواند به‌صورت حضوری یا آنلاین انجام شود
محل مصاحبه با توجه به راحتی شما انتخاب می‌شود؛ برای مثال، در دفتر یک سازمان اجتماعی، مؤسسه ما، منزل شما، یا از طریق یک تماس ویدیویی امن.
.مصاحبه‌ها به زبان‌های انگلیسی، آلمانی، آکان، عربی، بنگالی و دری/فارسی انجام می‌شوند

آیا مایل به شرکت در این پژوهش هستید؟
:برای دریافت اطلاعات بیشتر یا اعلام تمایل به شرکت، می‌توانید از طریق اطلاعات زیر با ما تماس بگیرید

Dr. Angel Phuti
پژوهشگر همکار
angel.phuti@charite.de
+49 30 450 565 752
Bahar Hassantabar
bahar.hassantabar@charite.de

:تأییده اخلاق پژوهش
کمیته اخلاق بیمارستان دانشگاهی شاریته برلین
(EA4/076/23)

Figure S2. Dari-language recruitment flyer provided as an example of the translated recruitment materials used to support multilingual participant recruitment.
