# Supplementary material for: “Do not leave me behind and do not put me in your box”: family-planning access and care needs among women with migration histories in Berlin, Germany—a mixed-methods study
Source: Reprod Health. 2026 Sep 25;23:175. doi: 10.1186/s12978-026-02450-6 (PMC13613642; doi:10.1186/s12978-026-02450-6)
Supplement: Supplementary file 1 [file 12978_2026_2450_MOESM1_ESM.zip › Supplementary file 2 _Semi-structured-interview-guide.pdf]

SEMI-STRUCTURED INTERVIEW GUIDE

Maternal, Reproductive, Family Planning, and Mental Health Care Experiences among Women with Migration Histories in Germany

Target group:
First-generation women with migration histories who have accessed maternal and/or reproductive healthcare in Germany

Estimated duration:
60–90 minutes

Interview approach:
Open-ended, exploratory, culturally responsive, non-judgmental, participant-centred, and trauma-informed

---

Annex 4. Consent Form for Women with Migration Histories

CHARITÉ
UNIVERSITÄTSMEDIZIN BERLIN

Version: 16 August 2024

Charité – Universitätsmedizin Berlin

Institute of International Health

Declaration of Consent

Study title:
....

I, the undersigned,

First name: ....
Surname: ....
Date of birth: ....

confirm that I have been informed, both verbally and in writing, by

Name of study researcher: ....

about the nature, purpose, significance, procedures, and potential risks of the above-mentioned scientific study. I have had sufficient opportunity to ask questions and to discuss any concerns with the study researcher.

I understand that my participation is voluntary. I may withdraw from the study at any time, without giving a reason and without any adverse consequences for me. I have also been informed of my rights concerning the further processing of my personal data, as described in the participant information provided for this study.

I confirm that I have received a copy of the written participant information and consent form, version dated **16 August 2024**.

Consent

I voluntarily agree to participate in this scientific study.

I consent to the following:

1. **The personal data required for the purposes of the study may be collected and processed by the study researcher as described in the participant information dated 16 August 2024. Study data will be recorded and processed in pseudonymized form, including on electronic data-storage systems.**
2. **Study findings may be published or presented in a form that does not directly identify me.**
3. **For the purposes of the study, my pseudonymized study data may be transmitted to the following authorized recipient(s):**

Name of recipient:
....

Address of recipient:
....
....

Place: ....

Date: .... / .... / 2024

Participant's signature:
....

Declaration by the Informing Researcher

I hereby confirm that I informed the participant about the study *Migrants' Maternal Health in Germany (MIMAHG)*, including its purpose, procedures, significance, and potential risks, both orally and in writing. I answered the participant's questions and provided her with a copy of the participant information and consent form.

Name of informing study physician/researcher:
....

Signature:
....

Date:
....

Using This Interview Guide

The questions in this guide are intended to be **open-ended and exploratory**. Encourage participants to describe their experiences across the continuum of care, including reproductive healthcare before pregnancy, pregnancy, childbirth, the postpartum period, family planning, and emotional well-being.

Use neutral probes where additional detail would be helpful. The guide is flexible: **not every question needs to be asked**, and the order may be adapted to the participant's experiences and the natural flow of the conversation.

Participants may decline to answer any question, request a pause or break, or stop the interview at any time.

Interviewers should avoid assumptions about participants' migration pathways, legal status, relationships, religion, reproductive intentions, contraceptive practices, or previous experiences. Sensitive topics should be approached gradually and only explored to the extent that the participant is comfortable.

Interviewer Introduction and Purpose of the Study

Hello, my name is **[name of interviewer]**. I am part of a research study exploring the maternal, reproductive, family planning, and mental healthcare experiences of women with migration histories in Germany.

The purpose of this study is to better understand women's experiences of accessing and receiving healthcare, including what has worked well, what challenges they have encountered, and how migration-related and broader social circumstances may have influenced their experiences.

You have been invited to participate because you are a first-generation woman with a migration history who has accessed maternal and/or reproductive healthcare in Germany.

There are no right or wrong answers. We are interested in your own experiences, views, and perspectives.

Interview Process

During this interview, I will ask you about different aspects of your healthcare experiences in Germany. Depending on what is relevant to you, we may discuss:

- maternal and reproductive healthcare before, during, and after pregnancy;
- navigating the German healthcare system;
- communication with healthcare professionals;
- respectful and culturally responsive care;
- family planning and contraception;
- emotional well-being and mental health;
- sources of personal, family, community, and professional support; and
- unmet needs or gaps in care.

The interview is expected to last approximately **60–90 minutes**.

You may choose not to answer any question, ask for a break, or stop the interview at any time. At the end, you will also have an opportunity to share anything else that you think is important.

Before we begin, we will confirm your preferred language and, if relevant, any interpretation arrangements.

Participation, Privacy, and Consent

Before starting the interview, I would like to make sure that you understand the purpose of the study and have had an opportunity to ask questions or raise any concerns.

Your privacy, comfort, and autonomy are important. You may decline to answer any question, request a break, or stop the interview at any time.

Do you have any questions before we begin?

If not, I would now like to confirm that you have provided written informed consent and agree to participate in this interview.

Data Privacy, Confidentiality, and Ethical Considerations

This study has received ethical approval from the **Ethics Committee of Charité – Universitätsmedizin Berlin (EA4/076/23)**. The necessary site-level permissions have also been obtained from participating recruitment organizations.

The study is conducted in accordance with the ethical principles of the **Declaration of Helsinki**.

Participation is entirely voluntary. Before participating, each person receives information about the purpose and procedures of the study, the topics that may be discussed during the interview, confidentiality and data-protection measures, and their rights as a research participant. Written informed consent is obtained before the interview begins.

Participants may decline to answer any question, request a pause or break, or withdraw from participation without adverse consequences.

Privacy and confidentiality will be protected throughout the study. Interviews will be conducted by trained researchers in the participant's preferred study language.

Where interviews are audio-recorded, recordings will be labelled using a **study identification code rather than the participant's name or other direct personal identifiers** and stored securely.

Interview transcripts, English translations, analytical files, and participant-level matrix data will be **pseudonymized**. This means that direct identifying information will be removed or replaced with study codes wherever possible. Access to these materials will be restricted to authorized members of the research team and will be limited to purposes related to this research.

Personal data collected and processed as part of the study will be handled in accordance with the **European Union General Data Protection Regulation (GDPR), Regulation (EU) 2016/679**, and the study's approved data-protection procedures.

Research findings will be reported in a manner designed to protect participants from being directly identified.

Before beginning the interview, the interviewer should ensure that the participant:

- has had an opportunity to ask questions;
- understands the study and what participation involves;
- understands how her data will be handled and protected; and
- has provided written informed consent.

Interviewer Confirmation Before Starting

Before we begin, I would like to confirm that you have received and understood the information about this study, including how your information will be protected and your rights as a participant.

Participation is voluntary. You may choose not to answer any question, take a break, or stop the interview at any time.

Your interview data will be pseudonymized and stored securely, with access limited to authorized members of the research team.

Do you have any questions about the study, confidentiality, or how your information will be used and protected?

Do you confirm that you have provided written informed consent and agree to participate in this interview?

If the interview will be audio-recorded

Do you also confirm that you agree to this interview being audio-recorded for research purposes?

Guide Structure

Section	Topic
A	Participant Demographic Information
B	Medical, Reproductive, and Family Planning Profile
1	Migration Background and Social Context
2	Maternal and Reproductive Healthcare Across the Continuum of Care
3	Navigation, Communication, and Understanding
4	Experience of Care, Relationships, and Support
5	Cultural Responsiveness, Safety, and Structural Conditions
6	Family Planning Knowledge, Practices, and Preferences
7	Access, Quality, and Autonomy in Family Planning Care
8	Maternal Mental Health, Emotional Well-Being, and Psychosocial Support
9	Unmet Needs, Care Gaps, Autonomy, and Recommendations
Closing	Closing Reflection

Section A. Participant Demographic Information

To be completed by the researcher.

Participant ID: ....
Interview date: ....
Interview location/mode: ....
Interviewer: ....

Socio-Demographic Characteristics

1. **Age:** .... years
2. **Country of origin:** ....
3. **Native/first language(s):** ....
4. **Years living in Germany:** ....

5. **Years living in Berlin:** ....
6. **Reason(s) for migration:** ....

   Examples: asylum/protection, family reunification, education, employment, other.

7. **Marital/relationship status:**

   ( ) Married
   ( ) Single
   ( ) Cohabiting
   ( ) Divorced/separated
   ( ) Widowed
   ( ) Other: ....

8. **Highest level of education completed:**

   ( ) No formal education
   ( ) Primary education
   ( ) Secondary education
   ( ) Tertiary education
   ( ) Other: ....

9. **Additional professional or vocational training:**

   ( ) Yes — please specify: ....
   ( ) No
   ( ) Not applicable

10. **Current employment status:**

( ) Employed
( ) Not employed
( ) Seeking employment
( ) Parental/maternity leave
( ) Other: ....

Section B. Medical, Reproductive, and Family Planning Profile

Complete sensitively. Participants may decline to answer any question.

1. **Gravida — total number of pregnancies:** ....
2. **Parity — total number of births:** ....
3. **Number of living children:** ....
4. **Number of miscarriages:** ....
5. **Number of abortions:** ....
6. **Current reproductive status:**

( ) Pregnant
( ) Postpartum
( ) Parenting a child under two years of age
( ) Not currently pregnant
( ) Recent abortion
( ) Other: ....

7. **Age at menarche, if relevant to the study:** ....
8. **Last menstrual period, if relevant and the participant is comfortable sharing:** ....
9. **Current or previous pregnancy complications:** ....

   Examples: gestational diabetes, pre-eclampsia, anaemia, hypertension, other.

10. **Existing health conditions/comorbidities:** ....
11. **Cervical cancer screening history and date, if known:** ....
12. **Currently sexually active:**

    ( ) Yes
    ( ) No
    ( ) Prefer not to say

13. **Current family planning or contraceptive method, if any:** ....
14. **Family planning or contraceptive methods used previously:** ....

    Examples: condoms or other barrier methods, withdrawal or other traditional methods, oral contraceptives or other hormonal methods, intrauterine devices, fertility-awareness methods, combined methods, other.

Section 1. Migration Background and Social Context

Focus: individual pathways, social circumstances, support networks, and sources of resilience.

- To begin, can you tell me a little about where you were born and raised?
- What brought you to Germany?
- How long have you been living in Germany?
- How would you describe your experience of living in Germany overall?
- How would you describe your experience as a woman with a migration history living in Germany?
- What have been some of the main challenges for you?
- What have been some positive experiences or sources of strength?

- Can you tell me about your family and social situation here?
- Who do you usually rely on when you need practical or emotional support?
- Are the people who support you mainly in Germany, in your country of origin, or elsewhere?

Section 2. Maternal and Reproductive Healthcare Across the Continuum of Care

Focus: WHO Quality of Care Framework; continuum of care; provision and experience of care.

Overall Experience

- Thinking about the maternal or reproductive healthcare you have received in Germany, how would you describe your experience overall?
- Looking across the different stages of care that are relevant to you — before pregnancy, during pregnancy, childbirth, and after birth — what stands out most?
- Would you describe your overall experience as mainly positive, mainly negative, mixed, or in another way? What makes you say that?

Before Pregnancy and Early Reproductive Care

- Before becoming pregnant, what reproductive or women's health services did you use in Germany, if any?

  Possible examples: gynaecological care, contraception counselling, cervical screening, fertility advice, or preconception care.

- How easy or difficult was it to access these services?
- How useful was the information or care you received?
- Did these services help you prepare for pregnancy or make decisions about your reproductive health? Why or why not?
- Was there any care or information that you wanted but could not obtain?

Prenatal/Antenatal Care

- Can you describe your experience of healthcare during pregnancy?
- How easy or difficult was it to find an appropriate healthcare provider?
- Did the care feel regular, well-organized, and timely?
- Were tests, examinations, referrals, and follow-up appointments explained clearly?
- Did you feel medically safe and well cared for during pregnancy? Why or why not?
- Did you receive enough information about your pregnancy, possible complications, childbirth, and what to expect after birth?
- Did you feel listened to, respected, and taken seriously?
- Were there moments when you felt confused, unsupported, or excluded from decisions about your care?
- Was there anything you needed during pregnancy that you did not receive?

Intrapartum/Childbirth Care

- Can you tell me about your experience of labour and birth?
- How safe did you feel during childbirth? What contributed to that feeling?
- Did healthcare professionals explain what was happening in a way that you could understand?
- When procedures or interventions were proposed, how were they explained to you?
- To what extent did you feel involved in decisions about your care?
- Did you feel respected, supported, and treated with dignity?
- Were there moments when you felt afraid, powerless, ignored, or unable to ask questions?
- How would you describe the emotional support you received during labour and birth?
- Was there anything you wish had been done differently?

Postnatal/Postpartum Care

- Can you describe the care you received after giving birth?
- What support did you receive after leaving the hospital?
- Did you receive enough information about your own physical recovery?
- Did you receive enough information about newborn care, breastfeeding or infant feeding, warning signs, emotional well-being, and follow-up care?
- How easy or difficult was it to access postnatal care, midwifery care, follow-up appointments, or other support services?
- Did you feel that your own health and well-being received enough attention after birth? Why or why not?
- Were there needs you had during the postpartum period that were not addressed?

Continuity and Coordination Across Stages

- Looking across pregnancy, childbirth, and the period after birth, where did care feel strongest?
- Where did care feel weakest or most difficult?
- Were there times when care felt disconnected or fragmented?
- Did you ever have to repeat your medical history or explain the same situation to several providers because information did not seem to be shared?
- How well did different services and healthcare professionals coordinate with one another?
- What would have made the overall care journey feel more continuous and coordinated?

Section 3. Navigation, Communication, and Understanding

Focus: continuity of care, health-system navigation, communication, language accessibility, and health literacy.

Navigating the Healthcare System

- How easy or difficult was it for you to understand how maternal and reproductive healthcare works in Germany?
- When you needed care, how did you know where to go or what to do next?

- Were referrals, appointments, and follow-up steps explained clearly?
- Were there times when you felt lost in the system or unsure about what you were expected to do?
- Did you ever miss, postpone, or avoid care because the system was difficult to navigate?
- Who or what helped you understand the system?

  Possible examples: partner, family, friends, community organization, NGO, midwife, doctor, social worker, or online resources.

Communication and Understanding

- How easy or difficult was it to understand the information you received during pregnancy, childbirth, or after birth?
- Did healthcare professionals explain medical terms, procedures, possible benefits and risks, or treatment options in a way that made sense to you?
- Were there times when you left an appointment without fully understanding what had been discussed?
- What did you do when something was unclear?

Language and Interpretation

- Did language differences ever affect your access to care or your experience of care? How?
- When interpretation was needed, were professional interpreters available or offered?
- If you relied on family members, friends, or other people to translate, how did this affect your privacy, confidence, ability to ask questions, or understanding?
- What would have made communication easier or more comfortable for you?

Rights and Available Services

- Did you receive information about your healthcare rights, available services, or entitlements?
- Which information was most helpful?
- What information do you wish you had received earlier?
- Where would you prefer to receive this kind of information?

Section 4. Experience of Care, Relationships, and Support

Focus: respect, trust, agency, person-centred care, relationships, and support networks.

Relationships with Healthcare Professionals

- How would you describe the way healthcare professionals treated you during pregnancy, childbirth, after birth, or other reproductive healthcare experiences?
- Did you feel respected as a person? What made you feel that way?
- Did you feel that your concerns were taken seriously?
- Did you feel comfortable asking questions or expressing your preferences?

- How much did you trust the healthcare professionals caring for you? What influenced that trust?
- To what extent were you involved in decisions about your care?
- Can you describe a situation in which you felt particularly involved — or not involved — in a decision?
- Were there times when you felt rushed, dismissed, or not listened to?
- Were there times when you felt especially well supported, reassured, or cared for? What made the difference?
- Did healthcare professionals pay attention to your emotional needs as well as your physical health?
- During important moments of care, particularly childbirth, how much control or agency did you feel you had?

Personal and Community Support

- Who supported you during pregnancy, childbirth, the postpartum period, or other reproductive health experiences?
- What role did your partner, relatives, friends, or other people close to you play?
- Were there times when you wanted more support than you had?
- Have community groups, NGOs, migrant organizations, religious or cultural organizations, peer groups, or online communities helped you with maternal or reproductive health? If so, how?
- What do you think about having a trained support person, such as a doula or birth companion, who understands your language or community background?
- What kind of support would you want such a person to provide?

Section 5. Cultural Responsiveness, Safety, and Structural Conditions

Focus: cultural responsiveness, non-discrimination, social determinants of health, migration-related conditions, and systems of power.

Cultural Responsiveness

- Did you feel that your cultural background, language, values, or preferences were understood and respected during your care?
- Were there practices, beliefs, or expectations related to pregnancy, childbirth, postpartum care, or reproductive health that were particularly important to you?
- Were healthcare professionals willing and able to acknowledge or accommodate these preferences?
- Can you describe anything that worked particularly well?
- Was there anything that you felt was misunderstood or not respected?

Discrimination and Unfair Treatment

- Did you ever feel that a healthcare professional made assumptions about you because of your nationality, language, religion, race, ethnicity, appearance, or migration history?

- Did you experience anything that felt discriminatory, stereotypical, disrespectful, or unfair?
- If so, how did that experience affect you?
- Did it influence your trust in healthcare professionals or your willingness to seek care later?

Migration and Structural Conditions

- Do you think your migration history has influenced the way you access or experience maternal or reproductive healthcare in Germany? How?
- Have factors such as residence or legal status, language, financial circumstances, housing, employment, health insurance, childcare responsibilities, or transport affected your ability to obtain care?
- Have you ever been uncertain about whether you were entitled to a particular healthcare service or form of support?
- How have broader life circumstances — for example financial pressure, unstable housing, childcare responsibilities, social isolation, or uncertainty about the future — affected your ability to look after your maternal or reproductive health?
- Which changes in healthcare or social services would make the biggest difference for women with migration histories?

Section 6. Family Planning Knowledge, Practices, and Preferences

Focus: informed choice, contraceptive autonomy, individual reproductive pathways, and preferences.

Understanding and Knowledge

- What does the term **family planning** mean to you?
- What does being able to decide whether and when to have children mean to you personally?
- What contraceptive or family planning methods have you heard about?
- Where have you learned about these methods?

  Possible examples: healthcare professionals, school, family, friends, internet, social media, apps, NGOs, or community groups.

- How well informed do you feel about family planning options and services available in Germany?
- Do you know where you can go for advice, counselling, or contraception?

Experiences with Contraceptive Methods

- Have you ever used a family planning or contraceptive method?
- Which methods have you used at different points in your life?
- Are you currently using a method? If so, which one?
- Have there been periods when you did not use any method? If you are comfortable sharing, what influenced that?
- Have you ever used more than one method at the same time or changed methods over time?
- What led you to change, stop, or begin a particular method?

- Have you used methods such as withdrawal, calendar tracking, fertility-awareness methods, or other approaches that you would describe as traditional or natural?
- What was your experience with these methods?

Preferences and Decision-Making

- How do you decide whether to use contraception and which method to use?
- What matters most to you when choosing a method?

  Possible examples: effectiveness, side effects, cost, convenience, partner preferences, breastfeeding, health concerns, religion, privacy, reversibility, or future fertility.

- Are there methods you prefer? What do you like about them?
- Are there methods you would prefer not to use? Why?
- Have your family planning needs or preferences changed over time?

  Possible examples: before or after migration, marriage or partnership changes, pregnancy, childbirth, breastfeeding, miscarriage, or abortion.

- Do you feel that the family planning methods available to you fit your health needs, preferences, and life circumstances? Why or why not?
- How have cultural beliefs, health priorities, personal values, or your sense of autonomy influenced your contraceptive choices?

Section 7. Access, Quality, and Autonomy in Family Planning Care

Focus: rights-based family planning, barriers and facilitators to access, informed choice, relationships, and power.

Access to Services

- How easy or difficult has it been for you to access family planning or reproductive health services in Germany?
- Can you describe what usually happens when you try to obtain contraception, counselling, or other reproductive healthcare?
- Where do you usually go for these services?

  Possible examples: gynaecologist, general practitioner, clinic, counselling centre, pharmacy, NGO, hospital, or online service.

- How easy or difficult is it to obtain an appointment?
- Are the services and methods you want usually available when you need them?
- Have cost, insurance rules, paperwork, or legal or administrative issues affected your options?
- Have language barriers made it more difficult to obtain family planning care?
- When language support was needed, was professional interpretation offered?

- Have you ever delayed, interrupted, or stopped using a contraceptive method because of cost, bureaucracy, availability, or difficulties navigating the healthcare system?

Information and Informed Choice

- When you discuss family planning with healthcare professionals, what kind of information do they usually provide?
- Are different methods, their possible benefits and risks, side effects, and alternatives explained clearly?
- Do you feel that you receive enough information to make a decision that is right for you?
- Do healthcare professionals usually discuss several options with you, or do they tend to recommend one particular method?
- Do you feel able to ask questions and take time to decide?

Autonomy and Reproductive Decision-Making

- Have you ever felt pressured or pushed towards a contraceptive method you did not want?
- Have you ever felt discouraged from using a method that you preferred?
- In general, have your reproductive preferences been respected?
- Can you describe an experience in which you felt that your preferences were particularly well respected — or not respected?
- How, if at all, do your partner's views or other people's opinions influence your family planning decisions?
- Do you feel able to express your own preferences freely? Why or why not?
- In your experience, is family planning generally treated as a woman's responsibility, a man's responsibility, or a shared responsibility? What makes you say that?
- What would respectful, high-quality family planning care look like to you?

Section 8. Maternal Mental Health, Emotional Well-Being, and Psychosocial Support

Focus: maternal mental health, psychosocial well-being, trauma-informed care, help-seeking, and access to support.

Interviewer Transition

I would now like to ask about emotional well-being during pregnancy and after birth.

Some of these questions are personal or sensitive. Please share only what you feel comfortable sharing. You may skip any question, ask for a break, or stop this part of the interview at any time.

Emotional Well-Being

- How would you describe how you felt emotionally during pregnancy?
- How did you feel emotionally after giving birth?
- Were there times when you felt emotionally well, supported, or able to cope? What helped during those times?

- Were there times when you felt overwhelmed, stressed, sad, anxious, lonely, or emotionally exhausted?
- Did these feelings change during pregnancy or after birth? In what way?
- Were there periods when you felt persistently sad, low, hopeless, very worried, or unable to relax?
- Did you experience difficulties with sleep, appetite, concentration, motivation, or feeling able to cope?
- How, if at all, did emotional distress affect your everyday life, relationships, ability to care for yourself, or ability to care for your baby?
- Were there times when your emotional distress felt especially severe or frightening?

Safety and Severe Distress

Ask sensitively and without requesting unnecessary detail. Follow the study safety protocol where required.

- Were there times when you felt that life was not worth living or had thoughts of harming yourself?
- If so, were you able to tell anyone or receive support?
- During pregnancy or after birth, were there times when you felt unsafe at home or in a close relationship?
- If you are comfortable discussing it, was support available to you?

Difficult or Traumatic Experiences

- Do you think difficult experiences in your past or present circumstances affected your emotional well-being during pregnancy or after birth? How?

  Possible examples include migration-related stress, bereavement or separation, violence, trauma, miscarriage, abortion, a difficult or traumatic birth, social isolation, uncertainty about legal status, or financial pressure.

- Were there particular circumstances that made coping more difficult?
- Were there things that helped you feel safer, stronger, or more supported?

Help-Seeking and Mental Healthcare

- When you felt stressed or emotionally unwell, did you seek help from anyone? Who?
- Did anyone in the healthcare system ask you about your mood, stress, anxiety, or emotional well-being during pregnancy or after birth?
- Were you ever offered or referred for counselling, psychological support, psychiatric care, medication, peer support, or another form of assistance?
- If you tried to access support, how easy or difficult was that?
- What made access easier or more difficult?
- Did maternal healthcare professionals pay attention to your emotional well-being as well as your physical health?
- Did you feel comfortable discussing emotional or mental health concerns with healthcare professionals? Why or why not?

- Were there times when you felt that emotional distress was ignored, minimized, misunderstood, or treated as unimportant?
- What should maternal healthcare services do differently to support women's emotional well-being and mental health during pregnancy and after birth?

Section 9. Unmet Needs, Care Gaps, Autonomy, and Recommendations

Focus: synthesis across maternal healthcare, reproductive autonomy, family planning, mental health, and health-system improvement.

Unmet Reproductive Needs

- Have there been times when you wanted to avoid pregnancy, delay pregnancy, become pregnant, or plan a pregnancy differently but were unable to do so in the way you wanted?
- What made it difficult?

  Possible examples: lack of information, provider attitudes, language barriers, cost, difficulties accessing services, partner influence, concerns about side effects, insurance or legal barriers, or limited availability of preferred methods.

Gaps in Care

- Looking at maternal and reproductive healthcare more broadly, where do you think the biggest gaps are for women with migration histories in Germany?
- Are there particular stages — before pregnancy, during pregnancy, childbirth, postpartum, family planning, or mental healthcare — where you think women need more support?
- Which needs do you think healthcare services currently overlook?

Autonomy, Dignity, and Quality

- In your view, what does it mean for a woman to have genuine choice, control, dignity, and support in maternal and reproductive healthcare?
- What makes a healthcare experience feel respectful and safe?
- What makes it feel disrespectful or unsafe?

Recommendations

- What would help women with migration histories obtain better maternal, reproductive, family planning, and mental healthcare in Germany?
- What could healthcare professionals do differently?
- What could clinics and hospitals do differently?
- What could health insurers, government services, community organizations, or NGOs do differently?
- What kind of information, support, or services would make the biggest difference for you personally?

- What would you recommend to improve maternal healthcare and family planning services for other women in circumstances similar to yours?

Closing Reflection

Before we finish, I would like to give you an opportunity to mention anything we may not have discussed.

Is there anything else about your experiences with maternal care, reproductive health, family planning, emotional well-being, or healthcare in Germany that you think is important for us to understand?

Is there anything you expected me to ask about that I did not ask?

Thank you very much for your time and for sharing your experiences with us.

The information you have provided will contribute to our understanding of how women with migration histories experience maternal, reproductive, family planning, and mental healthcare in Germany. Your experiences and perspectives will help us identify what works well, where gaps or challenges exist, and what could be improved to make healthcare more accessible, responsive, respectful, and supportive.

We greatly appreciate your contribution to this study.

END OF INTERVIEW

Suggested Neutral Probing Prompts for Interviewers

Use probes selectively rather than after every response.

- “Can you tell me more about that?”
- “Can you give me an example?”
- “What happened next?”
- “What was that experience like for you?”
- “How did you feel at the time?”
- “What do you think contributed to that?”
- “How did you respond?”
- “What made that easier or more difficult?”
- “Who was involved or supported you?”
- “Was there anything you wanted to happen differently?”
- “What would have helped at that moment?”
- “How did that affect what you did afterwards?”
- “Is there anything else about that experience that feels important?”

Avoid probes that suggest there is a particular answer the participant is expected to give.

Interviewer Notes

- Use inclusive, respectful, and non-stigmatizing language throughout.
- Follow the participant's terminology for her identity, experiences, relationships, and reproductive history where appropriate.
- Avoid assumptions about migration pathway, residence or legal status, nationality, ethnicity, religion, relationship dynamics, contraceptive use, fertility intentions, pregnancy intentions, or family structure.
- Prioritize participant privacy, comfort, dignity, and autonomy.
- Remind participants when necessary that they may decline to answer any question.
- Do not pressure participants to provide details about traumatic or highly personal experiences.
- Sensitive topics — including miscarriage, abortion, infertility, traumatic birth, violence, discrimination, reproductive coercion, self-harm, and mental health difficulties — should be introduced gently and explored only to the extent that the participant wishes.
- Where interpreters are used, maintain confidentiality and participant-centred communication. Address questions directly to the participant rather than to the interpreter.
- Avoid using family members as interpreters for highly sensitive topics wherever appropriate professional interpretation is available.
- Pay attention to transitions between stages of care, particularly where participants describe fragmentation, confusion, repeated referrals, or discontinuity.
- Allow participants to define concepts such as "quality," "support," "respect," "safety," "good care," and "autonomy" in their own words.
- Distinguish between what the participant personally experienced and what she has heard from others.
- Avoid suggesting that negative experiences necessarily resulted from migration status or cultural background; allow participants to describe what they believe influenced their experiences.
- Do not challenge, correct, or clinically interpret a participant's account during the research interview unless immediate safety requires action under the study protocol.
- Where a participant describes a positive or negative experience, explore what specifically contributed to that experience.
- If a participant becomes visibly distressed, stop asking substantive questions and follow the distress protocol below.
- If a participant discloses an immediate risk of self-harm or current danger, follow the approved study safety and referral protocol.

Safety and Distress Protocol

If a participant becomes distressed during the interview:

1. **Pause the interview.**
   Stop the current line of questioning and acknowledge that the participant may take as much of a break as she needs within the interview setting.
2. **Reaffirm choice and control.**
   Remind the participant that she may decline to answer the question, change the topic, take a break, or stop the interview entirely.
3. **Check whether she wishes to continue.**
   Continue only if the participant clearly indicates that she would like to do so.
4. **Avoid unnecessary probing.**
   Do not ask for additional details about traumatic experiences merely for completeness if those details are not essential to the research question.
5. **Follow the approved safety and referral procedure when required.**
   If the participant discloses an immediate risk of self-harm, current danger, or another circumstance covered by the study's safety protocol, follow the approved escalation and referral procedure.
6. **Document according to the approved study procedure.**
   Record only the information required by the study's safety and research procedures, while protecting the participant's confidentiality.

Emergency Study Contacts

Dr Angel Phuti, Research Associate
Email: angel.phuti@charite.de
Telephone: +49 30 450 565 752

Bahar Hassantabar, Research coordinator
Email: bahar.hassantabar@charite.de
