# Supplementary material for: “Do not leave me behind and do not put me in your box”: family-planning access and care needs among women with migration histories in Berlin, Germany—a mixed-methods study
Source: Reprod Health. 2026 Sep 25;23:175. doi: 10.1186/s12978-026-02450-6 (PMC13613642; doi:10.1186/s12978-026-02450-6)
Supplement: Supplementary file 1 [file 12978_2026_2450_MOESM1_ESM.zip › Supplementary File 3_Qualitative_Codebook.pdf]

SUPPLEMENTARY FILE 3

Qualitative Codebook

Integrated UNFPA-aligned family-planning care-needs and ETR Health Equity coding frameworks

Contents. Part I presents the UNFPA-aligned codebook for family-planning care needs. Part II presents the integrated ETR Health Equity theoretical and operational codebook. Each part includes the complete parent-level or framework-level definitions, coding decision rules, study subcodes, and illustrative quotations supplied for analysis.

Part I. UNFPA-Aligned Family-Planning Care-Needs Codebook

P1. Communication and Information

**Parent code**	P1. Communication and Information
**UNFPA alignment**	UNFPA level: Primarily Service Delivery – Quality Information and Services and Individual – Empowered and Satisfied Client; also relevant to Laws & Policy. Relationship: Reflects UNFPA's requirement for comprehensive, unbiased, scientifically accurate, understandable, and accessible contraceptive information that supports informed decision-making and is adapted to individuals' language, abilities, and circumstances. Components: Accessibility; informed decision-making; quality; agency/autonomy/empowerment; non-discrimination and equality.
**Operational definition**	Experiences or expressed needs concerning the availability, clarity, comprehensibility, timing, and individual relevance of family planning information and counselling, including proactive information provision, language and translation support, and health-literacy needs. Also includes self-directed, digital, or alternative information-seeking when formal healthcare information is insufficient.

Child domains

1.1 Clear, Understandable, and Relevant Contraceptive Information

Child definition

Apply when participants describe needs or experiences related to the availability, clarity, completeness, comprehensibility, and relevance of information about contraception, side effects, reproductive options, or family planning services.

Illustrative quotation(s)

"In Germany, doctors don't usually give you a lot of information."

1.2 Proactive Counselling and Sufficient Consultation Time

Child definition

Apply when participants describe a need for healthcare providers to proactively initiate family planning discussions and provide sufficient consultation time to discuss contraceptive options, ask questions, express preferences, and receive clarification.

Illustrative quotation(s)

"When you go to the doctor, the visit always feels rushed." / "In my country they give birth control information right after the baby is born, but here nobody talked to me about it unless I asked."

1.3 Self-Directed Information-Seeking and Digital Tools

Child definition

Apply when participants independently seek family planning information through their own research, websites, artificial intelligence, fertility-tracking applications, translation tools, or other digital resources, particularly when information from formal healthcare services is insufficient.

Illustrative quotation(s)

> *"I had to do a lot of research myself." / "ChatGPT has changed everything… That is how I am surviving now." / "I use apps to track my period and understand ovulation so I can avoid having sex on those days."*

1.4 Language, Translation, and Health-Literacy Support

Child definition

Apply when language proficiency, lack of multilingual information, difficulty interpreting health information, or limited support for navigating reproductive health information affects participants' ability to understand and use family planning services.

Illustrative quotation(s)

> *"Most of the information is in German, and since my German is only at a B1 level, it was a struggle to understand everything."*

P2. Continuity and Affordability

**Parent code**	P2. Continuity and Affordability
**UNFPA alignment**	UNFPA level: Primarily Service Delivery – Quality Information and Services and Laws & Policy – Enabling Legal and Policy Environment; also relevant to Individual – Empowered and Satisfied Client. Relationship: Reflects UNFPA's principle of accessibility, including economic and practical accessibility, and the requirement that contraception be affordable, available when needed, and free from unnecessary administrative, medical, or other access barriers. Components: Accessibility; availability; non-discrimination and equality; accountability; agency/autonomy.
**Operational definition**	Experiences or expressed needs concerning the ability to obtain and continue using a preferred contraceptive method without excessive financial costs, repeated appointments, administrative requirements, caregiving demands, or other practical burdens. Includes both initial access and sustained access over time.

Child domains

2.1 Affordable Contraception and Reduced Out-of-Pocket Costs

Child definition

Apply when participants describe financial barriers to contraception, including out-of-pocket payments, limited income, unemployment, or other financial circumstances that make preferred contraceptive methods difficult to afford.

Illustrative quotation(s)

"Because I'm not working it can be expensive." / "I had to pay for them."

2.2 Continuity of Preferred Contraceptive Methods

Child definition

Apply when participants describe the need to obtain and continue using a contraceptive method that suits their preferences and circumstances without interruptions caused by financial, prescription, supply, or other access barriers.

Illustrative quotation(s)

"Sometimes I ask people to bring them from Portugal." / "I have to go back again to get a new prescription."

2.3 Reduced Administrative and Appointment Burden

Child definition

Apply when participants describe unnecessary or burdensome prescription renewals, repeated appointments, paperwork, healthcare-system procedures, waiting times, or other administrative requirements associated with accessing contraception.

Illustrative quotation(s)

"My doctor only gave me a prescription for six months, so I have to go back again to get a new prescription." / "It feels like a lot of wasted time."

2.4 Practical Accessibility and Caregiving-Related Barriers

Child definition

Apply when practical circumstances, including time, travel, caregiving responsibilities, migration-related circumstances, unfamiliarity with the healthcare system, or limited support networks, make family planning services difficult to access or maintain.

Illustrative quotation(s)

"Most of us migrant women don't really know where to find the information."

P3. Respect and Shared Responsibility

**Parent code**	P3. Respect and Shared Responsibility
**UNFPA alignment**	UNFPA level: Primarily Individual – Empowered and Satisfied Client and Service Delivery – Quality Information and Services; also linked

	to Community – Supportive Community and Social and Gender Norms. Relationship: Reflects UNFPA's emphasis on bodily autonomy, agency, informed decision-making, and respect for individual preferences, including the right to accept or refuse contraception without pressure or coercion. It also relates to gender norms surrounding the distribution of contraceptive responsibility. Components: Agency/autonomy/empowerment; informed decision-making; acceptability; quality and respectful care; non-discrimination and equality; participation.
**Operational definition**	Experiences or expressed needs concerning respect for contraceptive preferences, reproductive autonomy and informed refusal, collaborative rather than directive decision-making, and a more equitable distribution of contraceptive responsibility between partners and genders.

Child domains

3.1 Respect for Contraceptive Preferences and Informed Refusal

Child definition

Apply when participants describe the need for healthcare providers to recognise and respect their contraceptive preferences, including their right to refuse an unwanted method without pressure, persuasion, or coercion.

Illustrative quotation(s)

"My doctor has been trying to tell me to get a coil." / "I told her that I would get back to her, but I do not plan to do it."

3.2 Reproductive Autonomy and Shared Decision-Making

Child definition

Apply when participants emphasise bodily autonomy and expect contraceptive decisions to be made collaboratively through discussion between the individual and the healthcare provider rather than through one-sided recommendations.

Illustrative quotation(s)

"I do not want to put anything else in my body." / "You also need to listen to what works for me so that we can meet somewhere in the middle."

3.3 Individualised, Non-Prescriptive Counselling

Child definition

Apply when participants describe a need for providers to listen to their individual circumstances, experiences, concerns, and preferences and to avoid standardised or prescriptive approaches to contraceptive counselling.

Illustrative quotation(s)

"You should not put me in your box." / "You also need to listen to what works for me."

3.4 Equitable Partner and Gender Responsibility for Contraception

Child definition

Apply when participants discuss the unequal distribution of contraceptive responsibility between women and men, including women's disproportionate physical, emotional, practical, or organisational burden, limited male contraceptive options, and unequal exposure to contraceptive side effects.

Illustrative quotation(s)

"Most contraception methods are more focused on women than on men." / "For men, there are very few methods." / "The side effects are also mostly on women — hormonal side effects or birth-control side effects."

P4. Cultural and Life-Course Responsiveness

**Parent code**	P4. Cultural and Life-Course Responsiveness
**UNFPA alignment**	UNFPA level: Primarily Service Delivery – Quality Information and Services and Individual – Empowered and Satisfied Client; also relevant to Laws & Policy. Relationship: Reflects UNFPA's principles of acceptability, non-discrimination, and quality, including culturally respectful, gender-sensitive, and life-course-responsive services that accommodate individual needs and preferences rather than generalised assumptions. Components: Acceptability; non-discrimination and equality; quality; accessibility; agency/autonomy/empowerment; informed decision-making.
**Operational definition**	Experiences or expressed needs for individualised family planning care that takes account of cultural and migration-related contexts, previous healthcare experiences, and changing reproductive circumstances across the life course, including pregnancy, the postpartum period, breastfeeding, and other reproductive transitions.

Child domains

4.1 Recognition of Previous Healthcare Experiences

Child definition

Apply when participants' previous experiences with healthcare or family planning services, particularly in their countries of origin, shape their expectations, preferences, or evaluations of family planning care in Germany.

Illustrative quotation(s)

"In my country they give birth control information right after the baby is born, but here nobody talked to me about it unless I asked."

4.2 Migration- and Culturally Responsive Care

Child definition

Apply when participants describe a need for healthcare providers to consider cultural background, ethnicity, migration history, language, and related contextual factors while avoiding stereotyping and recognising individual differences.

Illustrative quotation(s)

"When ethnicity and other factors contribute, there can be differences. I think this should be considered while treating patients." / "Everybody is different." / "You should not put me in your box."

4.3 Responsiveness to Reproductive and Life-Course Stages

Child definition

Apply when pregnancy, the postpartum period, breastfeeding, age, fertility intentions, or other reproductive and life-course stages influence participants' contraceptive needs, preferences, or need for family planning counselling.

Illustrative quotation(s)

"Right now, I am breastfeeding and using condoms." / "In my country they give birth control information right after the baby is born."

4.4 Counselling Adapted to Changing Contraceptive Needs and Circumstances

Child definition

Apply when participants describe contraceptive needs or preferences that may change over time and express a need for counselling about methods appropriate to their current circumstances as well as options that may become suitable in the future.

Illustrative quotation(s)

"I feel like I should probably talk to a doctor about other options that might work better for me later."

Part II. Integrated ETR Health Equity Theoretical and Operational Codebook

Systems of Power

**Framework component**	Systems of Power
**Full theoretical definition**	Systems of Power refers to the policies, institutional structures, processes, practices, and broader forms of power that determine who has access to resources, services, opportunities, and protection and who does not. These systems can generate, reproduce, maintain, or reduce inequities. The construct includes formal laws and policies as well as institutional routines, administrative procedures, allocation of resources, discrimination, and structural manifestations of racism, sexism, classism, ableism, homophobia, transphobia, and other forms of unequal power. The central theoretical concern is not simply that an individual encounters difficulty, but that the difficulty is produced or shaped by the organization of institutions and the unequal distribution of resources and opportunities.
**Operational definition in this study**	Systems of Power was operationalized as the structural, institutional, legal, administrative, economic, and material conditions that shaped women's ability to access and experience family-planning and reproductive-health care. It included racism and racialization within healthcare, unequal treatment, uncertainty about healthcare entitlements, insurance or eligibility arrangements, institutional procedures, costs of contraception or treatment, and housing or material insecurity. These conditions were examined in terms of how they affected whether participants could obtain and afford services and whether they felt recognized, safe, and fairly treated within healthcare institutions.

Study subcodes

SYS-1: Racism, discrimination, and racialization

Coding decision rule

Code when the primary influence arises from institutional or structural racialization, racism, stereotyping, othering, or unequal treatment related to race, ethnicity, skin colour, migration status, or national background.

Subcode definition

Used when participants described interpersonal or structural racism, stereotyping, othering, unequal treatment, or assumptions associated with race, ethnicity, skin colour, migration status, or national background. The code allows both specific discriminatory encounters and participants’ descriptions of racism as a wider structural condition.

Illustrative quotation or indicator

"The negative here is racism and discrimination. This is structural. When you are Black, people assume things about you."

SYS-2: Institutional and administrative access

Coding decision rule

Assigned when the institutional issue concerned rules, eligibility, insurance, administrative procedures, service entitlements, appointments, or uncertainty about how to enter or use the healthcare system, rather than racialized treatment or financial circumstances.

Subcode definition

Used when healthcare access was shaped by eligibility rules, legal status, insurance arrangements, administrative procedures, service entitlements, appointment systems, or uncertainty about how healthcare services operated.

Illustrative quotation or indicator

"I did not know which services I am actually entitled to."

SYS-3: Financial and material conditions

Coding decision rule

Selected when the relevant structural constraint or resource involved money or material circumstances, such as cost, income, housing, food security, transportation, or other resources affecting access to contraception, pregnancy care, or reproductive-health services.

Subcode definition

Used when costs, income, housing, food security, transport, or other material resources influenced reproductive health, pregnancy experiences, contraceptive access, or use of healthcare services.

Illustrative quotation or indicator

"Birth control is not free… I would have to pay… it costs more than 300."

SYS-4: Equity, rights, and institutional responsiveness

Coding decision rule

Applied when participants moved beyond describing a barrier and explicitly raised fairness, rights, unequal institutional treatment, challenges to unfair practices, or whether institutions responded appropriately to women's needs.

Subcode definition

Used when participants identified unequal institutional practices, asserted healthcare rights, challenged unfair treatment, or described whether healthcare organizations responded equitably to diverse reproductive-health needs.

Illustrative quotation or indicator

"I finally got up and asked… 'I've been here before these people, so why aren't you attending to me?'"

Relationships and Networks

**Framework component**	Relationships and Networks
**Full theoretical definition**	Relationships and Networks refers to the social connections and support structures through which people experience assistance, influence, pressure, belonging, stigma, conflict, or isolation. These relationships may include partners, family members, relatives, friends, peers, community members, schools, workplaces, and other social networks. The framework recognizes that social relationships may either protect and facilitate health or create constraints and harms.
**Operational definition in this study**	Relationships and Networks was operationalized as the ways partners, family members, relatives, friends, peers, translators, community organizations, NGOs, and digital communities influenced family-planning decisions and access to reproductive-health services. Relationships could facilitate access through information, emotional support, translation, childcare, navigation, or referrals, but could also constrain access through social isolation, lack of practical support, stigma, discouraging information, or circulation of fears and misinformation.

Study subcodes

NET-1: Family and social support

Coding decision rule

Apply where the presence, absence, or quality of support from partners, family, relatives, friends, or other trusted people influences wellbeing, decisions, or healthcare access.

Subcode definition

Used when the presence, absence, or quality of support from partners, family, relatives, friends, or trusted individuals influenced emotional wellbeing, reproductive decisions, or healthcare access.

Illustrative quotation or indicator

> *"I have no one here to help me with anything… I don't have friends or relatives here to support me."*

NET-2: Practical and navigation support

Coding decision rule

Chosen where another person provides concrete practical assistance, such as interpretation, childcare, transport, making or attending appointments, communication, locating services, or healthcare navigation.

Subcode definition

Used when another person assisted with interpretation, transportation, childcare, appointments, communication with providers, locating services, or navigating the healthcare system.

Illustrative quotation or indicator

"The only help we get sometimes is from translators who work in the refugee housing."

NET-3: Community and organizational support

Coding decision rule

Apply when support comes specifically from an organized community or civil-society source, such as an NGO, refugee organization, advocate, community organization, or community worker.

Subcode definition

Used when NGOs, refugee organizations, community organizations, advocates, or community workers provided reproductive-health information, referrals, advocacy, accompaniment, or access support.

Illustrative quotation or indicator

"Without the help of these organizations, many women would not know where to go or how to get care."

NET-4: Peer and digital knowledge networks

Coding decision rule

Apply when knowledge or support circulates through peers, messaging platforms, online communities, social media, or informal digital/social networks.

Subcode definition

Used when women exchanged experiences, advice, emotional support, recommendations, or reproductive-health information through peers, social groups, messaging platforms, online communities, or social media.

Illustrative quotation or indicator

"I started an online support group where women could share their experiences and information with each other."

Individual Factors

**Framework component**	Individual Factors
**Full theoretical definition**	Individual Factors refers to the knowledge, attitudes, beliefs, skills, coping strategies, confidence, self-efficacy, decision-making capacities, and behaviours through which an individual understands and responds to her social and environmental circumstances. This sphere does not conceptualize the individual in isolation from structural or relational influences; rather, it captures the personal

	resources and responses through which those circumstances are interpreted and acted upon.
**Operational definition in this study**	Individual Factors was operationalized as participants' personal knowledge and understanding of family planning and healthcare, language proficiency, contraceptive beliefs and perceptions, information-seeking practices, previous experiences, confidence, personal agency, and capacity to advocate for themselves. These resources affected participants' ability to understand contraceptive options, communicate with providers, book appointments, navigate services, obtain second opinions, and make reproductive-health decisions consistent with their preferences.

Study subcodes

IND-1: Contraceptive knowledge and perceptions

Coding decision rule
Apply to beliefs, expectations, concerns, perceived risks, side-effect fears, misinformation, or previous knowledge concerning contraception when these shape preferences or decisions.

Subcode definition
Used for beliefs, expectations, concerns, perceived risks, side-effect fears, misinformation, or previous knowledge concerning contraception when these shaped contraceptive preferences or decisions.

Illustrative quotation or indicator
> *"I've heard so many horror stories how it just messes up your hormones… I just never ended up taking it."*

IND-2: Health literacy and language

Coding decision rule
Apply when the relevant personal resource is the participant's own ability to understand health information or communicate in the healthcare language. The participant's own language proficiency belongs here; interpretation supplied by another person belongs under NET-2.

Subcode definition
Used when ability to understand reproductive-health information, terminology, healthcare instructions, or the dominant healthcare language affected understanding, communication, or service navigation.

Illustrative quotation or indicator
> *Difficulty understanding reproductive-health information or communicating with providers because of language or unfamiliar terminology.*

IND-3: Information seeking and knowledge development

Coding decision rule

Apply when the participant actively searches for, compares, checks, verifies, or develops information herself, rather than simply reporting what she already knows or what others tell her.

Subcode definition

Used when women independently sought, compared, verified, or developed knowledge concerning contraception, pregnancy, reproductive health, or available services using formal or informal sources.

Illustrative quotation or indicator

"I pretty much research on my own."

IND-4: Reproductive agency and self-advocacy

Coding decision rule

Selected when the account emphasizes choice, confidence, expressing preferences, asking questions, challenging advice, seeking another opinion, changing providers, or advocating for oneself.

Subcode definition

Used when women expressed confidence in identifying preferences, making reproductive decisions, asking questions, communicating needs, challenging recommendations, or advocating for themselves within healthcare encounters.

Illustrative quotation or indicator

"I'm very confident about the choices that I make… I know what I want."

Physiological and Psychological Pathways

**Framework component**	Physiological Pathways (empirically operationalized primarily as Psychological Pathways in this study)
**Full theoretical definition**	In the framework, Physiological Pathways encompasses biological, physical, cognitive, and psychological processes and capacities that influence health. It also captures mechanisms through which social and environmental exposures become embodied or affect biological, developmental, cognitive, or psychological functioning. Relevant processes may include chronic stress responses, psychological functioning, cognitive functioning, brain development, immune and neuroendocrine processes, physical functioning, disability, and the biological or developmental consequences of prolonged adversity.
**Operational definition in this study**	In this family-planning study, the empirical material within this theoretical sphere was concentrated primarily on psychological rather than biological mechanisms. It was therefore operationalized as Psychological Pathways: how trauma, fear, emotional distress,

	reproductive loss, displacement, painful memories, and feelings of psychological safety shaped participants' reproductive-health experiences and, where evident in the data, family-planning decisions or healthcare engagement. The study did not assume that trauma automatically influenced family-planning behaviour.

Study subcodes

PSY-1: Trauma and reproductive fear

Coding decision rule

Apply when past trauma, violence, reproductive loss, displacement, female genital cutting, or other harmful experiences generate fear, anxiety, uncertainty, or concern about pregnancy, childbirth, contraception, or reproductive healthcare.

Subcode definition

Used when past trauma, violence, reproductive loss, displacement, female genital cutting, or other harmful experiences generated fear, anxiety, uncertainty, or concern about pregnancy, childbirth, contraception, or reproductive healthcare.

Illustrative quotation or indicator

> *"Sometimes I wonder if I will even be able to give birth because of the cutting. That is one of my biggest fears."*

PSY-2: Emotional and psychological distress

Coding decision rule

Apply where the dominant phenomenon is the participant's emotional or psychological distress itself, including anxiety, grief, fear, unresolved painful experiences, or unwillingness to disclose distressing memories.

Subcode definition

Used for descriptions of emotional pain, anxiety, distress, grief, fear, or unresolved psychological experiences associated with reproductive health or migration experiences. It also includes reluctance to disclose painful experiences.

Illustrative quotation or indicator

> *"No one ever asked me, and honestly, I prefer not to share because it brings me pain."*

PSY-3: Recognition of emotional needs in care

Coding decision rule

Apply when the participant discusses whether the healthcare encounter recognized, explored, ignored, or responded to her emotional needs.

Subcode definition

Used when participants described whether healthcare encounters recognized, explored, overlooked, or responded to emotional and psychological needs.

Illustrative quotation or indicator

> *"You can have the best medical support, but if someone doesn't really listen or understand the emotional side, then something important is missing."*

PSY-4: Psychological safety with empathetic care

Coding decision rule

Apply when provider empathy, communication, listening, validation, sensitivity, or trauma-informed interaction influences psychological comfort, trust, safety, or willingness to communicate reproductive-health needs.

Subcode definition

Used when provider communication, empathy, listening, validation, sensitivity, or trauma-informed practices influenced feelings of trust, comfort, safety, and willingness to discuss reproductive needs.

Illustrative quotation or indicator

> *"It's one thing to be just a doctor, but another thing to have an empathetic doctor in the medical field."*
