# Supplementary material for: “Do not leave me behind and do not put me in your box”: family-planning access and care needs among women with migration histories in Berlin, Germany—a mixed-methods study
Source: Reprod Health. 2026 Sep 25;23:175. doi: 10.1186/s12978-026-02450-6 (PMC13613642; doi:10.1186/s12978-026-02450-6)
Supplement: Supplementary file 1 [file 12978_2026_2450_MOESM1_ESM.zip › Supplementary File 4_Quantitative_Codebook.pdf]

SUPPLEMENTARY FILE 4

Quantitative Codebook

Empty data shell, variable dictionary, coding decisions, and value labels

**Scope**	48 analytic variables spanning reproductive history, socioeconomic and legal determinants, migration context, material/biological/linguistic determinants, unmet family-planning needs, and four Health Equity Framework pathways.
**Unit of analysis**	One participant or qualitative interview per row in the accompanying Excel data shell.
**Administrative field**	participant_id is used for record linkage and is not included in the count of 48 analytic variables.
**Binary convention**	1 = mechanism or unmet need evidenced; 0 = not evidenced. A zero does not necessarily mean the mechanism was absent from the participant’s life.
**Missing data**	Use 9 only where explicitly defined. Leave continuous or text variables blank when missing.

Document structure

Section 1 provides global coding rules. Section 2 presents the complete variable dictionary in the order used by the Excel data-entry shell. Section 3 consolidates categorical value labels.

1. General Coding Rules

Unmet needs

Mention does not equal unmet need. Assign 1 only for an explicit or clearly implied gap, barrier, difficulty, dissatisfaction, inadequate support, or desired improvement.

Missing values

Never use 0 for missing where zero is substantive. Use 9 only where defined; otherwise leave continuous/text values blank.

Explicit evidence

Code what is reported; do not calculate or infer miscarriage, legal status, migration reason, language proficiency, discrimination, or other mechanisms from demographics.

Current vs ever method

current_fp_method records use at interview; ever_fp_method records lifetime experience, including current use.

Binary pathways

1 = mechanism evidenced; 0 = not evidenced. Zero does not necessarily mean the mechanism did not exist in the participant's life.

Barrier/facilitator independence

Do not treat barrier and facilitator variables as opposites; both can be 0 when neither is evidenced.

Mechanism over topic

Code the mechanism, not a topic mention. Costs must restrict access to count as financial_barriers; side effects must shape a decision to count as perceived_contraceptive_side_effects.

Co-coding

Co-coding is permitted when multiple mechanisms are evidenced in the same passage.

Uncertainty

Review the surrounding passage. If the mechanism cannot be established confidently, code 0 for binary variables or 9/blank according to the variable's missing rule.

Combined construct warning

childcare_burden_stigma is 1 when either childcare constraint or interpersonal stigma is evidenced; consider splitting this variable in future analyses.

2. Variable Dictionary

Reproductive history and proximate reproductive factors

1. Number of children (number_children)	
**Variable type**	Integer
**Allowed values**	0 = no living children; 1 = one; 2 = two; 3 = three.
**Missing value**	9 = unclear/missing
**Definition**	Number of living children reported by the participant at the time of interview.
**Coding decision rule**	Count living children only. Do not count miscarriages, abortions, or other pregnancy losses as children. If the number cannot be established from the interview, code 9 = unclear/missing.

2. Total pregnancies (total_pregnancies)	
**Variable type**	Integer
**Allowed values**	0 = none; 1 = one; 2 = two; 3= three; 4 = four.
**Missing value**	9 = unclear/missing
**Definition**	Total number of pregnancies the participant has experienced, regardless of outcome.
**Coding decision rule**	Count all reported pregnancies, including pregnancies resulting in a live birth, miscarriage, abortion, or current pregnancy. If the total cannot be determined, code 9 = unclear/missing.

3. Number of miscarriages (number_miscarriages)	
**Variable type**	Integer
**Allowed values**	0 = none; 1 = one; 2 = two.
**Missing value**	9 = unclear/missing
**Definition**	Number of pregnancies reported as ending in miscarriage.
**Coding decision rule**	Count only pregnancy losses explicitly described as miscarriage. Do not infer miscarriage from an unexplained difference between pregnancies and children. If unclear, code 9.

4. Current reproductive status (current_reproductive_status)	
**Variable type**	Categorical integer
**Allowed values**	1 = has a child under 2 years; 2 = currently pregnant; 3 = recent abortion; 9 = unclear/other.
**Missing value**	9 = unclear/missing
**Definition**	Participant's reproductive situation at the time of interview.
**Coding decision rule**	Assign according to the participant's current or most immediately relevant reproductive status. Do not infer status unless supported by the interview. If more than one category applies, establish a predetermined priority rule or retain the categories separately rather than choosing arbitrarily.

5. Current family-planning method (current_fp_method)	
**Variable type**	Categorical integer
**Allowed values**	0 = no method; 1 = barrier, including condoms; 2 = natural/withdrawal/traditional; 3 = hormonal; 4 = copper IUD; 5 = mixed/combined methods; 9 = unclear.
**Missing value**	9 = unclear/missing
**Definition**	Family-planning/contraceptive method being used currently.
**Coding decision rule**	Code the method being used at the time of interview. If two or more method categories are being used concurrently, code 5. If the participant explicitly states that no method is being used, code 0. Do not code a past method as current.

6. Marital status (marital_status)	
**Variable type**	Categorical integer
**Allowed values**	1 = married; 2 = single; 9 = unclear/missing.
**Missing value**	9 = unclear/missing
**Definition**	Marital/partnership status reported at interview.
**Coding decision rule**	Code according to explicit participant information. Do not infer marital status from having children, pregnancy, or mentioning a partner.

7. Ever used family-planning method (ever_fp_method)	
**Variable type**	Categorical integer
**Allowed values**	0 = never used a method; 1 = barrier; 2 = natural/withdrawal/traditional; 3 = hormonal; 4 = IUD; 5 = multiple/mixed method categories; 9 = unclear.
**Missing value**	9 = unclear/missing
**Definition**	Whether and what type of family-planning methods the participant has ever used, including current and previous use.
**Coding decision rule**	Code 0 only when there is evidence that the participant has never used contraception. Use 5 when the participant reports experience with methods belonging to more than one category. Current use counts as ever use.

Structural socioeconomic and legal determinants

8. Education level (education_level)	
**Variable type**	Categorical integer
**Allowed values**	1 = Secondary/high school; 2 = Bachelor’s degree; 3 = Master’s degree; 4 = PhD/doctoral degree; 9 = unclear/missing
**Missing value**	9 = unclear/missing
**Definition**	Highest completed education level.
**Coding decision rule**	Code the highest completed qualification; do not infer from occupation or employment.

9. Employment status (employment_status)	
**Variable type**	Categorical integer
**Allowed values**	1 = Employed; 2 = Maternity/parental leave; 3 = Unemployed; 9 = unclear/missing
**Missing value**	9 = unclear/missing
**Definition**	Current employment status.
**Coding decision rule**	Use explicit current status; do not infer from family, income, or education.

10. Legal-status security (legal_status_security)	
**Variable type**	Categorical integer
**Allowed values**	1 = Secure; 2 = Partly secure; 3 = Insecure/uncertain; 9 = unclear/not assessed
**Missing value**	9 = unclear/not assessed
**Definition**	Security of residence or legal status.
**Coding decision rule**	Do not infer from nationality, migrant/refugee background, or years in Germany.

Migration-context and background characteristics

11. Years in Germany (years_germany)	
**Variable type**	Continuous numeric
**Allowed values**	Continuous numeric years; decimals allowed
**Missing value**	Blank = missing
**Definition**	Total duration lived in Germany.
**Coding decision rule**	Use decimal years when appropriate; do not estimate from age or migration reason.

12. Years in Berlin (years_berlin)	
**Variable type**	Continuous numeric
**Allowed values**	Continuous numeric years; decimals allowed
**Missing value**	Blank = missing
**Definition**	Total duration lived in Berlin.
**Coding decision rule**	Do not assume this equals years in Germany unless explicitly supported.

13. Country of origin (country_origin)	
**Variable type**	Text
**Allowed values**	Country name (text)
**Missing value**	Blank = missing/unclear
**Definition**	Participant-reported country of origin.
**Coding decision rule**	Do not infer from language, ethnicity, name, or nationality.

14. Migration reason (migration_reason)	
**Variable type**	Categorical integer
**Allowed values**	1 = Family; 2 = Refuge/protection; 3 = Study; 4 = Work; 9 = unclear/missing
**Missing value**	9 = unclear/missing
**Definition**	Primary reason for moving to Germany.
**Coding decision rule**	Code the main reason at the time of migration; do not infer from current circumstances.

Intermediary material, biological, and linguistic determinants

15. Age (years) (age_years)	
**Variable type**	Continuous numeric
**Allowed values**	Completed years (continuous numeric)
**Missing value**	Blank = missing
**Definition**	Age at interview in completed years.
**Coding decision rule**	Record the reported age; do not categorize.

16. Financial security level (financial_security)	
**Variable type**	Categorical integer
**Allowed values**	1 = Financially insecure; 2 = Moderately secure; 3 = Financially secure; 9 = unclear
**Missing value**	9 = unclear
**Definition**	Overall financial security.
**Coding decision rule**	Classify from direct evidence of ability to meet essential expenses; do not infer from employment or education.

17. German proficiency (german_proficiency)	
**Variable type**	Categorical integer
**Allowed values**	1 = Limited; 2 = Moderate; 3 = Proficient; 9 = unclear
**Missing value**	9 = unclear
**Definition**	German-language proficiency.
**Coding decision rule**	Code from reported ability to speak, understand, read, or use German in healthcare; do not infer from years in Germany.

Unmet Needs

18. Communication & Information (unmet_communication_information)	
**Variable type**	Binary integer
**Allowed values**	0 = not evidenced; 1 = unmet need evidenced
**Missing value**	0 means not evidenced; no separate missing code
**Definition**	There is evidence of an unmet information or communication need: information is missing, unclear, insufficient, poorly timed, difficult to understand, not proactively provided, not available in an appropriate language, or the participant has to seek information elsewhere because formal support is insufficient.
**Coding decision rule**	There is evidence of an unmet information or communication need: information is missing, unclear, insufficient, poorly timed, difficult to understand, not proactively provided, not available in an appropriate language, or the participant has to seek information elsewhere because formal support is insufficient.
**Code 0 / exclusions**	Assign 0 when no information/communication problem is expressed. Exclude simple mentions of receiving information, using the internet/apps, language, or counselling when the participant does not indicate a gap, difficulty, dissatisfaction, or unmet need.
**If unsure**	Ask: Is there evidence that the participant needed better/more accessible information or communication than they received? If yes -> 1. If this cannot be established -> 0.

19. Continuity & Affordability (unmet_continuity_affordability)	
**Variable type**	Binary integer
**Allowed values**	0 = not evidenced; 1 = unmet need evidenced
**Missing value**	0 means not evidenced; no separate missing code
**Definition**	There is evidence that cost or a practical/system barrier makes obtaining or continuing contraception difficult, including expense, prescriptions, repeated appointments, administrative requirements, availability, time, travel, caregiving, or healthcare-navigation difficulties.
**Coding decision rule**	There is evidence that cost or a practical/system barrier makes obtaining or continuing contraception difficult, including expense, prescriptions, repeated appointments, administrative requirements, availability, time, travel, caregiving, or healthcare-navigation difficulties.
**Code 0 / exclusions**	Assign 0 when no access/continuity difficulty is expressed. Exclude mentions of paying, appointments, prescriptions, travel, etc. when they are described neutrally and do not create a burden or barrier.
**If unsure**	Ask: Does this issue make contraception harder to obtain, afford, or continue? Yes -> 1. No/unclear -> 0.

20. Respect & Shared Responsibility (unmet_respect_shared_responsibility)	
**Variable type**	Binary integer
**Allowed values**	0 = not evidenced; 1 = unmet need evidenced
**Missing value**	0 means not evidenced; no separate missing code
**Definition**	There is evidence of an unmet need for respect, autonomy, shared decision-making, individualised care, or equitable contraceptive responsibility, such as pressure to use a method, preferences not being respected, not being listened to, one-sided decision-making, or unequal gender/partner burden.
**Coding decision rule**	There is evidence of an unmet need for respect, autonomy, shared decision-making, individualised care, or equitable contraceptive responsibility, such as pressure to use a method, preferences not being respected, not being listened to, one-sided decision-making, or unequal gender/partner burden.
**Code 0 / exclusions**	Assign 0 when no problem with respect, autonomy, decision-making, or responsibility is expressed. Exclude statements about personal preferences, method choices, or gender differences alone unless they indicate dissatisfaction, unfairness, pressure, or a need for change.
**If unsure**	Ask: Is the participant identifying something about respect, decision-making, or responsibility that should have been different? Yes -> 1. If not evident -> 0.

21. Cultural & Life-Course Responsiveness (unmet_cultural_lifecourse_responsiveness)	
**Variable type**	Binary integer
**Allowed values**	0 = not evidenced; 1 = unmet need evidenced
**Missing value**	0 means not evidenced; no separate missing code
**Definition**	There is evidence that family-planning care should better respond to the participant's cultural/migration context, previous healthcare experiences, or current/changing reproductive circumstances, such as postpartum status, breastfeeding, age, fertility intentions, or changing contraceptive needs.
**Coding decision rule**	There is evidence that family-planning care should better respond to the participant's cultural/migration context, previous healthcare experiences, or current/changing reproductive circumstances, such as postpartum status, breastfeeding, age, fertility intentions, or changing contraceptive needs.
**Code 0 / exclusions**	Assign 0 when these characteristics or circumstances are merely mentioned without an unmet need. Exclude being a migrant, mentioning culture, breastfeeding, pregnancy/postpartum, age, or previous healthcare experiences by itself.
**If unsure**	Ask: Does the participant indicate that care/information should be adapted because of this context or life stage? Yes -> 1. If the context is only descriptive -> 0.

Psychological Pathways

22. Trauma-related fear concerning reproduction (trauma_fear_reproduction)	
**Pathway role**	Barrier
**Variable type**	Binary integer
**Allowed values**	0 = not evidenced; 1 = evidenced
**Missing value**	0 means not evidenced; no separate missing code
**Definition**	Past trauma, violence, reproductive loss, displacement, FGM/cutting, or another harmful experience generates fear, anxiety, uncertainty, or concern about pregnancy, childbirth, contraception, or reproductive healthcare. Based on PSY-1 Trauma and reproductive fear.
**Coding decision rule**	Past trauma, violence, reproductive loss, displacement, FGM/cutting, or another harmful experience generates fear, anxiety, uncertainty, or concern about pregnancy, childbirth, contraception, or reproductive healthcare. Based on PSY-1 Trauma and reproductive fear.
**Code 0 / exclusions**	Trauma or a difficult past experience is mentioned but no link is made to current reproductive fear, pregnancy, contraception, childbirth, or reproductive care. Also exclude general reproductive worry with no trauma-related basis.
**If unsure**	Ask: Is a past harmful experience contributing to reproductive fear now? Yes -> 1. Cannot establish the link -> 0.

23. Unaddressed trauma and psychological distress (unaddressed_trauma_distress)	
**Pathway role**	Barrier
**Variable type**	Binary integer
**Allowed values**	0 = not evidenced; 1 = evidenced
**Missing value**	0 means not evidenced; no separate missing code
**Definition**	Participant describes ongoing or unresolved emotional pain, grief, fear, distress, painful memories, or trauma-related experiences that have not been adequately acknowledged, explored, or addressed. Based mainly on PSY-2 and the unmet-recognition aspect of PSY-3.
**Coding decision rule**	Participant describes ongoing or unresolved emotional pain, grief, fear, distress, painful memories, or trauma-related experiences that have not been adequately acknowledged, explored, or addressed. Based mainly on PSY-2 and the unmet-recognition aspect of PSY-3.
**Code 0 / exclusions**	Past trauma is mentioned without current emotional distress, or emotional needs were appropriately recognized and addressed rather than remaining unaddressed.
**If unsure**	Look for evidence of both distress/pain and that it remains unresolved, unspoken, overlooked, or insufficiently addressed. If this cannot be established -> 0.

24. Pre-existing or newly developed mental-health concerns (mental_health_concerns)	
**Pathway role**	Barrier
**Variable type**	Binary integer
**Allowed values**	0 = not evidenced; 1 = evidenced
**Missing value**	0 means not evidenced; no separate missing code
**Definition**	Participant reports anxiety, emotional distress, grief, fear, psychological pain, or other emotional/mental-health difficulties relevant to reproductive health, migration experiences, or healthcare engagement. Based on PSY-2 Emotional and psychological distress.
**Coding decision rule**	Participant reports anxiety, emotional distress, grief, fear, psychological pain, or other emotional/mental-health difficulties relevant to reproductive health, migration experiences, or healthcare engagement. Based on PSY-2 Emotional and psychological distress.
**Code 0 / exclusions**	Ordinary worry, dissatisfaction, or stress is mentioned without evidence of meaningful psychological/emotional distress. Do not code merely because mental health is discussed generally.
**If unsure**	Ask: Is the participant describing her own meaningful emotional or psychological difficulty? Yes -> 1. Ambiguous -> 0.

25. Provider compassion and empathy (provider_compassion_empathy)	
**Pathway role**	Facilitator
**Variable type**	Binary integer
**Allowed values**	0 = not evidenced; 1 = evidenced
**Missing value**	0 means not evidenced; no separate missing code
**Definition**	A provider demonstrates empathy, listening, validation, sensitivity, understanding, or compassionate communication, contributing to trust, comfort, safety, or willingness to communicate. Based on PSY-4 Psychological safety and empathetic care.
**Coding decision rule**	A provider demonstrates empathy, listening, validation, sensitivity, understanding, or compassionate communication, contributing to trust, comfort, safety, or willingness to communicate. Based on PSY-4 Psychological safety and empathetic care.
**Code 0 / exclusions**	Provider is described as clinically competent, available, or generally “good” without evidence of empathy, listening, validation, or emotional sensitivity.
**If unsure**	Look for a specific compassionate interaction or its positive effect. Absence of a negative experience alone is not enough -> 0.

26. Directly addressing trauma / trauma-informed emotional support (trauma_informed_support)	
**Pathway role**	Facilitator
**Variable type**	Binary integer
**Allowed values**	0 = not evidenced; 1 = evidenced
**Missing value**	0 means not evidenced; no separate missing code
**Definition**	A provider or service recognizes trauma/emotional needs and responds sensitively, for example by creating psychological safety, avoiding harmful interaction, listening to painful experiences, validating feelings, or adapting care to trauma. Based on PSY-3 and PSY-4.
**Coding decision rule**	A provider or service recognizes trauma/emotional needs and responds sensitively, for example by creating psychological safety, avoiding harmful interaction, listening to painful experiences, validating feelings, or adapting care to trauma. Based on PSY-3 and PSY-4.
**Code 0 / exclusions**	General kindness or good communication without evidence that care was responsive to trauma or emotional vulnerability.
**If unsure**	Ask: Was the support specifically responsive to trauma, distress, or emotional safety? Yes -> 1. General empathy only -> code provider_compassion_empathy = 1, but this variable 0.

Individual Pathways

27. Perception of contraceptive side effects (perceived_contraceptive_side_effects)	
**Pathway role**	Barrier
**Variable type**	Binary integer
**Allowed values**	0 = not evidenced; 1 = evidenced
**Missing value**	0 means not evidenced; no separate missing code
**Definition**	Concerns, expectations, fears, or experiences of contraceptive side effects shape method preference, avoidance, discontinuation, refusal, or another reproductive decision. Based on IND-1 Contraceptive knowledge and perceptions.
**Coding decision rule**	Concerns, expectations, fears, or experiences of contraceptive side effects shape method preference, avoidance, discontinuation, refusal, or another reproductive decision. Based on IND-1 Contraceptive knowledge and perceptions.
**Code 0 / exclusions**	Side effects are merely mentioned or known about but do not influence a contraceptive preference or decision.
**If unsure**	Ask: Did perceived/experienced side effects affect what she chose or did? Yes -> 1. No clear influence -> 0.

28. Negative perceptions or misinformation about contraception (contraceptive_misinformation)	
**Pathway role**	Barrier
**Variable type**	Binary integer
**Allowed values**	0 = not evidenced; 1 = evidenced
**Missing value**	0 means not evidenced; no separate missing code
**Definition**	Misleading, inaccurate, unverified, or fear-based contraceptive information/beliefs-such as “horror stories”-shape contraceptive perceptions, preferences, or decisions. Based on IND-1.
**Coding decision rule**	Misleading, inaccurate, unverified, or fear-based contraceptive information/beliefs-such as “horror stories”-shape contraceptive perceptions, preferences, or decisions. Based on IND-1.
**Code 0 / exclusions**	Participant accurately discusses risks or side effects, or reports a concern without evidence that misinformation is involved. Do not automatically label a belief misinformation simply because it differs from a provider's recommendation.
**If unsure**	If you cannot confidently determine that the information is misinformation from the qualitative material, do not infer it -> 0. The broader perception may still be represented elsewhere.

29. Limited health literacy (limited_health_literacy)	
**Pathway role**	Barrier
**Variable type**	Binary integer
**Allowed values**	0 = not evidenced; 1 = evidenced
**Missing value**	0 means not evidenced; no separate missing code
**Definition**	Difficulty understanding reproductive-health information, terminology, healthcare instructions, contraceptive information, or how to use/navigate healthcare information limits understanding or access. Based on IND-2 Health literacy and language.
**Coding decision rule**	Difficulty understanding reproductive-health information, terminology, healthcare instructions, contraceptive information, or how to use/navigate healthcare information limits understanding or access. Based on IND-2 Health literacy and language.
**Code 0 / exclusions**	Simply having lower German proficiency or needing translation does not automatically equal limited health literacy. Also exclude lack of information caused by providers when the problem is not the participant's ability to understand it.
**If unsure**	Ask: Is the problem her ability to understand/use health information? Yes -> 1. If the issue is only language proficiency without evidence of health-information difficulty -> 0 here.

30. Active information seeking (active_information_seeking)	
**Pathway role**	Facilitator
**Variable type**	Binary integer
**Allowed values**	0 = not evidenced; 1 = evidenced
**Missing value**	0 means not evidenced; no separate missing code
**Definition**	Participant independently searches, researches, compares, checks, verifies, or develops information about contraception, pregnancy, reproductive health, or healthcare services. Based on IND-3 Information seeking and knowledge development.
**Coding decision rule**	Participant independently searches, researches, compares, checks, verifies, or develops information about contraception, pregnancy, reproductive health, or healthcare services. Based on IND-3 Information seeking and knowledge development.
**Code 0 / exclusions**	Participant passively receives information from a doctor, friend, NGO, etc., without independently seeking or checking information.
**If unsure**	Look for an active verb/mechanism: searched, researched, checked, compared, looked up, asked around. Merely possessing knowledge -> 0.

31. Decision-making confidence (decision_making_confidence)	
**Pathway role**	Facilitator
**Variable type**	Binary integer
**Allowed values**	0 = not evidenced; 1 = evidenced
**Missing value**	0 means not evidenced; no separate missing code
**Definition**	Participant expresses confidence in knowing her reproductive preferences, evaluating options, or making contraceptive/reproductive decisions. Based on IND-4 Reproductive agency and self-advocacy.
**Coding decision rule**	Participant expresses confidence in knowing her reproductive preferences, evaluating options, or making contraceptive/reproductive decisions. Based on IND-4 Reproductive agency and self-advocacy.
**Code 0 / exclusions**	Participant makes a decision but does not demonstrate confidence, certainty, or perceived ability to make the decision.
**If unsure**	Ask: Does she communicate confidence/agency in deciding what she wants? Yes -> 1. Decision alone -> 0.

32. Self-advocacy (self_advocacy)	
**Pathway role**	Facilitator
**Variable type**	Binary integer
**Allowed values**	0 = not evidenced; 1 = evidenced
**Missing value**	0 means not evidenced; no separate missing code
**Definition**	Participant acts to communicate or defend her needs/preferences, including asking questions, challenging recommendations, refusing unwanted treatment, seeking another opinion, changing providers, requesting care, or asserting her rights/preferences. Based on IND-4.
**Coding decision rule**	Participant acts to communicate or defend her needs/preferences, including asking questions, challenging recommendations, refusing unwanted treatment, seeking another opinion, changing providers, requesting care, or asserting her rights/preferences. Based on IND-4.
**Code 0 / exclusions**	Participant has preferences or confidence but does not take an advocacy action. Do not code merely because she disagrees internally with a provider.
**If unsure**	Ask: Did she actively speak up, request, challenge, refuse, seek alternatives, or otherwise advocate for herself? Yes -> 1. Confidence without action may be decision_making_confidence = 1 but self_advocacy = 0.

Systems of Power

33. Racism, discriminatory treatment, and racial othering (racism_discrimination_othering)	
**Pathway role**	Barrier
**Variable type**	Binary integer
**Allowed values**	0 = not evidenced; 1 = evidenced
**Missing value**	0 means not evidenced; no separate missing code
**Definition**	Participant experiences or describes racism, discrimination, racialization, unequal treatment, or being treated as an outsider/“other” because of race, ethnicity, skin colour, migration status, or national background. Based on SYS-1.
**Coding decision rule**	Participant experiences or describes racism, discrimination, racialization, unequal treatment, or being treated as an outsider/“other” because of race, ethnicity, skin colour, migration status, or national background. Based on SYS-1.
**Code 0 / exclusions**	Poor treatment, disagreement, rudeness, or an unsatisfactory healthcare encounter without evidence connecting it to race, ethnicity, migration background, nationality, or another relevant identity.
**If unsure**	Do not infer discrimination from poor care alone. There must be an explicit or clearly supported identity-related dimension. Otherwise -> 0.

34. Provider stereotyping and lack of cultural preparedness (provider_stereotyping)	
**Pathway role**	Barrier
**Variable type**	Binary integer
**Allowed values**	0 = not evidenced; 1 = evidenced
**Missing value**	0 means not evidenced; no separate missing code
**Definition**	A healthcare provider makes assumptions, generalizations, judgments, or stereotyped expectations about the participant based on race, ethnicity, nationality, migration status, culture, or related characteristics. Based on SYS-1.
**Coding decision rule**	A healthcare provider makes assumptions, generalizations, judgments, or stereotyped expectations about the participant based on race, ethnicity, nationality, migration status, culture, or related characteristics. Based on SYS-1.
**Code 0 / exclusions**	Provider gives advice the participant dislikes, makes a clinical recommendation, or communicates poorly without evidence of stereotyping.
**If unsure**	Ask: Is the provider treating her according to an assumed group characteristic rather than her individual circumstances? Yes -> 1. Unclear -> 0.

35. Structural racism within healthcare systems (structural_racism_healthcare)	
**Pathway role**	Barrier
**Variable type**	Binary integer
**Allowed values**	0 = not evidenced; 1 = evidenced
**Missing value**	0 means not evidenced; no separate missing code
**Definition**	Participant describes racism or racialized inequality as embedded in healthcare institutions, routine practices, systems, patterns, or broader institutional organization, rather than only a single interpersonal event. Based on the structural dimension of SYS-1.
**Coding decision rule**	Participant describes racism or racialized inequality as embedded in healthcare institutions, routine practices, systems, patterns, or broader institutional organization, rather than only a single interpersonal event. Based on the structural dimension of SYS-1.
**Code 0 / exclusions**	A single discriminatory interaction is described without indicating a broader institutional/systemic pattern. That may still be racism_discrimination_othering = 1.
**If unsure**	Look for language or context indicating systemic, institutional, repeated, patterned, or structural inequality. If only an individual encounter is supported -> 0 here.

36. Uncertainty about healthcare rights and entitlements (uncertainty_healthcare_rights)	
**Pathway role**	Barrier
**Variable type**	Binary integer
**Allowed values**	0 = not evidenced; 1 = evidenced
**Missing value**	0 means not evidenced; no separate missing code
**Definition**	Participant is uncertain about healthcare entitlements, eligibility, insurance rights, which services she can access, or what she is legally/institutionally entitled to receive. Based on SYS-2 Institutional and administrative access.
**Coding decision rule**	Participant is uncertain about healthcare entitlements, eligibility, insurance rights, which services she can access, or what she is legally/institutionally entitled to receive. Based on SYS-2 Institutional and administrative access.
**Code 0 / exclusions**	General uncertainty about where a clinic is, what contraceptive method to choose, or medical information without uncertainty regarding rights/eligibility/entitlements.
**If unsure**	Ask: Is the uncertainty about what the healthcare system allows or entitles her to receive? Yes -> 1. Otherwise -> 0.

37. Financial barriers (financial_barriers)	
**Pathway role**	Barrier
**Variable type**	Binary integer
**Allowed values**	0 = not evidenced; 1 = evidenced
**Missing value**	0 means not evidenced; no separate missing code
**Definition**	Costs, insufficient income, inability to pay, insurance gaps, or financial strain restrict or complicate contraception, reproductive-health care, treatment, transport, or service use. Based on SYS-3 Financial and material conditions.
**Coding decision rule**	Costs, insufficient income, inability to pay, insurance gaps, or financial strain restrict or complicate contraception, reproductive-health care, treatment, transport, or service use. Based on SYS-3 Financial and material conditions.
**Code 0 / exclusions**	A cost is mentioned but it does not create difficulty or influence access/use. Low income alone is not enough unless it affects reproductive-health access or care.
**If unsure**	Ask: Did money make obtaining or using care more difficult? Yes -> 1. Cost without demonstrated barrier -> 0.

38. Housing and material insecurity (housing_material_insecurity)	
**Pathway role**	Barrier
**Variable type**	Binary integer
**Allowed values**	0 = not evidenced; 1 = evidenced
**Missing value**	0 means not evidenced; no separate missing code
**Definition**	Housing instability, food insecurity, transport difficulties, lack of essential material resources, or similar material circumstances affect reproductive health, pregnancy experience, contraceptive access, or healthcare use. Based on SYS-3.
**Coding decision rule**	Housing instability, food insecurity, transport difficulties, lack of essential material resources, or similar material circumstances affect reproductive health, pregnancy experience, contraceptive access, or healthcare use. Based on SYS-3.
**Code 0 / exclusions**	Participant mentions housing, transport, or material circumstances without showing that they influence reproductive-health experience/access.
**If unsure**	Require a clear or strongly implied connection to reproductive health or healthcare. If not established -> 0.

39. Insurance and healthcare coverage (insurance_healthcare_coverage)	
**Pathway role**	Facilitator
**Variable type**	Binary integer
**Allowed values**	0 = not evidenced; 1 = evidenced
**Missing value**	0 means not evidenced; no separate missing code
**Definition**	Insurance or another healthcare-coverage arrangement enables access, eligibility, payment, or use of reproductive-health services. This is the facilitating side of SYS-2.
**Coding decision rule**	Insurance or another healthcare-coverage arrangement enables access, eligibility, payment, or use of reproductive-health services. This is the facilitating side of SYS-2.
**Code 0 / exclusions**	Merely having health insurance is mentioned without evidence that it enables or facilitates access. Do not code 1 simply because no insurance problem is reported.
**If unsure**	Ask: Is coverage functioning as a resource that helps her obtain care? Yes -> 1. Mere insurance status -> 0.

40. Knowledge of healthcare entitlements (knowledge_healthcare_entitlements)	
**Pathway role**	Facilitator
**Variable type**	Binary integer
**Allowed values**	0 = not evidenced; 1 = evidenced
**Missing value**	0 means not evidenced; no separate missing code
**Definition**	Participant knows or understands what healthcare services, benefits, or reproductive-health services she is entitled to, and this knowledge supports access or navigation. Derived from the facilitating counterpart of SYS-2.
**Coding decision rule**	Participant knows or understands what healthcare services, benefits, or reproductive-health services she is entitled to, and this knowledge supports access or navigation. Derived from the facilitating counterpart of SYS-2.
**Code 0 / exclusions**	General healthcare knowledge, contraceptive knowledge, or knowing where a clinic is without knowledge specifically about rights, eligibility, or entitlements.
**If unsure**	Ask: Does she know what the system allows her to access/receive? Yes -> 1. General health knowledge -> 0.

41. Ability to manage healthcare costs (ability_manage_healthcare_costs)	
**Pathway role**	Facilitator
**Variable type**	Binary integer
**Allowed values**	0 = not evidenced; 1 = evidenced
**Missing value**	0 means not evidenced; no separate missing code
**Definition**	Participant has sufficient financial/material capacity to pay for or manage healthcare/contraceptive costs without those costs preventing access, and this operates as a resource. Facilitating counterpart of SYS-3.
**Coding decision rule**	Participant has sufficient financial/material capacity to pay for or manage healthcare/contraceptive costs without those costs preventing access, and this operates as a resource. Facilitating counterpart of SYS-3.
**Code 0 / exclusions**	No financial barrier is reported, but there is also no evidence that ability to manage costs is functioning as a facilitator. Absence of hardship alone is not enough.
**If unsure**	Look for positive evidence such as being able to afford, budget for, reimburse, or otherwise manage costs. If this is only assumed -> 0.

Relationships and Networks

42. Social isolation (social_isolation)	
**Pathway role**	Barrier
**Variable type**	Binary integer
**Allowed values**	0 = not evidenced; 1 = evidenced
**Missing value**	0 means not evidenced; no separate missing code
**Definition**	Participant describes absence or insufficiency of family, friends, relatives, peers, or trusted social connections, and this affects emotional wellbeing, reproductive decisions, or healthcare access. Based on NET-1 Family and social support.
**Coding decision rule**	Participant describes absence or insufficiency of family, friends, relatives, peers, or trusted social connections, and this affects emotional wellbeing, reproductive decisions, or healthcare access. Based on NET-1 Family and social support.
**Code 0 / exclusions**	Living alone, being a migrant, or having family abroad without evidence that lack of social connection creates a problem.
**If unsure**	Ask: Is lack of social connection itself operating as a barrier? Yes -> 1. Merely having a small network -> 0.

43. Lack of practical support (lack_practical_support)	
**Pathway role**	Barrier
**Variable type**	Binary integer
**Allowed values**	0 = not evidenced; 1 = evidenced
**Missing value**	0 means not evidenced; no separate missing code
**Definition**	Participant lacks concrete assistance needed for interpretation, transport, childcare, appointments, communication, locating services, accompaniment, or healthcare navigation. Based mainly on the absence of NET-2 Practical and navigation support.
**Coding decision rule**	Participant lacks concrete assistance needed for interpretation, transport, childcare, appointments, communication, locating services, accompaniment, or healthcare navigation. Based mainly on the absence of NET-2 Practical and navigation support.
**Code 0 / exclusions**	General loneliness or lack of emotional support with no practical assistance needed.
**If unsure**	Ask: Was specific practical help needed but unavailable? Yes -> 1. Emotional isolation only -> consider social_isolation, not this variable.

44. Childcare burden or interpersonal stigma (childcare_burden_stigma)	
**Pathway role**	Barrier
**Variable type**	Binary integer
**Allowed values**	0 = not evidenced; 1 = evidenced
**Missing value**	0 means not evidenced; no separate missing code
**Definition**	Code 1 when either childcare responsibilities/lack of childcare materially constrain healthcare access OR interpersonal stigma/social pressure from family, peers, or community constrains reproductive decisions or care. These mechanisms derive from NET-1/NET-2.
**Coding decision rule**	Code 1 when either childcare responsibilities/lack of childcare materially constrain healthcare access OR interpersonal stigma/social pressure from family, peers, or community constrains reproductive decisions or care. These mechanisms derive from NET-1/NET-2.
**Code 0 / exclusions**	Having children alone is not a barrier. Mentioning social attitudes without experiencing pressure, stigma, or constraint is also not sufficient.
**If unsure**	Important: this variable combines two conceptually different mechanisms. If you retain it as one binary variable, code 1 if either childcare burden or interpersonal stigma is evidenced. If possible analytically, splitting it into childcare_burden and interpersonal_stigma would align better with the qualitative codebook.

45. NGO and community support (ngo_community_support)	
**Pathway role**	Facilitator
**Variable type**	Binary integer
**Allowed values**	0 = not evidenced; 1 = evidenced
**Missing value**	0 means not evidenced; no separate missing code
**Definition**	NGO, refugee organization, community organization, advocate, or community worker provides information, referrals, advocacy, accompaniment, or access support. Based on NET-3 Community and organizational support.
**Coding decision rule**	NGO, refugee organization, community organization, advocate, or community worker provides information, referrals, advocacy, accompaniment, or access support. Based on NET-3 Community and organizational support.
**Code 0 / exclusions**	Participant simply knows that an NGO exists or attends an organization without evidence that it provides relevant support.
**If unsure**	Ask: Did an organized community/civil-society source actually support reproductive-health knowledge or access? Yes -> 1. Otherwise -> 0.

46. Peer support and knowledge sharing (peer_support_knowledge_sharing)	
**Pathway role**	Facilitator
**Variable type**	Binary integer
**Allowed values**	0 = not evidenced; 1 = evidenced
**Missing value**	0 means not evidenced; no separate missing code
**Definition**	Friends, peers, other women, or social groups provide or exchange experiences, advice, reproductive-health information, recommendations, or emotional support. Based on NET-4 Peer and digital knowledge networks.
**Coding decision rule**	Friends, peers, other women, or social groups provide or exchange experiences, advice, reproductive-health information, recommendations, or emotional support. Based on NET-4 Peer and digital knowledge networks.
**Code 0 / exclusions**	Information comes solely from healthcare providers, NGOs, or the participant's own independent research.
**If unsure**	Look for another peer/person-to-person social source sharing knowledge/support. If absent -> 0.

47. Digital support networks (digital_support_networks)	
**Pathway role**	Facilitator
**Variable type**	Binary integer
**Allowed values**	0 = not evidenced; 1 = evidenced
**Missing value**	0 means not evidenced; no separate missing code
**Definition**	Online communities, messaging groups, social media, or similar digital networks provide social support, shared experiences, advice, or reproductive-health information. Based on the digital component of NET-4.
**Coding decision rule**	Online communities, messaging groups, social media, or similar digital networks provide social support, shared experiences, advice, or reproductive-health information. Based on the digital component of NET-4.
**Code 0 / exclusions**	Participant independently searches Google/websites/apps without interacting with or receiving support from a network/community. That is better coded active_information_seeking.
**If unsure**	Ask: Is the digital mechanism a social/network interaction rather than solitary information searching? Network/community -> 1. Individual searching -> 0.

48. Navigation support (navigation_support)	
**Pathway role**	Facilitator
**Variable type**	Binary integer
**Allowed values**	0 = not evidenced; 1 = evidenced
**Missing value**	0 means not evidenced; no separate missing code
**Definition**	Another person provides concrete help navigating healthcare, such as interpretation, locating services, making appointments, communicating with providers, accompaniment, referrals, or explaining how to access care. Based on NET-2.
**Coding decision rule**	Another person provides concrete help navigating healthcare, such as interpretation, locating services, making appointments, communicating with providers, accompaniment, referrals, or explaining how to access care. Based on NET-2.
**Code 0 / exclusions**	Participant navigates the system herself, receives only emotional encouragement, or independently researches services.
**If unsure**	Ask: Did another person actively help her enter, understand, or move through the healthcare system? Yes -> 1. Otherwise -> 0.

3. Value Labels

The following table consolidates the controlled categorical values used in the accompanying empty data shell.

Variable	Code	Value label	Notes
all_binary	0	Not evidenced	All unmet-need and pathway variables
all_binary	1	Evidenced	All unmet-need and pathway variables
number_children	0-3	Reported number of living children	9 = unclear/missing
total_pregnancies	0-4	Reported total pregnancies	9 = unclear/missing
number_miscarriages	0-2	Reported miscarriages	9 = unclear/missing
current_reproductive_status	1	Has a child under 2 years	
current_reproductive_status	2	Currently pregnant	
current_reproductive_status	3	Recent abortion	
current_reproductive_status	9	Unclear/other	
current_fp_method	0	No current method	
current_fp_method	1	Barrier method, including condoms	
current_fp_method	2	Natural/withdrawal/traditional	
current_fp_method	3	Hormonal	
current_fp_method	4	Copper IUD	
current_fp_method	5	Mixed/combined method categories	
current_fp_method	9	Unclear	
ever_fp_method	0	Never used a method	
ever_fp_method	1	Barrier	
ever_fp_method	2	Natural/withdrawal/traditional	
ever_fp_method	3	Hormonal	
ever_fp_method	4	IUD	
ever_fp_method	5	Multiple/mixed method categories	
ever_fp_method	9	Unclear	
marital_status	1	Married	
marital_status	2	Single	
marital_status	9	Unclear/missing	
education_level	1	Secondary/high school	
education_level	2	Bachelor’s degree	
education_level	3	Master’s degree	
education_level	4	PhD/doctoral degree	
education_level	9	Unclear/missing	
employment_status	1	Employed	
employment_status	2	Maternity/parental leave	
employment_status	3	Unemployed	
employment_status	9	Unclear/missing	
legal_status_security	1	Secure legal status	
legal_status_security	2	Partly secure legal status	

Variable	Code	Value label	Notes
legal_status_security	3	Insecure/uncertain legal status	
legal_status_security	9	Unclear/not assessed	
financial_security	1	Financially insecure	
financial_security	2	Moderately financially secure	
financial_security	3	Financially secure	
financial_security	9	Unclear/insufficient information	
german_proficiency	1	Limited proficiency	
german_proficiency	2	Moderate proficiency	
german_proficiency	3	Proficient	
german_proficiency	9	Unclear/insufficient information	
migration_reason	1	Family purposes	
migration_reason	2	Seeking refuge or protection	
migration_reason	3	Study	
migration_reason	4	Work	
migration_reason	9	Unclear/missing	
