# Supplementary material for: “Do not leave me behind and do not put me in your box”: family-planning access and care needs among women with migration histories in Berlin, Germany—a mixed-methods study
Source: Reprod Health. 2026 Sep 25;23:175. doi: 10.1186/s12978-026-02450-6 (PMC13613642; doi:10.1186/s12978-026-02450-6)
Supplement: Supplementary file 1 [file 12978_2026_2450_MOESM1_ESM.zip › Supplementary file 5 _ Family_Planning_Analysis_Report.pdf]

ANALYTICAL REPORT

Family-Planning Care Needs, Pathway Experiences, and Participant-Level Patterns

Counts, cross-tabulations, co-occurrence, Spearman correlations, and bias-corrected Cramér’s V

Participant-level matrix: 29 participants

Prepared from clean_data.xlsx and the existing analysis notebook

Report overview

This report consolidates seven complementary analyses of the transformed participant-by-code matrix. Each section begins with the method, presents the principal tables and figures, and ends with an interpretation. Detailed indicator-level, participant-level, and cross-tabulation tables are retained in appendices so the main report remains readable.

Contents

1. Family-planning care-needs counts and percentages
2. Barrier and facilitator counts and percentages
3. Participant-level barrier and facilitator counts
4. CT: Cross-tabulation analysis
5. COA: Co-occurrence analysis
6. SC: Spearman correlation analysis
7. CV: Bias-corrected Cramér’s V analysis
8. Overall conclusion

Appendices: complete indicator, participant, and cross-tabulation tables

Interpretive boundary

The analyses are exploratory and sample-specific. A code of 0 means that an experience was not identified in the coded material, not necessarily that it was confirmed absent. Counts, co-occurrence, and associations do not establish causation, mediation, prediction, or population prevalence.

1. Family-planning care-needs counts and percentages

Methodology

Four binary care-need indicators were summarized by counting participants coded 1 and dividing by the 29 valid participant records. Percentages were calculated as identified count divided by valid n multiplied by 100.

Table 1. *Family-planning care needs identified in the participant matrix*

Care-need domain	Count	Percentage
Communication and information	28/29	96.6%
Cultural and life-course responsiveness	26/29	89.7%
Continuity and affordability	24/29	82.8%
Respect and shared responsibility	22/29	75.9%

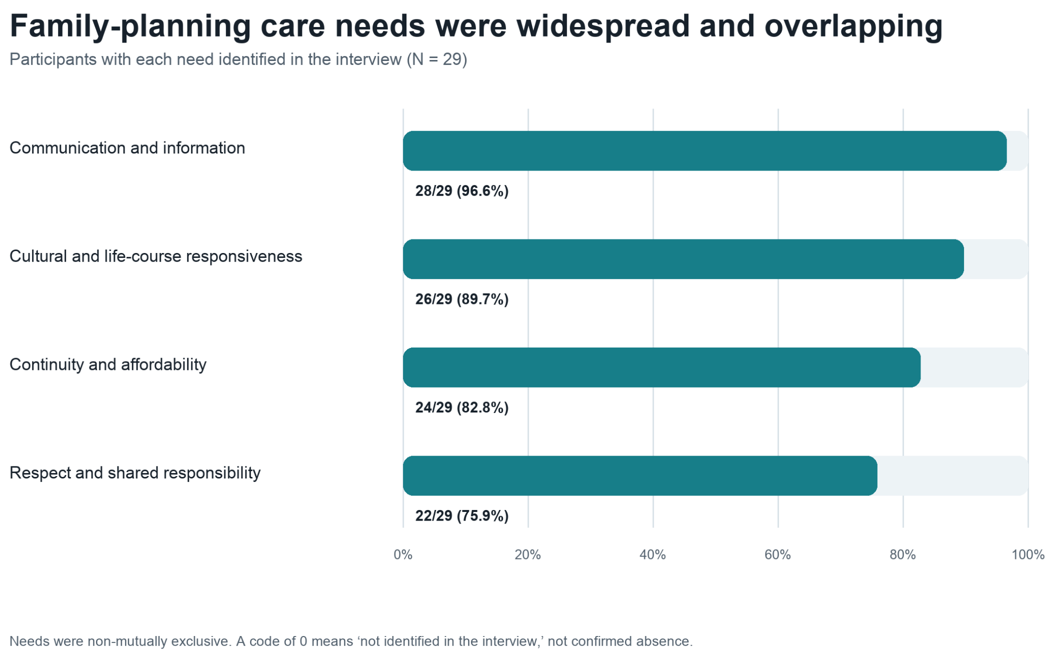

Figure 1. *Counts and percentages for the four family-planning care-need domains.*

Interpretation

All four care needs were common. Communication/information was nearly universal, and cultural/life-course responsiveness, continuity/affordability, and respect/shared responsibility were also identified in most accounts. The high prevalence means that subgroup contrasts involving these outcomes can be unstable, especially for communication/information, where only one participant was coded 0.

2. Barrier and facilitator counts and percentages

Methodology

The 27 binary pathway indicators—15 barriers and 12 facilitators—were summarized across participants. For each indicator, the identified count was divided by its valid participant denominator. All indicators had 29 valid observations.

Table 2. *Most frequently identified barriers and facilitators*

Role	Indicator	Pathway	Count	%
Barrier	Mental-health concerns	Psychological Pathways	23/29	79.3%
Barrier	Childcare burden/stigma	Relationships and Networks	22/29	75.9%
Barrier	Social isolation	Relationships and Networks	19/29	65.5%
Barrier	Provider stereotyping	Systems of Power	16/29	55.2%
Barrier	Structural racism in healthcare	Systems of Power	16/29	55.2%
Barrier	Lack of practical support	Relationships and Networks	16/29	55.2%
Facilitator	Provider compassion/empathy	Psychological Pathways	28/29	96.6%
Facilitator	Self-advocacy	Individual Pathways	25/29	86.2%
Facilitator	Decision-making confidence	Individual Pathways	24/29	82.8%
Facilitator	Navigation support	Relationships and Networks	24/29	82.8%
Facilitator	Peer support/knowledge sharing	Relationships and Networks	21/29	72.4%
Facilitator	Active information seeking	Individual Pathways	20/29	69.0%

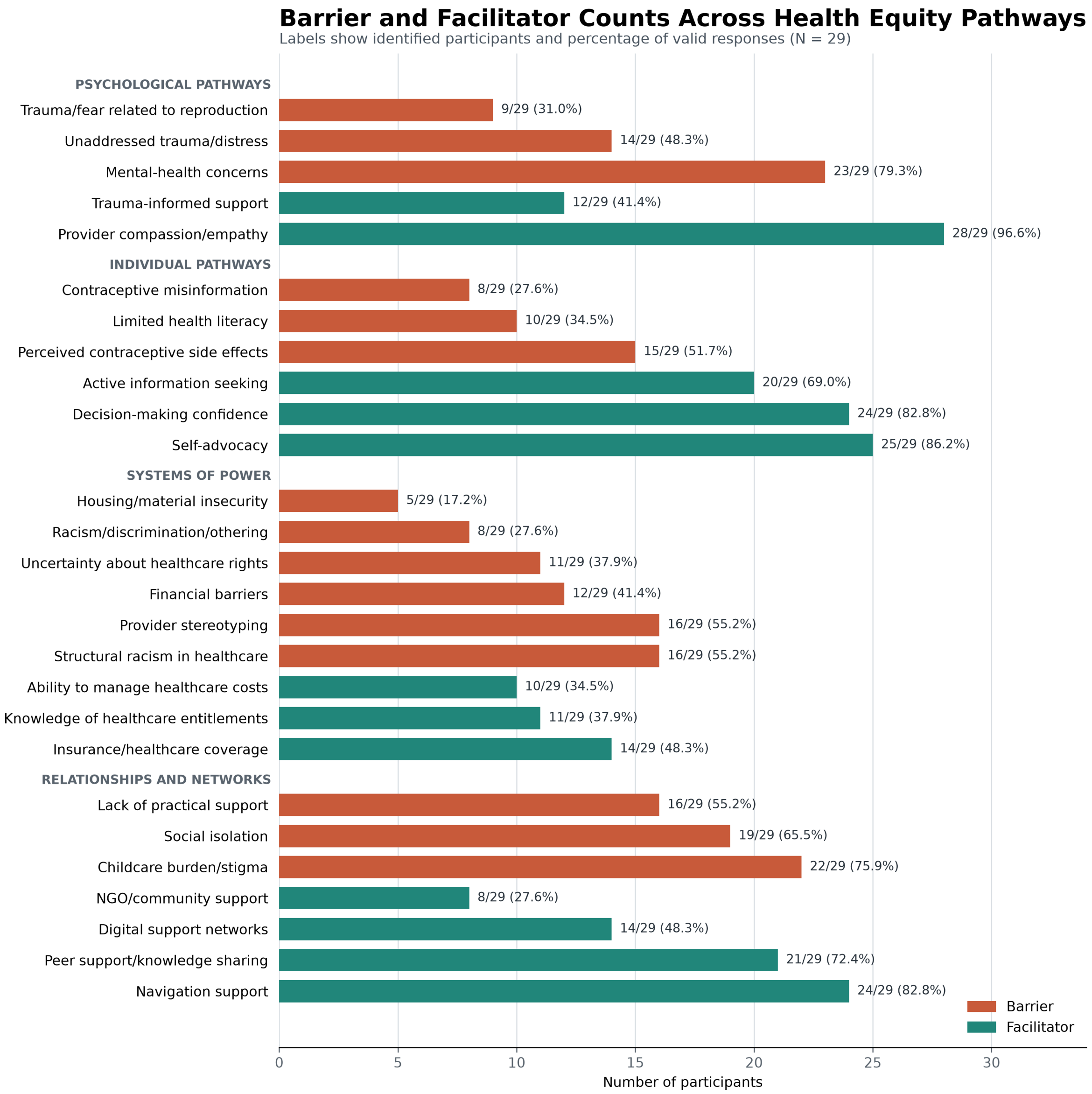

Figure 2. *All pathway-indicator counts and percentages, grouped by Health Equity Framework pathway.*

Interpretation

Provider compassion/empathy was the most frequent facilitator (28/29), followed by self-advocacy (25/29), decision-making confidence (24/29), and navigation support (24/29). The most common barriers were mental-health concerns (23/29), childcare burden/stigma (22/29), and social isolation (19/29). The sample therefore showed substantial barriers alongside strong agency and supportive resources.

3. Participant-level barrier and facilitator counts

Methodology

For each participant, the 15 barrier indicators and 12 facilitator indicators were summed separately. Participant percentages used the relevant valid item count as the denominator. These are descriptive counts, not validated scales or measures of severity.

Table 3. *Summary of participant-level barrier and facilitator totals*

Indicator type	Maximum	Mean	Observed range
Barriers	15	7.03	1–14
Facilitators	12	7.28	1–12

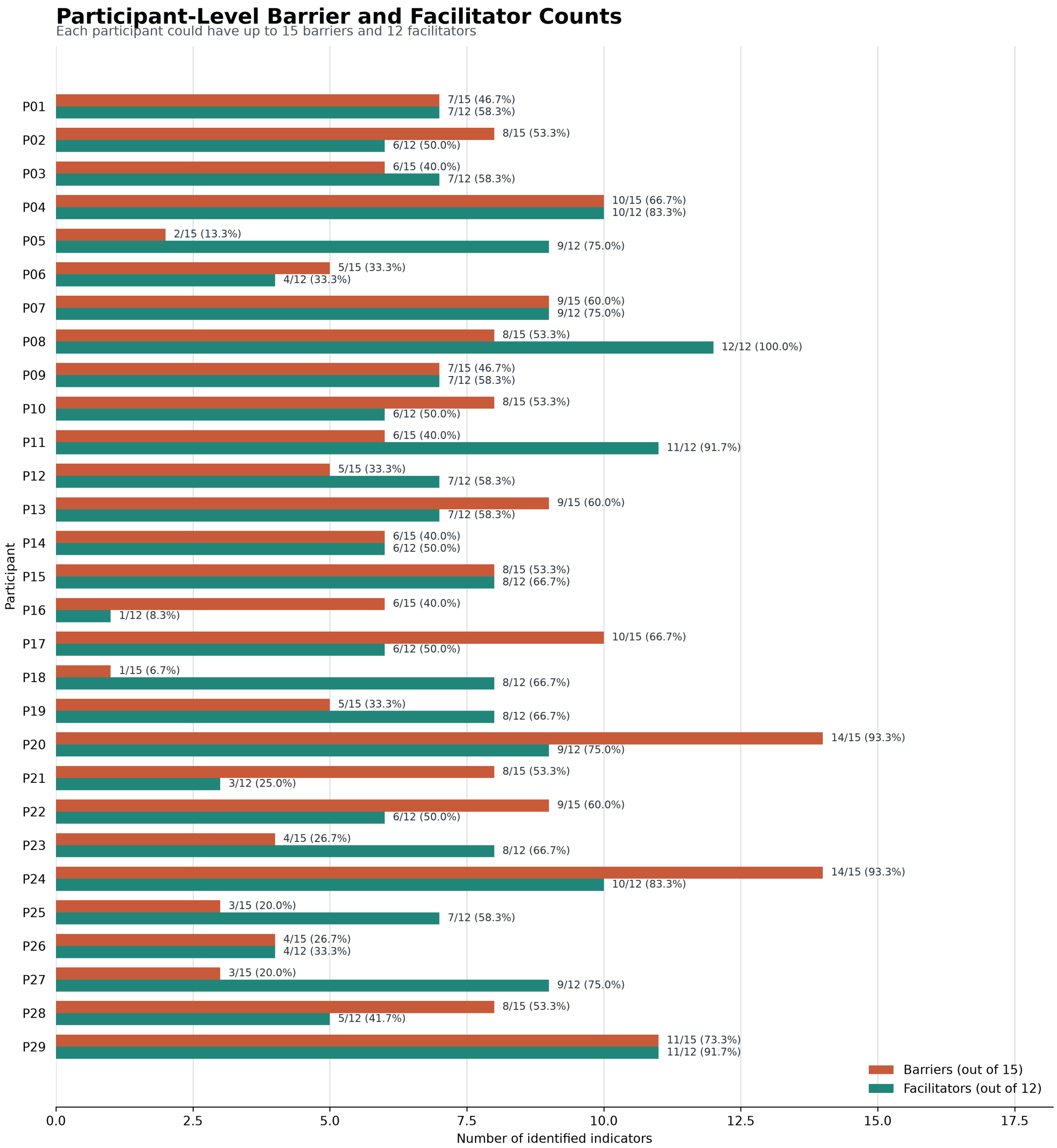

Figure 3. *Paired barrier and facilitator totals for each participant.*

Interpretation

Participants averaged 7.03 barriers and 7.28 facilitators. Barrier counts ranged from 1 to 14 and facilitator counts from 1 to 12, showing considerable heterogeneity. Some participants had high levels of both, demonstrating that facilitators did not necessarily eliminate barriers or unmet needs.

4. CT: Cross-tabulation analysis

Methodology

Each of the four care-needs indicators was cross-tabulated with each of the 27 pathway indicators, producing 108 descriptive 2 × 2 tables. The four cells were neither identified (n00), pathway experience only (n01), unmet need only (n10), and both identified (n11). Available-case percentages and within-unmet-need percentages were calculated; no additional test statistic is required to read the descriptive tables.

Table 4. *High-level summary of pathway experiences within unmet-need groups*

Unmet-need group	n	Leading barriers	Leading facilitators
Communication/information	28	Mental-health concerns: 22 (78.6%); childcare burden/stigma: 21 (75.0%)	Provider compassion/empathy: 27 (96.4%); self-advocacy: 25 (89.3%)
Continuity/affordability	24	Mental-health concerns: 19 (79.2%); childcare burden/stigma: 19 (79.2%)	Provider compassion/empathy: 24 (100%); self-advocacy: 23 (95.8%)
Respect/shared responsibility	22	Mental-health concerns: 17 (77.3%); structural racism: 16 (72.7%)	Provider compassion/empathy: 21 (95.5%); self-advocacy: 21 (95.5%)
Cultural/life-course	26	Mental-health concerns: 22 (84.6%); childcare burden/stigma: 20 (76.9%)	Provider compassion/empathy: 25 (96.2%); self-advocacy: 23 (88.5%)

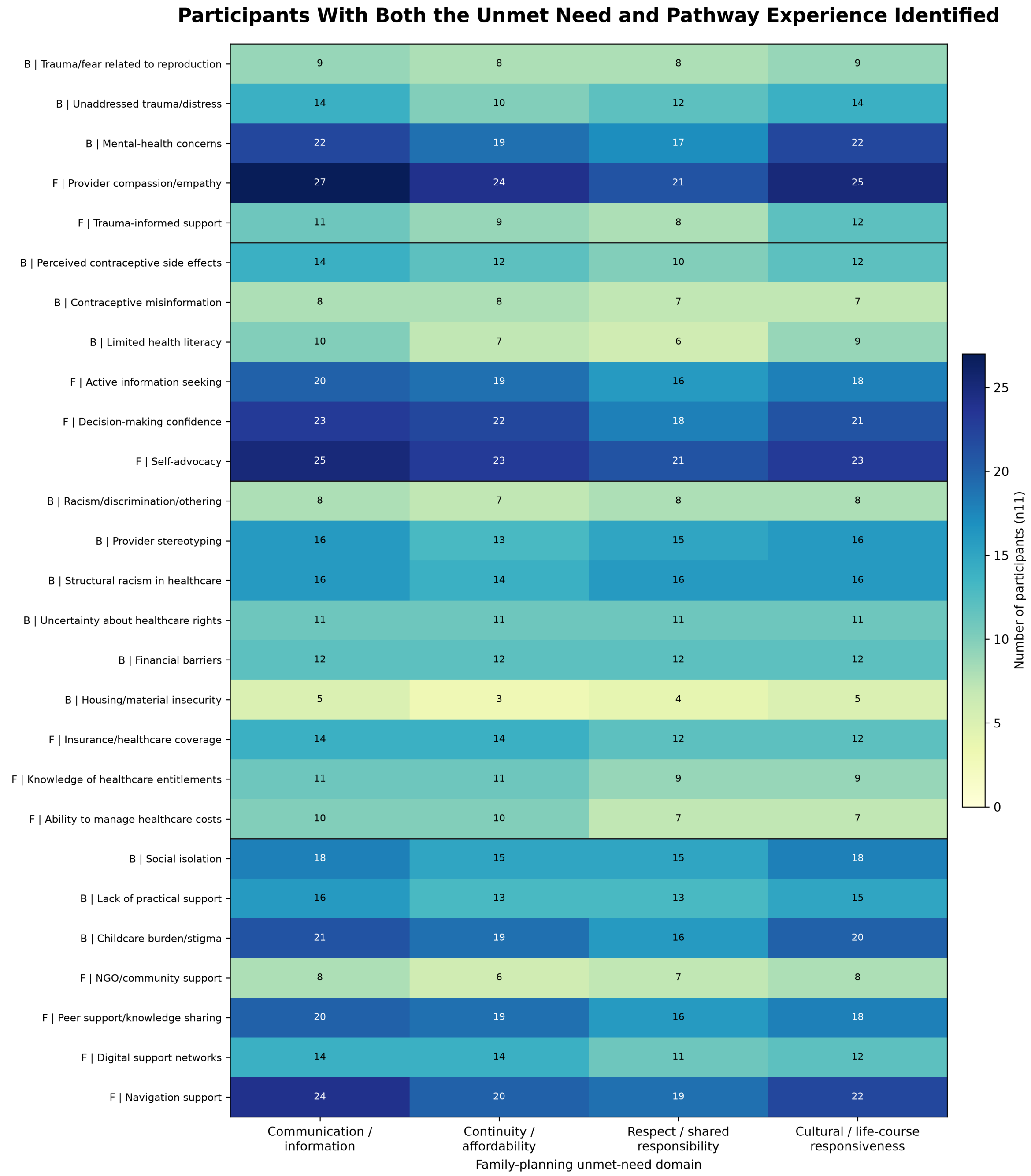

B = barrier; F = facilitator. Descriptive cross-tabulations only; no statistical tests.

Figure 4. *Number of participants with both the unmet need and pathway experience identified.*

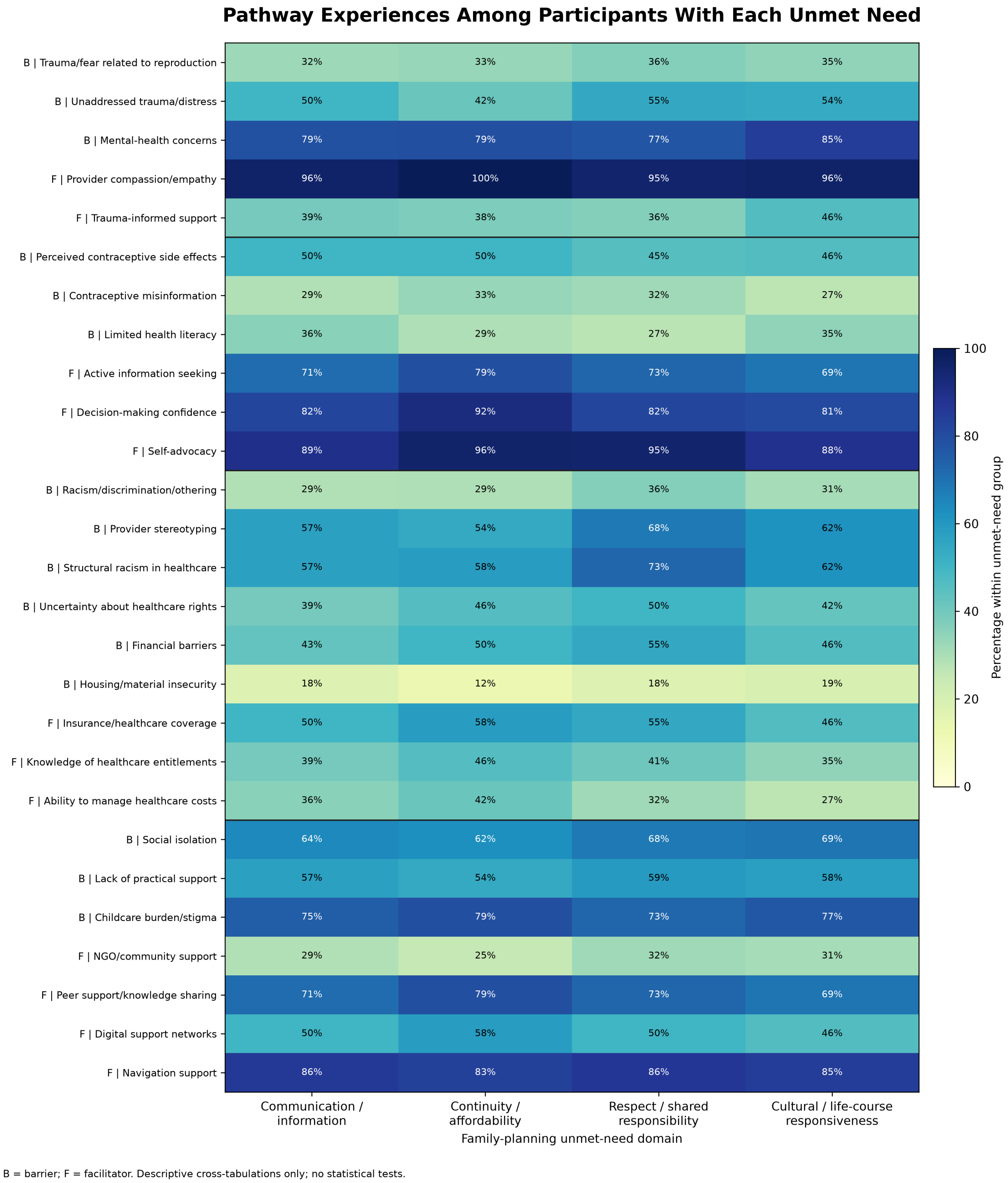

Figure 5. *Percentage with each pathway experience among participants with each unmet need.*

Interpretation

Mental-health concerns and childcare burden/stigma repeatedly appeared across unmet-need groups. Structural racism and provider stereotyping were especially prominent within respect/shared responsibility. Provider compassion, self-advocacy, navigation support, and decision-making confidence were common in every unmet-need group, reinforcing a pattern of simultaneous burden and support. High n11 values are partly driven by the overall prevalence of the unmet needs and should be read as overlap rather than causation.

5. COA: Co-occurrence analysis

Methodology

Co-occurrence configurations were counted when all required determinant, unmet-need, barrier, or facilitator components were identified in the same participant account. Barrier pairs and barrier–facilitator pairings were descriptive. Broader profiles were allowed to overlap; participants could meet more than one definition. Signed phi coefficients in Figure 7 summarize direction and magnitude for binary unmet-need–pathway pairs.

Table 5. *Frequent determinant–need–barrier configurations*

Configuration	Participants
Children + continuity need + childcare burden/stigma	17/29
Children + continuity need + lack of practical support	12/29
Age ≥33 + respect need + mental-health concerns	12/29
≤5 years in Berlin + communication need + social isolation	12/29
Children + continuity need + rights uncertainty	11/29
Children + continuity need + financial barriers	11/29

Table 6. *Frequent determinant–need–facilitator configurations*

Configuration	Participants
Children + continuity need + provider compassion/empathy	21/29
Children + continuity need + decision-making confidence	19/29
Children + continuity need + navigation support	17/29
Limited German + communication need + navigation support	15/29

Table 7. *Most frequent accumulating barrier pairs*

Barrier pair	Participants
Mental-health concerns + childcare burden/stigma	19/29
Social isolation + childcare burden/stigma	17/29
Mental-health concerns + social isolation	16/29
Lack of practical support + childcare burden/stigma	15/29
Provider stereotyping + structural racism	13/29

Table 8. *Recurring, overlapping participant configurations*

Configuration	n	Definition
Cross-pathway accumulation with compensatory support	21	≥2 needs; barriers and facilitators each spanning ≥3 pathways
Trauma and inadequate life-course responsiveness	16	Cultural/life-course need with ≥2 psychological barriers
Disrespect and structural othering	15	Respect need with ≥2 othering/stereotyping/structural-racism barriers
Systems-of-Power burden with individual agency	12	Respect need, ≥3 systems barriers, and ≥2 individual facilitators
Communication constraints with active coping	6	Limited German, communication need, limited literacy, and coping resources
Structural/material vulnerability and multiple barriers	4	Legal/financial vulnerability, continuity need, and material/system barriers
Material/childcare constraints with relational support	3	Children, insecurity, continuity need, childcare/practical barrier, and support

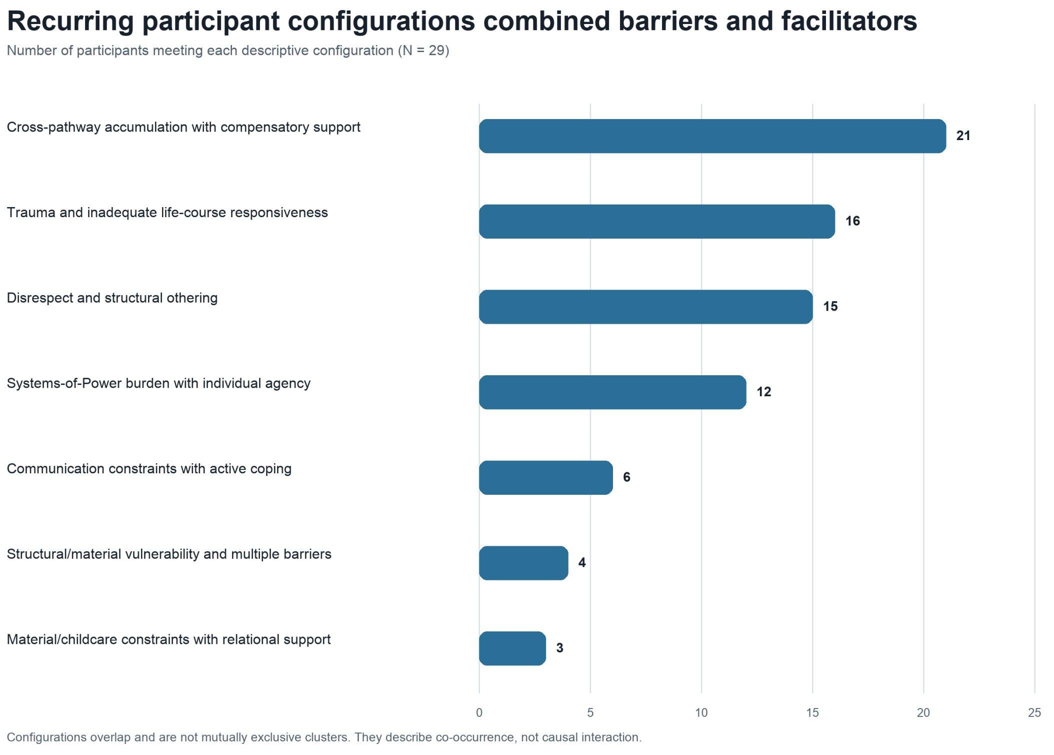

Figure 6. *Frequency of seven recurring, overlapping participant configurations.*

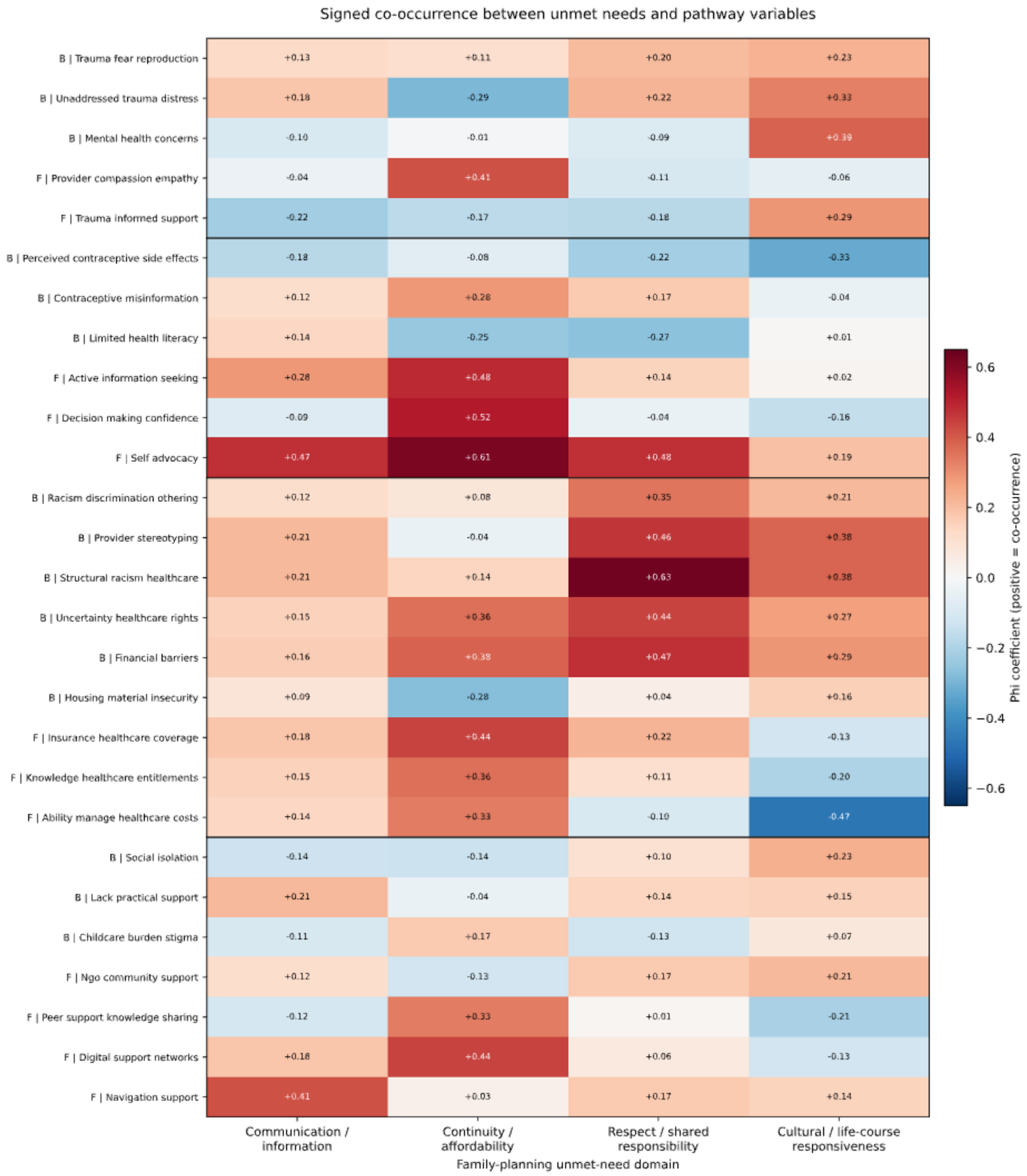

B = barrier; F = facilitator. * q < .05 across all 108 comparisons.

Figure 7. *Signed co-occurrence between unmet needs and pathway variables. Red indicates positive and blue inverse co-occurrence.*

Interpretation

The dominant pattern was cross-pathway accumulation accompanied by compensatory resources. Psychological, caregiving, relational, and structural barriers often accumulated, while compassion, confidence, navigation, peer support, and self-advocacy remained present. The clearest signed binary pattern linked structural racism in healthcare with respect/shared responsibility (phi ≈ 0.63). Configurations overlap and do not define fixed participant types.

6. SC: Spearman correlation analysis

Methodology

Spearman's ρ was used across participants to explore monotonic relationships involving ordered, count, or continuous characteristics. Positive values indicate that higher determinant ranks tended to accompany pathway identification; negative values indicate the reverse. Direction must be read in relation to the determinant coding. Available-case analysis was used, and financial-security comparisons included 20 participants.

Table 9. *Prominent determinant–pathway Spearman correlations*

Characteristic	Pathway indicator	ρ	n	Interpretation
Total pregnancies	Financial barriers	0.564	29	More pregnancies accompanied more financial-barrier coding
Financial security	Decision-making confidence	0.554	20	Greater security accompanied greater confidence
Education	Limited health literacy	−0.551	29	Higher education accompanied less limited-literacy coding
Number of children	Social isolation	0.499	29	More children accompanied more isolation coding
Financial security	Ability to manage healthcare costs	0.477	20	Greater security accompanied cost-management ability
Legal-status insecurity	Insurance/coverage	−0.476	29	Less secure status accompanied lower coverage coding

Table 10. *Prominent determinant–unmet-need Spearman correlations*

Characteristic	Unmet need	ρ	n
Education	Continuity/affordability	0.451	29
Education	Respect/shared responsibility	0.413	29
Financial security	Continuity/affordability	0.363	20

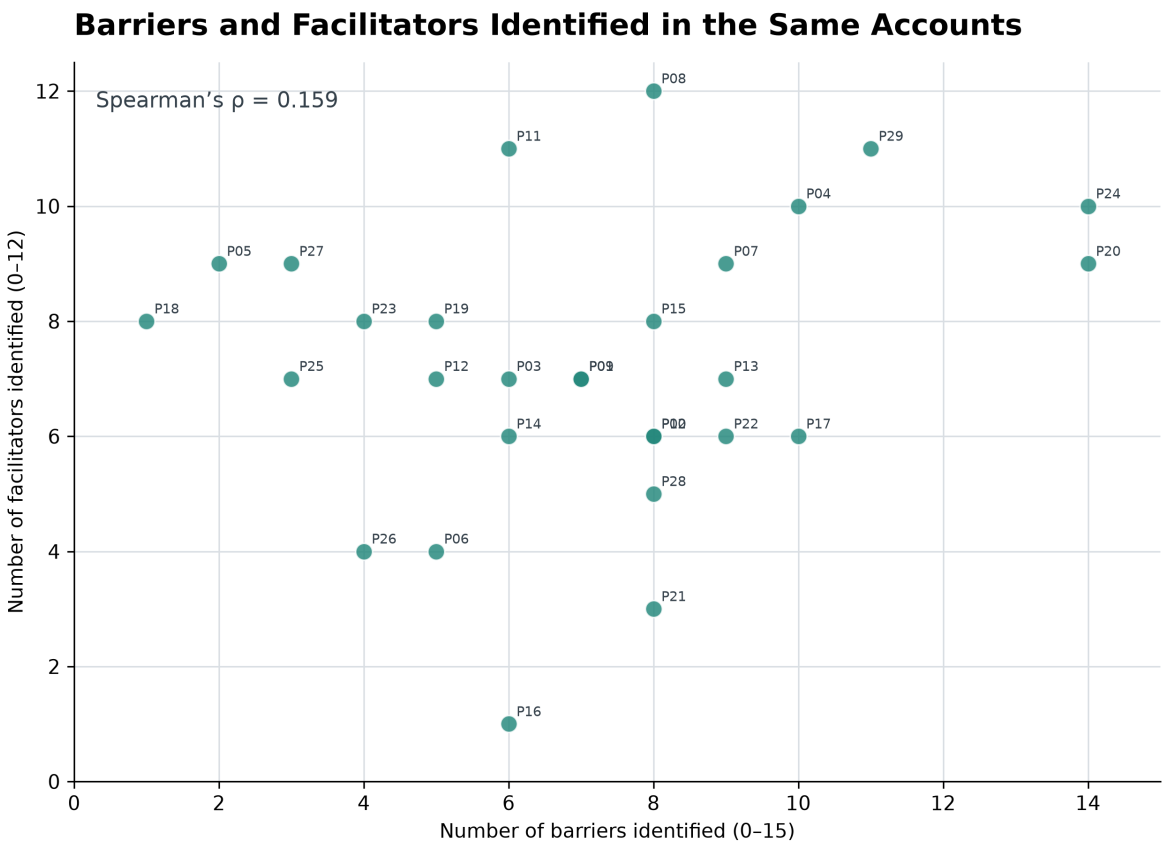

Figure 8. *Participant-level relationship between total barriers and facilitators; Spearman's ρ = 0.159.*

Interpretation

The strongest observed monotonic patterns connected reproductive history with financial barriers, education with lower limited-health-literacy coding, financial security with confidence and cost management, children with social isolation, and legal insecurity with lower coverage. The total barrier–facilitator relationship was weakly positive, consistent with coexistence rather than substitution. These correlations do not establish causal direction.

7. CV: Bias-corrected Cramér's V analysis

Methodology

Bias-corrected Cramér's V was used to summarize the strength of association between nominal participant characteristics and binary matrix indicators. It is derived from contingency-table structure, ranges from 0 to 1, and is non-directional. Category-specific counts are required to determine which categories contribute to a pattern. Sparse or empty cells, especially for country of origin and migration reason, limit stability.

Table 11. *Prominent nominal-characteristic–pathway associations*

Nominal characteristic	Pathway indicator	Role	V	n
Migration reason	Digital support networks	Facilitator	0.570	29
Previously used FP method	Decision-making confidence	Facilitator	0.566	29
Migration reason	Housing/material insecurity	Barrier	0.560	29
Previously used FP method	Financial barriers	Barrier	0.547	29

Table 12. *Prominent nominal-characteristic–unmet-need associations*

Nominal characteristic	Unmet need	V	n
Previously used FP method	Cultural/life-course responsiveness	0.477	29
Country of origin	Continuity/affordability	0.390	29
Previously used FP method	Continuity/affordability	0.347	29

Interpretation

Migration context and family-planning method history showed the largest nominal-category variation in digital support, material insecurity, confidence, financial barriers, and selected unmet needs. Cramér's V does not show direction or identify the responsible category. The results are useful for selecting questions for qualitative follow-up, but they should not be treated as reliable subgroup differences in this small, sparse sample. No dedicated Cramér's V-only graph was available; mixed-measure heatmaps were excluded to avoid mislabeling.

8. Overall conclusion

Across all seven analyses, the participant matrix showed widespread and overlapping family-planning care needs. Psychological distress, childcare burden, social isolation, practical-support gaps, stereotyping, structural racism, financial barriers, and rights uncertainty were recurring constraints. At the same time, provider compassion, self-advocacy, decision-making confidence, navigation support, peer support, and information seeking were frequently present.

The central finding is therefore not a simple barrier-versus-facilitator division. Most participants combined multiple unmet needs and cross-pathway barriers with substantial agency or external support. Structural and material barriers were particularly visible around respect/shared responsibility and continuity/affordability, while individual and relational facilitators often represented resources used under continuing constraint.

The association analyses add exploratory specificity. Spearman correlations highlighted reproductive, educational, financial, legal, and caregiving patterning; bias-corrected Cramér’s V identified variation across migration-context and method-history categories; signed phi coefficients highlighted respect/shared-responsibility alongside systems-of-power barriers. These findings are hypothesis-generating. With only 29 participants, highly prevalent outcomes, missing financial-security data, and sparse nominal categories, the results should not be generalized as population estimates or interpreted causally.

Appendix A. Complete barrier and facilitator indicator table

Pathway	Role	Indicator	Count	%
Psychological Pathways	Barrier	Trauma/fear related to reproduction	9/29	31.0%
Psychological Pathways	Barrier	Unaddressed trauma/distress	14/29	48.3%
Psychological Pathways	Barrier	Mental-health concerns	23/29	79.3%
Psychological Pathways	Facilitator	Provider compassion/empathy	28/29	96.6%
Psychological Pathways	Facilitator	Trauma-informed support	12/29	41.4%
Individual Pathways	Barrier	Perceived contraceptive side effects	15/29	51.7%
Individual Pathways	Barrier	Contraceptive misinformation	8/29	27.6%
Individual Pathways	Barrier	Limited health literacy	10/29	34.5%
Individual Pathways	Facilitator	Active information seeking	20/29	69.0%
Individual Pathways	Facilitator	Decision-making confidence	24/29	82.8%
Individual Pathways	Facilitator	Self-advocacy	25/29	86.2%
Systems of Power	Barrier	Racism/discrimination/othering	8/29	27.6%
Systems of Power	Barrier	Provider stereotyping	16/29	55.2%
Systems of Power	Barrier	Structural racism in healthcare	16/29	55.2%
Systems of Power	Barrier	Uncertainty about healthcare rights	11/29	37.9%
Systems of Power	Barrier	Financial barriers	12/29	41.4%
Systems of Power	Barrier	Housing/material insecurity	5/29	17.2%
Systems of Power	Facilitator	Insurance/healthcare coverage	14/29	48.3%
Systems of Power	Facilitator	Knowledge of healthcare entitlements	11/29	37.9%
Systems of Power	Facilitator	Ability to manage healthcare costs	10/29	34.5%
Relationships and Networks	Barrier	Social isolation	19/29	65.5%
Relationships and Networks	Barrier	Lack of practical support	16/29	55.2%
Relationships and Networks	Barrier	Childcare burden/stigma	22/29	75.9%
Relationships and Networks	Facilitator	NGO/community support	8/29	27.6%
Relationships and Networks	Facilitator	Peer support/knowledge sharing	21/29	72.4%
Relationships and Networks	Facilitator	Digital support networks	14/29	48.3%

Pathway	Role	Indicator	Count	%
Relationships and Networks	Facilitator	Navigation support	24/29	82.8%

Appendix B. Complete participant-level count table

Participant	Barrier count	Barrier %	Facilitator count	Facilitator %
P01	7	46.7%	7	58.3%
P02	8	53.3%	6	50.0%
P03	6	40.0%	7	58.3%
P04	10	66.7%	10	83.3%
P05	2	13.3%	9	75.0%
P06	5	33.3%	4	33.3%
P07	9	60.0%	9	75.0%
P08	8	53.3%	12	100.0%
P09	7	46.7%	7	58.3%
P10	8	53.3%	6	50.0%
P11	6	40.0%	11	91.7%
P12	5	33.3%	7	58.3%
P13	9	60.0%	7	58.3%
P14	6	40.0%	6	50.0%
P15	8	53.3%	8	66.7%
P16	6	40.0%	1	8.3%
P17	10	66.7%	6	50.0%
P18	1	6.7%	8	66.7%
P19	5	33.3%	8	66.7%
P20	14	93.3%	9	75.0%
P21	8	53.3%	3	25.0%
P22	9	60.0%	6	50.0%
P23	4	26.7%	8	66.7%
P24	14	93.3%	10	83.3%

Participant	Barrier count	Barrier %	Facilitator count	Facilitator %
P25	3	20.0%	7	58.3%
P26	4	26.7%	4	33.3%
P27	3	20.0%	9	75.0%
P28	8	53.3%	5	41.7%
P29	11	73.3%	11	91.7%

Appendix C. Complete 108-row descriptive cross-tabulation table

n00 = neither identified; n01 = pathway only; n10 = unmet need only; n11 = both identified. The final column is the percentage with the pathway experience among participants with the unmet need.

Unmet need	R	Pathway indicator	n00	n01	n10	n11	Within unmet %
Communication / information	B	Trauma/fear related to reproduction	1	0	19	9	32.1%
Communication / information	B	Unaddressed trauma/distress	1	0	14	14	50.0%
Communication / information	B	Mental-health concerns	0	1	6	22	78.6%
Communication / information	F	Provider compassion/empathy	0	1	1	27	96.4%
Communication / information	F	Trauma-informed support	0	1	17	11	39.3%
Communication / information	B	Perceived contraceptive side effects	0	1	14	14	50.0%
Communication / information	B	Contraceptive misinformation	1	0	20	8	28.6%
Communication / information	B	Limited health literacy	1	0	18	10	35.7%
Communication / information	F	Active information seeking	1	0	8	20	71.4%
Communication / information	F	Decision-making confidence	0	1	5	23	82.1%
Communication / information	F	Self-advocacy	1	0	3	25	89.3%
Communication / information	B	Racism/discrimination/othering	1	0	20	8	28.6%
Communication / information	B	Provider stereotyping	1	0	12	16	57.1%
Communication / information	B	Structural racism in healthcare	1	0	12	16	57.1%
Communication / information	B	Uncertainty about healthcare rights	1	0	17	11	39.3%
Communication / information	B	Financial barriers	1	0	16	12	42.9%
Communication / information	B	Housing/material insecurity	1	0	23	5	17.9%
Communication / information	F	Insurance/healthcare coverage	1	0	14	14	50.0%

Unmet need	R	Pathway indicator	n00	n01	n10	n11	Within unmet %
Communication / information	F	Knowledge of healthcare entitlements	1	0	17	11	39.3%
Communication / information	F	Ability to manage healthcare costs	1	0	18	10	35.7%
Communication / information	B	Social isolation	0	1	10	18	64.3%
Communication / information	B	Lack of practical support	1	0	12	16	57.1%
Communication / information	B	Childcare burden/stigma	0	1	7	21	75.0%
Communication / information	F	NGO/community support	1	0	20	8	28.6%
Communication / information	F	Peer support/knowledge sharing	0	1	8	20	71.4%
Communication / information	F	Digital support networks	1	0	14	14	50.0%
Communication / information	F	Navigation support	1	0	4	24	85.7%
Continuity / affordability	B	Trauma/fear related to reproduction	4	1	16	8	33.3%
Continuity / affordability	B	Unaddressed trauma/distress	1	4	14	10	41.7%
Continuity / affordability	B	Mental-health concerns	1	4	5	19	79.2%
Continuity / affordability	F	Provider compassion/empathy	1	4	0	24	100.0%
Continuity / affordability	F	Trauma-informed support	2	3	15	9	37.5%
Continuity / affordability	B	Perceived contraceptive side effects	2	3	12	12	50.0%
Continuity / affordability	B	Contraceptive misinformation	5	0	16	8	33.3%
Continuity / affordability	B	Limited health literacy	2	3	17	7	29.2%
Continuity / affordability	F	Active information seeking	4	1	5	19	79.2%
Continuity / affordability	F	Decision-making confidence	3	2	2	22	91.7%
Continuity / affordability	F	Self-advocacy	3	2	1	23	95.8%
Continuity / affordability	B	Racism/discrimination/othering	4	1	17	7	29.2%
Continuity / affordability	B	Provider stereotyping	2	3	11	13	54.2%
Continuity / affordability	B	Structural racism in healthcare	3	2	10	14	58.3%
Continuity / affordability	B	Uncertainty about healthcare rights	5	0	13	11	45.8%
Continuity / affordability	B	Financial barriers	5	0	12	12	50.0%

Unmet need	R	Pathway indicator	n00	n01	n10	n11	Within unmet %
Continuity / affordability	B	Housing/material insecurity	3	2	21	3	12.5%
Continuity / affordability	F	Insurance/healthcare coverage	5	0	10	14	58.3%
Continuity / affordability	F	Knowledge of healthcare entitlements	5	0	13	11	45.8%
Continuity / affordability	F	Ability to manage healthcare costs	5	0	14	10	41.7%
Continuity / affordability	B	Social isolation	1	4	9	15	62.5%
Continuity / affordability	B	Lack of practical support	2	3	11	13	54.2%
Continuity / affordability	B	Childcare burden/stigma	2	3	5	19	79.2%
Continuity / affordability	F	NGO/community support	3	2	18	6	25.0%
Continuity / affordability	F	Peer support/knowledge sharing	3	2	5	19	79.2%
Continuity / affordability	F	Digital support networks	5	0	10	14	58.3%
Continuity / affordability	F	Navigation support	1	4	4	20	83.3%
Respect / shared responsibility	B	Trauma/fear related to reproduction	6	1	14	8	36.4%
Respect / shared responsibility	B	Unaddressed trauma/distress	5	2	10	12	54.5%
Respect / shared responsibility	B	Mental-health concerns	1	6	5	17	77.3%
Respect / shared responsibility	F	Provider compassion/empathy	0	7	1	21	95.5%
Respect / shared responsibility	F	Trauma-informed support	3	4	14	8	36.4%
Respect / shared responsibility	B	Perceived contraceptive side effects	2	5	12	10	45.5%
Respect / shared responsibility	B	Contraceptive misinformation	6	1	15	7	31.8%
Respect / shared responsibility	B	Limited health literacy	3	4	16	6	27.3%
Respect / shared responsibility	F	Active information seeking	3	4	6	16	72.7%
Respect / shared responsibility	F	Decision-making confidence	1	6	4	18	81.8%
Respect / shared responsibility	F	Self-advocacy	3	4	1	21	95.5%
Respect / shared responsibility	B	Racism/discrimination/othering	7	0	14	8	36.4%

Unmet need	R	Pathway indicator	n00	n01	n10	n11	Within unmet %
Respect / shared responsibility	B	Provider stereotyping	6	1	7	15	68.2%
Respect / shared responsibility	B	Structural racism in healthcare	7	0	6	16	72.7%
Respect / shared responsibility	B	Uncertainty about healthcare rights	7	0	11	11	50.0%
Respect / shared responsibility	B	Financial barriers	7	0	10	12	54.5%
Respect / shared responsibility	B	Housing/material insecurity	6	1	18	4	18.2%
Respect / shared responsibility	F	Insurance/healthcare coverage	5	2	10	12	54.5%
Respect / shared responsibility	F	Knowledge of healthcare entitlements	5	2	13	9	40.9%
Respect / shared responsibility	F	Ability to manage healthcare costs	4	3	15	7	31.8%
Respect / shared responsibility	B	Social isolation	3	4	7	15	68.2%
Respect / shared responsibility	B	Lack of practical support	4	3	9	13	59.1%
Respect / shared responsibility	B	Childcare burden/stigma	1	6	6	16	72.7%
Respect / shared responsibility	F	NGO/community support	6	1	15	7	31.8%
Respect / shared responsibility	F	Peer support/knowledge sharing	2	5	6	16	72.7%
Respect / shared responsibility	F	Digital support networks	4	3	11	11	50.0%
Respect / shared responsibility	F	Navigation support	2	5	3	19	86.4%
Cultural / life-course responsiveness	B	Trauma/fear related to reproduction	3	0	17	9	34.6%
Cultural / life-course responsiveness	B	Unaddressed trauma/distress	3	0	12	14	53.8%
Cultural / life-course responsiveness	B	Mental-health concerns	2	1	4	22	84.6%
Cultural / life-course responsiveness	F	Provider compassion/empathy	0	3	1	25	96.2%
Cultural / life-course responsiveness	F	Trauma-informed support	3	0	14	12	46.2%

Unmet need	R	Pathway indicator	n00	n01	n10	n11	Within unmet %
Cultural / life-course responsiveness	B	Perceived contraceptive side effects	0	3	14	12	46.2%
Cultural / life-course responsiveness	B	Contraceptive misinformation	2	1	19	7	26.9%
Cultural / life-course responsiveness	B	Limited health literacy	2	1	17	9	34.6%
Cultural / life-course responsiveness	F	Active information seeking	1	2	8	18	69.2%
Cultural / life-course responsiveness	F	Decision-making confidence	0	3	5	21	80.8%
Cultural / life-course responsiveness	F	Self-advocacy	1	2	3	23	88.5%
Cultural / life-course responsiveness	B	Racism/discrimination/othering	3	0	18	8	30.8%
Cultural / life-course responsiveness	B	Provider stereotyping	3	0	10	16	61.5%
Cultural / life-course responsiveness	B	Structural racism in healthcare	3	0	10	16	61.5%
Cultural / life-course responsiveness	B	Uncertainty about healthcare rights	3	0	15	11	42.3%
Cultural / life-course responsiveness	B	Financial barriers	3	0	14	12	46.2%
Cultural / life-course responsiveness	B	Housing/material insecurity	3	0	21	5	19.2%
Cultural / life-course responsiveness	F	Insurance/healthcare coverage	1	2	14	12	46.2%
Cultural / life-course responsiveness	F	Knowledge of healthcare entitlements	1	2	17	9	34.6%
Cultural / life-course responsiveness	F	Ability to manage healthcare costs	0	3	19	7	26.9%
Cultural / life-course responsiveness	B	Social isolation	2	1	8	18	69.2%
Cultural / life-course responsiveness	B	Lack of practical support	2	1	11	15	57.7%
Cultural / life-course responsiveness	B	Childcare burden/stigma	1	2	6	20	76.9%
Cultural / life-course responsiveness	F	NGO/community support	3	0	18	8	30.8%
Cultural / life-course responsiveness	F	Peer support/knowledge sharing	0	3	8	18	69.2%

Unmet need	R	Pathway indicator	n00	n01	n10	n11	Within unmet %
Cultural / life-course responsiveness	F	Digital support networks	1	2	14	12	46.2%
Cultural / life-course responsiveness	F	Navigation support	1	2	4	22	84.6%
