# Supplementary material for: “Do not leave me behind and do not put me in your box”: family-planning access and care needs among women with migration histories in Berlin, Germany—a mixed-methods study
Source: Reprod Health. 2026 Sep 25;23:175. doi: 10.1186/s12978-026-02450-6 (PMC13613642; doi:10.1186/s12978-026-02450-6)
Supplement: Supplementary file 1 [file 12978_2026_2450_MOESM1_ESM.zip › Supplementary File 6_GRAMMS_Checklist.pdf]

Supplementary File 6. Good Reporting of A Mixed Methods Study (GRAMMS) Checklist

This checklist summarizes how the study addressed the six Good Reporting of A Mixed Methods Study (GRAMMS) recommendations. The study used a **qualitatively driven, emergent sequential mixed-methods design (QUAL → quan → integration)**. Semi-structured interviews constituted the methodological core, followed by a subordinate descriptive participant-level matrix analysis and subsequent integration of the qualitative and descriptive cross-case findings. The study was conducted in **Berlin, Germany, between 17 July and 12 November 2024**.

Item	GRAMMS reporting recommendation	How addressed in this study	Location in manuscript
1	**Explain why mixed methods were needed for the research question.**	The study sought to understand both how women with migration histories experienced family-planning care needs and access and how these experiences were distributed, combined, and varied across participant accounts. Qualitative interviews were required to examine participants' experiences, meanings, care needs, and the structural, psychological, individual, and relational conditions shaping access. The subsequent participant-level matrix enabled systematic descriptive cross-case comparison of the distribution, overlap, coexistence, and heterogeneity of the qualitative findings. The descriptive component was used to complement and extend the qualitative interpretation rather than to test hypotheses or estimate population-level relationships.	**Introduction:**Evidence gap and study aims. **Methods 2.1:**Study Design and Setting. **Methods 2.8:**Data Analysis, particularly Stage 3.
2	**Describe the mixed-methods design, including the purpose, priority, and sequence of the components.**	The study used a **qualitatively driven, emergent sequential mixed-methods design**, with methodological priority given to the qualitative component. Semi-structured interviews were analyzed first using inductive and deductive thematic analysis and mapped to the ETR Health Equity Framework. Findings from the qualitative analysis generated and refined codes that were subsequently transformed into participant-level variables for a subordinate descriptive quantitative component. Qualitative and descriptive findings were then integrated through cross-case comparison, narrative weaving, joint displays, and conceptual-model development. The analytical sequence was therefore **QUAL → quan → integration**. The study was conducted in **Berlin, Germany**, a diverse migrant-receiving urban setting, between 17 July and 12 November 2024.	**Methods 2.1:**Study Design and Setting. **Figure 1:**Mixed-Methods Design and Analytical Sequence. **Methods 2.8:**Data Analysis.
3	**Report sampling, data collection, and analysis procedures for each component.**	The same **29 participant accounts** informed both components, permitting direct participant-level linkage between the qualitative and descriptive analyses. Eligible participants were first-generation women born outside Germany who had migrated from a low- or middle-income country and were residing in Berlin. Recruitment used voluntary-response recruitment followed by eligibility-based purposive screening, with variation sought in migration pathway, country of origin, duration of residence, and reproductive-health experiences. Data were collected through semi-structured interviews conducted in participants' preferred study languages. The qualitative component involved inductive and deductive thematic coding, within- and cross-case comparison, analytic memo writing, and mapping to the ETR Health Equity Framework. For the descriptive component, selected qualitative codes were organized into a participant-by-code matrix comprising **48 variables: 4 family-planning care-need indicators, 17 participant characteristics, and 27 pathway variables, including 15 barriers and 12 facilitators**. Matrix analyses included participant counts and available-case percentages, barrier and facilitator totals, descriptive cross-tabulations, and co-occurrence analyses.	**Methods 2.3:**Study Population and Eligibility Criteria. **Methods 2.4:**Recruitment and Sampling Strategy. **Methods 2.5:**Interview Guide Development and Data Collection. **Methods 2.7:**Data Adequacy. **Methods 2.8, Stages 1–3:**Data Analysis. **Supplementary Files 3–5.**

		Supplementary exploratory analyses used Spearman's rank correlations for ordered or continuous characteristics and bias-corrected Cramér's V for nominal characteristics. These analyses were treated as descriptive and exploratory rather than inferential.	
4	**State where and how integration occurred and who participated in it.**	Integration occurred during the later stages of analysis and interpretation. Matrix entries and selected cross-case patterns were returned to coded excerpts, analytic memos, and, where necessary, original transcripts before interpretation. Qualitative and descriptive findings were integrated through **cross-case comparison, narrative weaving, joint displays, and assessment of convergence, complementarity, divergence or ambiguity, and heterogeneity**. Table 3 presents the joint display of qualitative findings, descriptive matrix patterns, and integrated interpretations. Integration also informed iterative development of the ecological–alignment conceptual model. Integration and interpretation involved the interdisciplinary research team. **BH led the family-planning component and conducted the qualitative and exploratory quantitative analyses; AP and GPK contributed to analysis; NR contributed to qualitative analysis, coding, and interpretation; MF, JL, and JS contributed to interpretation; and all authors critically reviewed and revised the manuscript and its integrated interpretation.**	**Methods 2.8, Stage 5:**Integration and Conceptual-Model Development. **Methods 2.9:**Rigor and Quality Assurance. **Results 3.5:**Integration of Qualitative and Descriptive Cross-Case Findings. **Table 3. Results 3.6:**Integrated Conceptual Model. **Figure 3. Authors' Contributions.**
5	**Describe limitations arising from combining the methods or from the presence of one component alongside the other.**	Several limitations arose from the mixed-methods design. First, transforming complex qualitative accounts into participant-level variables necessarily reduced some contextual nuance. A code of 0 indicated that a finding was **not identified in the interview**, rather than confirming that the corresponding experience was absent; ambiguous or insufficiently supported observations were coded as missing. Second, the small sample (**N = 29**) and sparse or unevenly distributed categories limited the stability and interpretability of exploratory quantitative associations. These findings were therefore treated as descriptive, sample-specific, and hypothesis-generating rather than inferential. Third, the descriptive quantitative variables were derived from the same qualitative interview data rather than from an independent dataset. Consequently, convergence between components should not be interpreted as independent confirmation or validation of the qualitative findings. Instead, the matrix was used to systematize cross-case comparison while retaining the qualitative analysis as the methodological core. More broadly, the purposive and context-specific sample limits population-level generalizability.	**Methods 2.8, Stage 3:**Participant-Level Matrix Construction and Variable Definition. **Methods 2.9:**Rigor and Quality Assurance. **Results 3.4:**Exploratory Matrix Findings. **Discussion:**Strengths and Limitations. **Supplementary File 5.**
6	**Explain the additional insights gained by mixing or integrating the methods.**	Integration provided additional insight into the distribution, coexistence, and heterogeneity of findings across participant accounts. The matrix showed that the principal qualitative care-need domains and Health Equity Framework pathways were widely represented while occurring in different combinations across participants. Integration demonstrated that **unmet care needs, accumulated barriers, agency, and supportive resources could coexist within the same participant accounts**, rather than representing mutually exclusive states. Supportive resources such as self-advocacy, provider compassion, and navigation support could help participants manage constraints without necessarily resolving them. Cross-case analysis also contextualized qualitative findings by showing, for example, that structural racism was concentrated among accounts in which respect and shared responsibility needs were identified and that financial barriers were concentrated among accounts with continuity and affordability needs. Participant characteristics provided contextual signals but did not produce stable or deterministic profiles. These integrated findings informed the study's ecological–alignment conceptual model, which conceptualizes family-planning access and reproductive autonomy in relation to the alignment or misalignment between participants' circumstances, available resources, care needs, and healthcare responses.	**Results 3.4:**Exploratory Matrix Findings. **Results 3.5 and Table 3:**Integration of Qualitative and Descriptive Cross-Case Findings. **Results 3.6 and Figure 3:**Integrated Conceptual Model. **Discussion.**

Abbreviations

ETR, Education, Training, and Research; **GRAMMS**, Good Reporting of A Mixed Methods Study; **LMIC**, low- and middle-income country; **QUAL**, dominant qualitative component; **quan**, subordinate descriptive quantitative component.

Note

The descriptive quantitative component was designed to support **systematic qualitative cross-case comparison and theory development**, rather than statistical hypothesis testing or estimation of population-level associations. Participant-level matrix findings were interpreted in relation to the original qualitative material, and supportive and constraining conditions were allowed to coexist within individual accounts. Full descriptive cross-tabulations, co-occurrence analyses, Spearman's rank correlations, and bias-corrected Cramér's V analyses are reported in Supplementary File 5.

Reporting Guideline Reference

O'Cathain, A., Murphy, E. and Nicholl, J. (2008) 'The quality of mixed methods studies in health services research', *Journal of Health Services Research & Policy*, **13**(2), pp. 92–98. doi: 10.1258/jhsrp.2007.007074.
