# Supplementary material for: “Do not leave me behind and do not put me in your box”: family-planning access and care needs among women with migration histories in Berlin, Germany—a mixed-methods study
Source: Reprod Health. 2026 Sep 25;23:175. doi: 10.1186/s12978-026-02450-6 (PMC13613642; doi:10.1186/s12978-026-02450-6)
Supplement: Supplementary file 1 [file 12978_2026_2450_MOESM1_ESM.zip › Supplementary file 7_Completed COREQ and CCQR checklists .pdf]

Supplementary Table S1. COREQ checklist for the qualitative component of the mixed-methods study

No.	Domain and item	COREQ guide question	Study-specific response	Reporting location
1	**Domain 1: Research team and reflexivity — Personal characteristics: Interviewer/facilitator**	Which author(s) conducted the interviews?	Interviews were conducted by B.H., N.J.A.R., and five trained multilingual research assistants.	**Methods — 2.9 Rigor and Quality Assurance**
2	**Domain 1: Research team and reflexivity — Personal characteristics: Credentials**	What were the researchers' credentials?	B.H. and N.J.A.R. were qualified midwives. The five multilingual research assistants included one researcher with a PhD, one medical doctor, one PhD candidate, and two master's students in International Health.	**Supplementary Table S1**
3	**Domain 1: Research team and reflexivity — Personal characteristics: Occupation**	What was their occupation at the time of the study?	At the time of the study, all interviewers and multilingual research assistants were involved in research at the Institute of International Health. Their roles included midwives, a medical doctor, a PhD-level researcher, a doctoral researcher, and master's students in International Health.	**Supplementary Table S1**
4	**Domain 1: Research team and reflexivity — Personal characteristics: Gender**	What was the gender of the researchers?	Six members of the interviewing team were women, including B.H., N.J.A.R., and four multilingual research assistants. One multilingual research assistant was a man.	**Methods — 2.9 Rigor and Quality Assurance**
5	**Domain 1: Research team and reflexivity — Personal characteristics: Experience and training**	What experience or training did the researchers have?	The interviewing team had backgrounds in midwifery, medicine, international health, and qualitative research and experience in international and multicultural research settings. The lead researchers had personal migration histories, while the wider team included researchers with and without migration histories. Before data collection, interviewers received preparation covering the study objectives, ethical procedures, qualitative interviewing, cultural sensitivity, confidentiality, multilingual communication, and key family-planning and reproductive-health concepts.	**Methods — 2.5 Interview Guide Development and Data Collection; 2.9 Rigor and Quality Assurance; Supplementary Table S1 for additional training detail**
6	**Domain 1: Research team and reflexivity — Relationship with participants: Relationship established**	Was a relationship established before study commencement?	No interviewer or multilingual research assistant had a prior personal relationship with any participant before recruitment or data collection.	**Methods — 2.9 Rigor and Quality Assurance**
7	**Domain 1: Research team and reflexivity — Relationship with participants: Participant knowledge of interviewer**	What did participants know about the researcher?	Before participation, women were informed of the researchers' institutional affiliation, professional and research roles, the purpose of the study, the reasons for conducting the research, and what participation would involve.	**Methods — 2.9 Rigor and Quality Assurance; 2.11 Ethical Considerations**
8	**Domain 1: Research team and reflexivity — Relationship with participants: Interviewer characteristics**	What relevant interviewer characteristics were reported?	Relevant characteristics included gender, migration history, professional background, language, and cultural and professional experiences. The research team reflected on how these characteristics and researchers' assumptions could influence rapport, participant disclosure, data interpretation, and the meanings attributed to participants' accounts.	**Methods — 2.9 Rigor and Quality Assurance**

No.	Domain and item	COREQ guide question	Study-specific response	Reporting location
			Reflexive team discussion, researcher comparison, and deliberate consideration of alternative explanations were used throughout data collection and analysis.	
9	**Domain 2: Study design — Theoretical framework: Methodological orientation and theory**	What methodological orientation and theory underpinned the study?	The study employed a qualitatively driven, emergent sequential mixed-methods design within a **pragmatic research paradigm**, with an **interpretive orientation**guiding the qualitative component. The qualitative analysis used inductive and deductive thematic analysis and was informed by the UNFPA rights-based family-planning framework and the ETR Health Equity Framework. Selected qualitative codes were subsequently organized as participant-level variables for descriptive cross-case analysis, and qualitative and descriptive findings were integrated during conceptual-model development.	**Methods — 2.1 Study Design and Setting; 2.2 Theoretical and Conceptual Frameworks; 2.8 Data Analysis**
10	**Domain 2: Study design — Participant selection: Sampling**	How were participants selected?	Participants were recruited using voluntary-response recruitment followed by eligibility-based purposive screening. Recruitment sought variation in migration pathway, country of origin, duration of residence in Germany, and reproductive-health experiences rather than statistical representativeness. Interested women contacted the research team directly and were screened against predefined eligibility criteria.	**Methods — 2.4 Recruitment and Sampling Strategy**
11	**Domain 2: Study design — Participant selection: Method of approach**	How were participants approached?	Recruitment information was disseminated through community organizations, nongovernmental organizations, social-media channels, community networks, and multilingual study flyers. Community partners included the African Parents Association Berlin-Brandenburg (AfEBB e.V.) and United Action Women and Girls e.V. Interested women contacted the research team directly, after which eligibility was assessed before an interview was arranged.	**Methods — 2.4 Recruitment and Sampling Strategy**
12	**Domain 2: Study design — Participant selection: Sample size**	How many participants were included?	Twenty-nine participants were included in the final analytical sample. Two additional interviews conducted during pilot testing were excluded from the final analytical dataset.	**Methods — 2.3 Study Population and Eligibility Criteria; 2.7 Data Adequacy; Results — 3.1 Participant Characteristics**
13	**Domain 2: Study design — Participant selection: Non-participation**	How many people refused, withdrew, or were excluded, and why?	Three women who contacted the research team were excluded because they had migrated from high-income countries and did not meet the study eligibility criteria. No eligible women declined participation, and no enrolled participants withdrew from the study.	**Methods — 2.3 Study Population and Eligibility Criteria for exclusions; Supplementary Table S1 for refusals and withdrawals**
14	**Domain 2: Study design — Setting: Setting of data collection**	Where was the data collected?	Interviews were conducted in private settings selected in consultation with participants. One interview took place at United Action Women and Girls e.V., four at AfEBB e.V., five at the Institute of International Health at Campus Virchow, six online, and thirteen in participants' homes.	**Methods — 2.4 Recruitment and Sampling Strategy for settings; Supplementary Table S1 for setting-specific interview counts**
15	**Domain 2: Study design — Setting: Presence of non-participants**	Was anyone else present besides the participant and researcher?	One interviewer conducted each interview. No partners, adult family members, organization staff, or other adults were present. In some interviews, participants' infants or young children were present because participants needed to breastfeed or provide childcare.	**Supplementary Table S1**
16	**Domain 2: Study design — Setting: Description of sample**	What were the important characteristics of the sample?	The sample comprised 29 first-generation women who had migrated from low- and middle-income countries and were living in Berlin. Participants represented 17 countries and varied in age, migration pathway, duration of residence, education, employment, marital status, reproductive history, and contraceptive experience.	**Results — 3.1 Participant Characteristics; Tables 1 and 2**

No.	Domain and item	COREQ guide question	Study-specific response	Reporting location
17	**Domain 2: Study design — Data collection: Interview guide**	Were questions, prompts, or guides provided, and was the guide pilot-tested?	A semi-structured interview guide informed by the UNFPA rights-based family-planning framework and the ETR Health Equity Framework was used. The guide addressed seven substantive areas related to family planning and healthcare access. It was pilot-tested in English with two women meeting the study eligibility criteria. Feedback was used to simplify wording, improve transitions and flow, and strengthen probes. The two pilot interviews were excluded from the final analysis.	**Methods — 2.5 Interview Guide Development and Data Collection; 2.6 Pilot Testing and Interview-Guide Refinement; Supplementary File 2**
18	**Domain 2: Study design — Data collection: Repeat interviews**	Were repeat interviews carried out?	No participant was interviewed more than once. Follow-up discussions with two participants concerning preliminary findings did not constitute repeat research interviews or collection of new interview data.	**Supplementary Table S1; Methods — 2.9 Rigor and Quality Assurance for participant follow-up**
19	**Domain 2: Study design — Data collection: Audio/visual recording**	Did the study use audio or visual recording?	All 29 main-study interviews were audio-recorded with participants' consent. No video recording was undertaken.	**Methods — 2.5 Interview Guide Development and Data Collection for audio recording; Supplementary Table S1 for absence of video recording**
20	**Domain 2: Study design — Data collection: Field notes**	Were field notes made during or after interviews?	Interviewers made brief field notes to document issues requiring follow-up and contextual observations relevant to interpretation. The notes supported the analytical process but were not treated as an independent dataset.	**Methods — 2.5 Interview Guide Development and Data Collection**
21	**Domain 2: Study design — Data collection: Duration**	What was the duration of the interviews?	Interviews lasted approximately 60–90 minutes.	**Methods — 2.5 Interview Guide Development and Data Collection**
22	**Domain 2: Study design — Data collection: Data saturation/sample adequacy**	Was data saturation or sample adequacy discussed?	Data collection and preliminary analysis proceeded iteratively. After 25 main-study interviews, the team judged that additional interviews were contributing limited new information relevant to the central research questions. Four additional interviews were conducted to examine thematic adequacy across differing migration pathways and reproductive-health experiences. After 29 interviews, the dataset was judged to provide sufficient thematic depth, variation, and contextual detail to address the study objectives. This was framed as data adequacy rather than statistical or population-level saturation.	**Methods — 2.7 Data Adequacy**
23	**Domain 2: Study design — Data collection: Transcripts returned**	Were transcripts returned to participants for comment or correction?	Interview transcripts were not returned to participants for review or correction.	**Supplementary Table S1**
24	**Domain 3: Analysis and findings — Data analysis: Number of data coders**	How many researchers coded the data?	Two researchers analyzed transcripts in separate NVivo projects, with a third researcher reviewing and comparing the analytical outputs. Differences were discussed to refine coding definitions and interpretations rather than to calculate intercoder reliability.	**Methods — 2.8 Data Analysis; 2.9 Rigor and Quality Assurance**
25	**Domain 3: Analysis and findings — Data analysis: Description of the coding tree**	Was a coding tree or codebook described?	A formal qualitative coding tree was developed and included codes, subcodes, operational definitions, and coding rules. Deductive coding was sensitized by the UNFPA rights-based family-planning framework and ETR Health Equity Framework, while inductive coding allowed additional findings to emerge from participants' accounts. The coding tree and associated definitions are provided in the supplementary materials.	**Methods — 2.8 Data Analysis, Stage 1; Supplementary File 3**

No.	Domain and item	COREQ guide question	Study-specific response	Reporting location
26	**Domain 3: Analysis and findings — Data analysis: Derivation of themes**	Were themes identified in advance or derived from the data?	Themes were developed through a combination of inductive and deductive analysis. The two conceptual frameworks served as sensitizing and organizing tools rather than prespecified causal models. Coding and theme development proceeded iteratively through transcript reading, within- and cross-case comparison, analytic memo writing, and team discussion.	**Methods — 2.2 Theoretical and Conceptual Frameworks; 2.8 Data Analysis, Stages 1–2**
27	**Domain 3: Analysis and findings — Data analysis: Software**	What software was used to manage and analyze the data?	NVivo 12 was used to manage and analyze the qualitative interview data. Microsoft Excel was used to construct and manage the participant-level matrix used for descriptive cross-case analysis.	**Methods — 2.8 Data Analysis for NVivo 12; Supplementary Table S1 for Microsoft Excel**
28	**Domain 3: Analysis and findings — Data analysis: Participant checking**	Did participants provide feedback on the findings?	Preliminary findings were discussed with two participants as an additional source of reflection rather than as a formal validation procedure. Their feedback was considered during interpretation and refinement of the study findings.	**Methods — 2.9 Rigor and Quality Assurance; Supplementary Table S1 for additional detail**
29	**Domain 3: Analysis and findings — Reporting: Quotations presented**	Were participant quotations presented and identified?	Participant quotations are presented throughout the qualitative Results to illustrate themes and support interpretations. Quotations are attributed using pseudonyms and accompanied by selected characteristics, including age and country of origin.	**Results — 3.2 Family Planning Care Needs; 3.3 Pathways Shaping Access**
30	**Domain 3: Analysis and findings — Reporting: Data and findings consistent**	Was there consistency between the data presented and the findings?	Themes and interpretations were repeatedly checked against coded excerpts and original transcripts. Matrix-based findings were also returned to the underlying qualitative material before interpretation, and illustrative quotations were used to demonstrate the relationship between participants' accounts and reported findings.	**Methods — 2.8 Data Analysis; 2.9 Rigor and Quality Assurance; Results — Sections 3.2–3.5**
31	**Domain 3: Analysis and findings — Reporting: Clarity of major themes**	Were the major themes clearly presented?	Four major family-planning care-need themes were clearly identified and presented as separate subsections: communication and information; continuity and affordability; respect and shared responsibility; and cultural and life-course responsiveness. Four Health Equity Framework pathways shaping access were also presented as distinct subsections.	**Results — 3.2 Family Planning Care Needs; 3.3 Pathways Shaping Access**
32	**Domain 3: Analysis and findings — Reporting: Clarity of minor themes and diverse cases**	Were diverse cases or less common perspectives described?	Variation, contrasting experiences, and less common perspectives were retained where supported by participants' accounts. The Results demonstrate heterogeneity in the ways barriers and facilitators were experienced and emphasize that participants with similar characteristics did not necessarily report similar care experiences. Detailed subthemes and supporting codes are provided in the supplementary materials.	**Results — 3.2–3.5, including heterogeneity and cross-case findings; Supplementary materials for detailed subthemes and supporting codes**

Supplementary Table S2. Comprehensive Criteria for Reporting Qualitative Research (CCQR) checklist

No.	CCQR topic	Summary of reporting criterion	How addressed in this study
1	**Title of the paper**	The title should accurately and engagingly reflect the content and identify the study type.	The title identifies the central topic of **family-planning access and care needs**, the study population (**women with migration histories**), the setting (**Berlin, Germany**), and the study design (**mixed-methods study**). The participant quotation in the title reflects the experiential focus of the qualitative component.
2	**Abstract**	Include the research background, purpose, methodology, principal findings, conclusion, and keywords.	The structured abstract reports the background and rationale, study purpose, qualitatively driven mixed-methods design, sample and data-collection procedures, analytical approach, principal care-need domains and Health Equity Framework pathways, implications, and conclusion. Keywords are provided below the abstract.
3	**Introduction**	Clearly describe the study aim, rationale, research problem, relevance, and relationship to existing knowledge.	The Introduction establishes family planning as a human-rights and health-equity issue, summarizes existing evidence on family-planning access among women with migration histories, identifies limitations in previous German and international research, defines the explanatory research gap, and states the study aim and two specific objectives.
4	**Methodology**	Describe the design, theoretical framework and research paradigm, methodological rationale, population and eligibility criteria, setting, sampling, researcher–participant relationship, data collection, analytical procedures, data management, reflexivity, reporting standards, and transparency of study procedures.	The Methods describe a **qualitatively driven, emergent sequential mixed-methods design** situated within a **pragmatic research paradigm with an interpretive orientation**. The UNFPA rights-based family-planning framework and ETR Health Equity Framework are described and justified. The study setting, population, sample size, inclusion and exclusion criteria, recruitment and purposive screening, interviewer characteristics and relationships with participants, interview-guide development and pilot testing, multilingual interviewing, audio recording, field notes, verbatim transcription, translation, pseudonymization, thematic analysis, coding tree, analytical software, numbers of coders, participant-level matrix construction, cross-case analysis, mixed-methods integration, and conceptual-model development are reported. Reflexivity and analytical transparency are described in detail, and GRAMMS, COREQ, and CCQR reporting guidance is identified.
5	**Trustworthiness**	Explain procedures used to establish credibility, transferability, dependability, confirmability, and authenticity.	Credibility was supported through iterative analysis, researcher comparison, reflexive discussion, repeated return to transcripts and coded excerpts, and discussion of preliminary findings with two participants. Transferability was supported through detailed description of the setting, sample, recruitment, and contextual variation. Dependability was strengthened through documented coding procedures, operational definitions, shared analytical memos, and an audit trail. Confirmability was supported through reflexivity, researcher comparison, consideration of alternative explanations, and checking matrix findings against qualitative material. Authenticity was supported by retaining variation, contrasting experiences, and less common perspectives.
6	**Ethical consideration**	Report ethical approval, confidentiality/anonymity protections, informed consent, and consent procedures.	Ethical approval was obtained from the Ethics Committee of Charité–Universitätsmedizin Berlin (**EA4/076/23**), with additional permissions from participating recruitment organizations. Written informed consent was obtained before participation and included consent for audio recording and research use of the data. Voluntariness, withdrawal rights, confidentiality, pseudonymization, secure data storage, restricted access, GDPR requirements, and consent for publication of anonymized quotations are described.
7	**Results**	Clearly present the findings, including major and minor themes, narrative explanation, supporting quotations or field material, and appropriate visual presentation.	The Results describe participant characteristics, four major family-planning care-need themes, four Health Equity Framework pathways, descriptive cross-case findings, integrated mixed-methods findings, and the conceptual model. Themes are supported by participant quotations identified using pseudonyms and selected characteristics. Detailed subthemes and supporting codes are provided in the supplementary materials. Tables, a joint display, pathway visualization, and the integrated

No.	CCQR topic	Summary of reporting criterion	How addressed in this study
			conceptual model support clear presentation of the findings. Field notes informed interpretation but were not treated as an independent analytical dataset.
8	**Discussion**	Interpret the findings, compare them with existing literature and theory, consider bias, heterogeneity and alternative explanations, and describe implications, transferability, strengths, limitations, and contribution to the field.	The Discussion interprets the findings in relation to meaningful access, reproductive autonomy, agency, structural and migration-related conditions, communication, respectful care, and health equity. Findings are compared with rights-based approaches and previous migration and reproductive-health literature. Selection considerations and sample heterogeneity are acknowledged. Alternative interpretations are considered, including that several care needs may reflect broader healthcare-system conditions rather than being unique to women with migration histories. The Discussion explicitly addresses transferability, implications, study contribution, and methodological limitations.
9	**Conclusion**	Conclusions should arise from the analysis and interpretation and clearly describe implications.	The Conclusion synthesizes the integrated findings and conceptualizes family-planning access as dependent on alignment among women's circumstances, preferences, resources, and healthcare responses. It emphasizes the coexistence of agency and structural constraints and identifies implications for health-system responsiveness, equitable access, informed choice, and reproductive autonomy.
10	**Strengths and limitations**	Describe study strengths and limitations and indicate the value and boundaries of the findings.	The manuscript identifies strengths including the qualitatively driven mixed-methods design, multilingual data collection, diversity of countries of origin and migration pathways, interdisciplinary research team, reflexive analysis, participant checking, and integration of qualitative and descriptive findings. Limitations include possible recruitment bias, sample composition, eligibility-related sampling boundaries, multilingual translation issues, small sample size, researcher and participant subjectivities, and incompletely captured intersecting characteristics. Findings are explicitly framed as context-specific rather than statistically generalizable.
11	**Recommendations**	Provide recommendations for future research and relevant practice or policy.	Separate **Practice, Research, and Policy Implications** are provided. Recommendations include rights-based, culturally responsive, trauma-informed and anti-discriminatory care; improved multilingual communication, professional interpretation, and navigation support; reduction of financial and administrative barriers; validation and testing of the conceptual model and study-derived measures; larger and more diverse studies; longitudinal and participatory research; and monitoring of equitable access using social determinants of health and context-specific equity indicators.
12	**Funding**	Identify financial and non-financial support, the role of funders, and any support for authorship or publication.	Institutional support was provided by **Charité–Universitätsmedizin Berlin**, and participant vouchers were funded by the Institute of Midwifery Science. The manuscript states that institutional support did not influence study design, data collection, analysis, interpretation, or manuscript preparation. **No financial support was received for authorship, manuscript preparation, or publication.**
13	**References**	Identify information sources and citations and provide relevant appendices or supplementary materials.	Relevant literature and methodological sources are cited throughout the manuscript. Supplementary materials provide supporting documentation including recruitment materials, the interview guide and consent form, coding tree, codebook and variable definitions, exploratory analyses, and completed GRAMMS, COREQ, and CCQR reporting checklists.
14	**Conflict of interest**	Declare potential competing interests and explain how any potential influence was managed.	The authors declare that they have **no competing interests**. Because no competing interests were identified, no additional conflict-management procedures were required.
